# Shotgun sequencing of the vaginal microbiome reveals both a species and functional potential signature of preterm birth

**DOI:** 10.1038/s41522-020-00162-8

**Published:** 2020-11-12

**Authors:** Conor Feehily, David Crosby, Calum J. Walsh, Elaine M. Lawton, Shane Higgins, Fionnuala M. McAuliffe, Paul D. Cotter

**Affiliations:** 1 Teagasc Food Research Centre, Moorepark, Fermoy, Cork, Ireland; 2 APC Microbiome Ireland, University College Cork, Cork, Ireland; 3 UCD Perinatal Research Centre, Obstetrics and Gynaecology, School of Medicine, University College Dublin, National Maternity Hospital, Dublin, 2 Ireland

**Keywords:** Microbiome, Microbial ecology

## Abstract

An association between the vaginal microbiota and preterm birth (PTB) has been reported in several research studies. Population shifts from high proportions of lactobacilli to mixed species communities, as seen with bacterial vaginosis, have been linked to a twofold increased risk of PTB. Despite the increasing number of studies using next-generation sequencing technologies, primarily involving 16S rRNA-based approaches, to investigate the vaginal microbiota during pregnancy, no distinct microbial signature has been associated with PTB. Shotgun metagenomic sequencing offers a powerful tool to reveal community structures and their gene functions at a far greater resolution than amplicon sequencing. In this study, we employ shotgun metagenomic sequencing to compare the vaginal microbiota of women at high risk of preterm birth (*n* = 35) vs. a low-risk control group (*n* = 14). Although microbial diversity and richness did not differ between groups, there were significant differences in terms of individual species. In particular, *Lactobacillus crispatus* was associated with samples from a full-term pregnancy, whereas one community state-type was associated with samples from preterm pregnancies. Furthermore, by predicting gene functions, the functional potential of the preterm microbiota was different from that of full-term equivalent. Taken together, we observed a discrete structural and functional difference in the microbial composition of the vagina in women who deliver preterm. Importance: with an estimated 15 million cases annually, spontaneous preterm birth (PTB) is the leading cause of death in infants under the age of five years. The ability to accurately identify pregnancies at risk of spontaneous PTB is therefore of utmost importance. However, no single cause is attributable. Microbial infection is a known risk factor, yet the role of vaginal microbes is poorly understood. Using high-resolution DNA-sequencing techniques, we investigate the microbial communities present in the vaginal tracts of women deemed high risk for PTB. We confirm that *Lactobacillus crispatus* is strongly linked to full-term pregnancies, whereas other microbial communities associate with PTB. Importantly, we show that the specific functions of the microbes present in PTB samples differs from FTB samples, highlighting the power of our sequencing approach. This information enables us to begin understanding the specific microbial traits that may be influencing PTB, beyond the presence or absence of microbial taxa.

## Introduction

Preterm birth (PTB) is the leading cause of mortality in infants under the age of 5 years^1^. The rate of spontaneous PTB, defined as the onset of labour and subsequent delivery before 37 weeks of gestation, is reported to be at one in ten pregnancies (~15 million PTB annually) worldwide^2,3^. In addition to increased mortality, prematurity is associated with significant morbidity in terms of chronic lung disease, increased rates of neurodevelopmental delay, and long-term health problems^4–6^. The economic burden through healthcare costs related to prematurity is estimated to be $26 billion in the United States alone^7^.

Several studies have investigated links between maternal health status including diet^8^, smoking^9^, obesity^10^, stress^11^, age^12^, and previous history of obstetric complications^13^ as indicators of PTB. Microbial infection, specifically urinary tract infections, vaginitis, bacterial vaginosis (BV), and even periodontal disease have also been associated with an increased risk of spontaneous preterm delivery^14–16^. The composition of the microbiota of the mother, and in particular the mother’s vaginal microbiome, has more recently been linked to spontaneous PTB^3^. In general, the vaginal microbiota is stable throughout pregnancy with the dominant species rarely changing^17,18^; however, biogeography and ethnicity can play a considerable role in determining the apparent ‘normal’ microbiota^19^. A study of 90 women, mainly of African American decent, found that there was no association between the vaginal microbiota and PTB^20^. In contrast, DiGiulio et al.^21^ found that changes in the vaginal populations of Caucasian women were correlated with PTB. Further studies have provided additional evidence that race is a key factor when identifying patterns linking PTB with vaginal microbiota composition^22,23^.

The common means of categorizing the vaginal microbiome has been to group them into community state types (CSTs). CSTs dominated by *Lactobacillus* species have generally been associated with positive health states for the woman^24^. In contrast, CST-4, which is reduced in lactobacilli and contains an increased abundance of mixed species, has been associated with poorer health outcomes. These mixed species, including *Gardnerella vaginalis* (assigned as *Bifidobacterium vaginale* in the Genome Taxonomy Database (GTDB)), *Atopobium vaginae* (assigned as *Fannyhessea vaginae* in the GTDB*)*, *Mobiluncus* sp., *Prevotella* sp., and others, are most often associated with BV. Multiple studies have reported a twofold increased risk for PTB in women suffering from BV^25,26^. Despite this, large cohort studies have reported that CST-4 can also be present in a high percentage of the healthy population with no symptoms of BV^27^. Indeed, *B. vaginale* has also been frequently isolated from asymptomatic women^28^.

Determining a role for the vaginal microbiota in influencing pregnancy outcome has been limited by the ability to fully differentiate taxonomic groups. This resolution may prove important if discrete communities, species, or strains prove to represent a risk factor. In light of this, it is important to consider that the use of standard 16S rRNA targeted amplicon sequencing has been reported to under-report certain subgroups of *B. vaginale*^29^, while targeting the *cpn60* gene, has shown an improvement in terms of identifying *Bifidobacterium*^30^. Shotgun metagenomics presents an opportunity to overcome many of the limitations of amplicon sequencing, with the additional benefit of understanding the functional potential of a community. An increasing number of studies have now used this approach to elucidate the vaginal microbial communities. In particular, functional distinctions among different metagenomic assembled *B. vaginale* isolated from the same sample^31^ and an ability to sub-speciate important taxa^32^ provide examples for the power of this approach.

In a cohort of women with a predisposed risk for PTB, we aimed to use shotgun metagenomics to distinguish any vaginal microbial signatures different to a low-risk control group.

## Results

### Participant data

A total of 57 participants with singleton pregnancy were successfully recruited over the study period, 20 at low risk of PTB and 37 with risk factors for PTB. Multiple swabs were collected from some women depending on clinic visit frequency, accounting for 89 total samples. To control for multiple sampling of some woman and different trimester timepoints, we focused our analysis on single samples for each woman in the second trimester of pregnancy (*n* = 49). Of these, 8 pregnancies ultimately were preterm (PTB) and the remaining 41 were full-term birth (FTB). In addition, for the 35 participants initially considered at risk of PTB, 7 women delivered before 37 weeks’ gestation and were grouped into the PTB group (risk_PTB). The remainder were grouped into a risk but full-term group (risk_FTB). For the 14 women at low risk of PTB, 13 delivered at term (no-risk_FTB). The single sample from this control group that delivered prior to 37 weeks was subsequently provided a distinct group of no-risk_PTB. Within the PTB samples, six deliveries were late preterm (32–37 weeks), one was a moderate preterm (31.7 weeks), and one was a preterm at 26 weeks (Table 1). None of the women had preterm premature rupture of membranes. There were no significant differences in the age, race, body mass index, or smoking status of the participants by either the grouping of of risk_PTB, risk_FTB, no-risk_FTB, and no-risk_PTB or the FTB and PTB grouping. Patient demographics are outlined in Table 1. There was a lower birth weight for infants from the PTB compared to FTB (2230.62 g vs. 3646.12 g, respectively; *p* < 0.001 Student’s *t*-test).

**Table 1:** Descriptive statistics of study participants.

	Risk		Control	
	risk_PTB^a^ (*n* = 7)	risk_FTB^b^ (*n* = 28)	non-risk PTB^c^ (*n* = 1)	non-risk FTB^d^ (*n* = 13)
Age (years)	31.43	33.14	42	34.08
BMI (kg/m^2^)	26.07	25.41	32.02	23.6
Gestational age at delivery (weeks)	33.06	39.71	36.7	39.67
<28 Weeks	1	0	0	0
28–32 Weeks	0	0	0	0
32–37 Weeks	6	0	1	1
Ethnicity				
White Irish	6	24	1	12
White Caucasian	1	2	0	0
Asian	0	1	0	0
African	0	1	0	1
Smokers	1	3	0	0
Birth weight	2143.57	3666.43	2840	3602.38
LLETZ	2	9	0	0
Previous PTB	6	19	0	0
ABX within previous 6 months	3	6	0	2

^a^risk_PTB preterm birth refers to those women who delivered prior to 37 weeks’ gestation.

^b^risk_FTB refers to those women with risk factors for preterm birth but who delivered full term.

^c^non-risk PTB refers to low-risk women who delivered prior to 37 weeks’ gestation.

^d^non-risk FTB refers to low-risk women who delivered at term.

### Taxonomic analysis

**Fig. 1:**
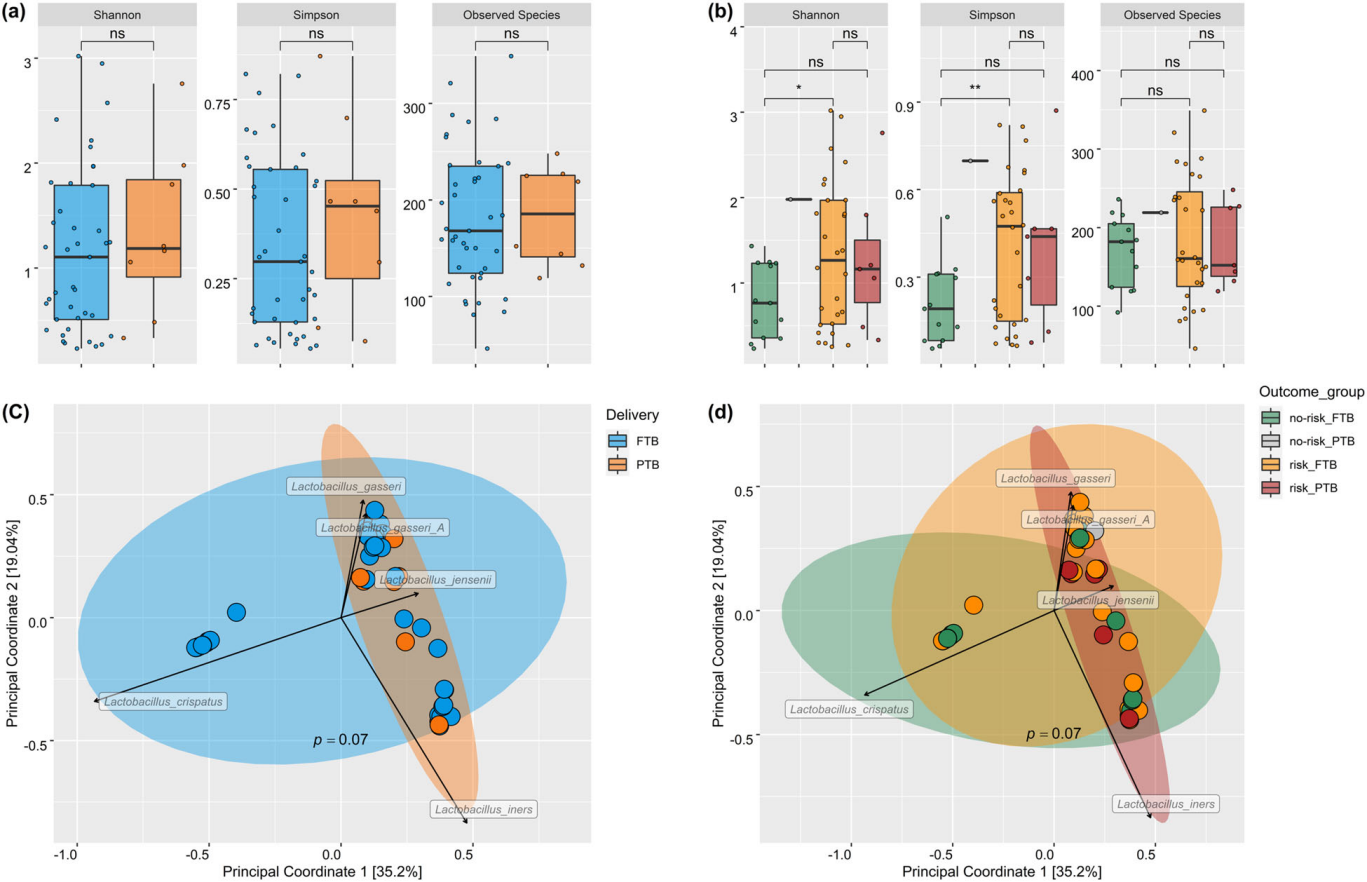
Species diversity between study groups. α-Diversity comparison as measured by Shannon and Simpson index, including the total observed species for either delivery outcome (**a**) or risk grouping (**b**). Significant differences as calculated by Wilcoxon’s test are noted with *p < 0.05 or **p < 0.02. The species level community dissimilarity as measured by Bray–Curtis and visualized using PCoA for either delivery outcome (**c**) or risk grouping (**d**). Ellipses are generated using stat_ellipse function in R. Significance of group dissimilarity as calculated by PERMANOVA is identified by the given *p*-value. The top five lactobacilli across all samples are labelled to highlight drivers of variation for clusters with black arrows to indicate the directionality.

Following quality filtering, a mean of 828,950 high-quality microbial sequencing reads were obtained per sample. The median number of observed species for either the FTB or PTB groups was 168 and 185.5, respectively (Fig. 1a), with the highest number of species observed at 15 for a risk_PTB sample. There was no significant difference in α-diversity measure between either the FTB or PTB groups (Fig. 1a); however, there was an increased diversity of the risk_FTB group compared to the no-risk_FTB group using both Shannon and Simpson measures (Fig. 1b; *p* = 0.01 and *p* = 0.003, respectively). In terms of β-diversity, the risk_PTB communities were significantly dissimilar from both the no-risk_FTB and risk_FTB samples (Fig. 1b; *p* = 0.02).

**Fig. 2:**
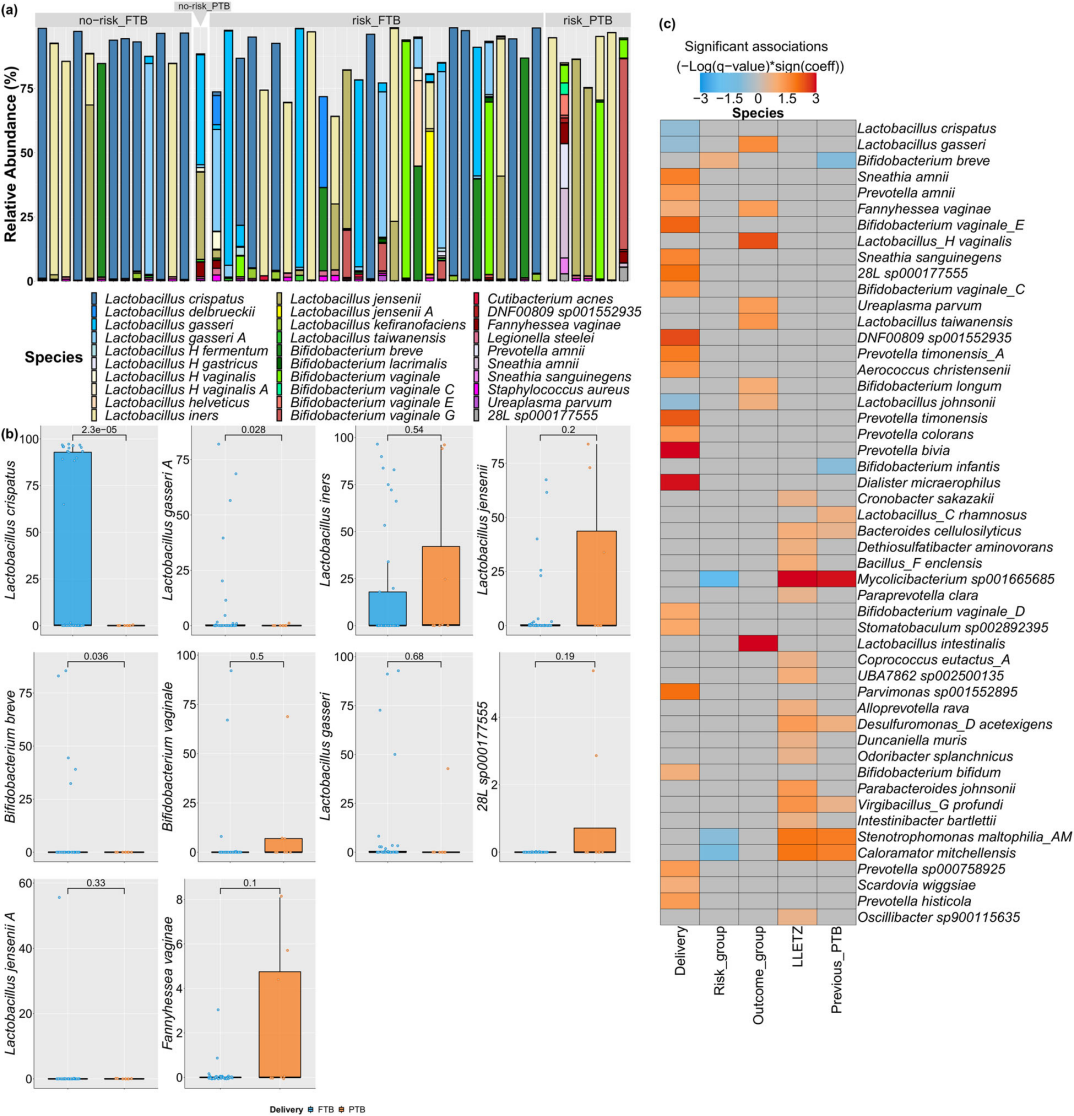
Species composition across study groups. **a** The relative abundance for the top 30 species across all samples for each of the four risk groupings. **b** Comparitive analysis within the delivery outcomes for the ten most abundant species across all samples. Significant differences were calculated using Student’s *t*-test. **c** MaAsLiN analysis correlating species multiple metadata fixed effects. Heatmap displays the top 50 species with a significant assoaction to either fixed effect with *q-*value < 0.25.

Taxonomic classification revealed that lactobacilli were dominant across all groups at 59.13% mean relative abundance (Fig. 2a), with a greater proportion observed in full-term pregnancies (61.86%) compared to preterm (45.11%). This dominance was greatest in the no-risk_FTB group (71.32%) where both *Lactobacillus crispatus* (50.86%) and *Lactobacillus iners* (21.27%) were dominant. Within the risk_FTB group, *L. crispatus* (25.83%)*, L. iners* (14.83%), *Lactobacillus gasseri* (11.70%), and *L. gasseri* A (7.30%) were the dominant species detected. *L. crispatus* was never >0.03% relative abundance in the risk_PTB group. In addition, *L. gasseri* was only detected in two samples in the risk_PTB group, accounting for just 0.01% mean relative abundance. Although *L. iners* (30.82%) accounted for a greater proportion of the mean population in the risk_PTB group compared to both FTB groups, this increase was not statistically significant. Due to the large variation in both the abundance and number of species observed between samples, *L. crispatus* (*p* < 0.001), *L. gasseri* (*p* = 0.028), and *Bifidobacterium breve* (*p* = 0.036) were the only species that significantly differed between full-term and preterm groups, with a higher respective mean relative abundance (Fig. 2b). A significant negative correlation was observed between PTB samples and both *L. crispatus* and *L. gasseri* (*p*-value 0.03 and 0.03, respectively), and after correcting for multiple comparisons, these *q*-values were below than 0.25 (Fig. 2c, Table 2, and Supplementary Table 2). Overall, the dominance of BV-associated bacteria was low across all samples. Using the GTDB database, *B. vaginale* (this database assigns *G. vaginalis* as *B. vaginale*) was assigned as multiple sub-species. The two most abundant BV-associated species across all samples were *B. vaginale* and *B. vaginale*_G; however, neither were significantly increased in either FTB or PTB samples (Fig. 2b). A significant correlation with PTB samples was observed for *B. vaginale_D*, *_E*, and *_F* (Fig. 2c, Table 2, and Supplementary Table 2; *q*-value 0.097, 0.006, and 0.002, respectively). In addition, *F. vaginae* and *F. vaginae*_A (this database assigns *A. vaginae* as *F. vaginae*) were positively correlated with PTB samples (*q*-value 0.137 and 0.008, respectively; Fig. 2c, Table 2, and Supplementary Table 2).

**Table 2:** MaAsLiN2 multivariate correlation analysis of most abundant microbial species and sample groupings.

Species	Group	Value	Coef	stderr	*N*	*p-*Value	*q*-Value
*B. breve*	Previous_PTB	Yes	−1.8149	0.7393	49	0.0182	0.1366
*B. breve*	Risk_group	Risk	1.8357	0.8000	49	0.0267	0.1810
*B. vaginale_C*	Delivery	PTB	0.6202	0.2028	49	0.0038	0.0353
*B. vaginale_E*	Delivery	PTB	0.7502	0.2001	49	0.0005	0.0064
*DNF00809 sp001552935*	Delivery	PTB	0.6983	0.1710	49	0.0002	0.0032
*F. vaginae*	Outcome_group	no-risk_PTB	1.3714	0.4852	49	0.0071	0.0580
*F. vaginae*	Delivery	PTB	0.4585	0.1870	49	0.0184	0.1373
*L. crispatus*	Delivery	PTB	−1.6480	0.7347	49	0.0301	0.2030
*L. gasseri*	Outcome_group	no-risk_PTB	4.5300	1.4246	49	0.0027	0.0266
*L. gasseri*	Delivery	PTB	−1.2000	0.5492	49	0.0344	0.2284
*Lactobacillus taiwanensis*	Outcome_group	no-risk_PTB	2.6198	0.8731	49	0.0045	0.0407
*Lactobacillus_H vaginalis*	Outcome_group	no-risk_PTB	2.6170	0.6470	49	0.0002	0.0036
*Prevotella amnii*	Delivery	PTB	0.0496	0.0173	49	0.0062	0.0513
*Sneathia amnii*	Delivery	PTB	0.2996	0.0878	49	0.0014	0.0160
*Sneathia sanguinegens*	Delivery	PTB	0.5417	0.1629	49	0.0018	0.0193
*Ureaplasma parvum*	Outcome_group	no-risk_PTB	1.5612	0.5485	49	0.0067	0.0553
*28 L sp000177555*	Delivery	PTB	0.8062	0.2222	49	0.0008	0.0090

**Fig. 3:**
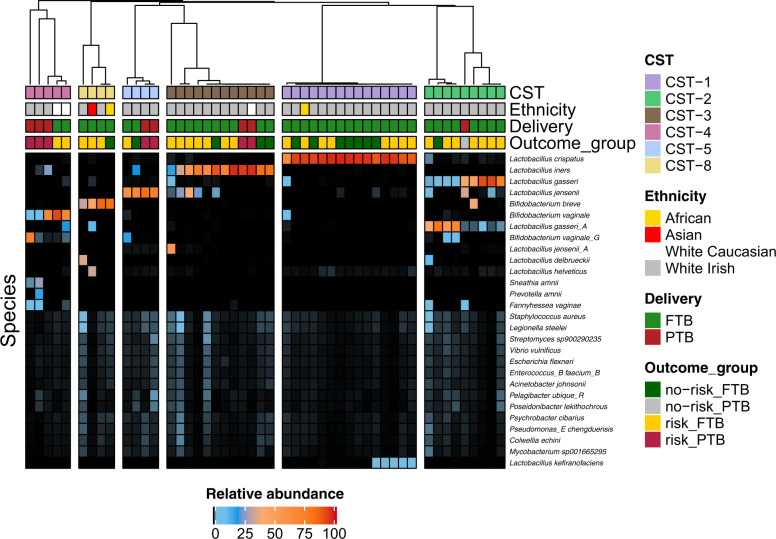
Community state types of all samples. Relative heatmap intensity comparison for all species with an across-sample mean relative abundance > 0.2, with sample clustering of similar samples by Pearson’s clustering. Community state types are defined based on the most abundant species per sample and are indicated by the top bar of the figure.

Resolution of the microbial species into CSTs revealed that six distinct CSTs were present across all swabs (Fig. 3). CSTs were determined by the most dominant species in a cluster as previously described^33^ with the definition of CST-8 according to Brooks et al.^24^. CST-1, dominated by *L. crispatus*, had a weak association overall with delivery outcome (Supplementary Table 1; *p* = 0.09). There was a weak association between samples from the risk_PTB groups and CST-5 (Supplementary Table 1; *p* = 0.09). There were five observations of the BV-associated CST-4, three of which were from the risk_PTB group. This CST had a positive association with samples from the risk_PTB group (*p* = 0.02). Neither CST-3 nor CST-8 were associated with any pregnancy outcome.

### Functional pathways analysis

**Fig. 4:**
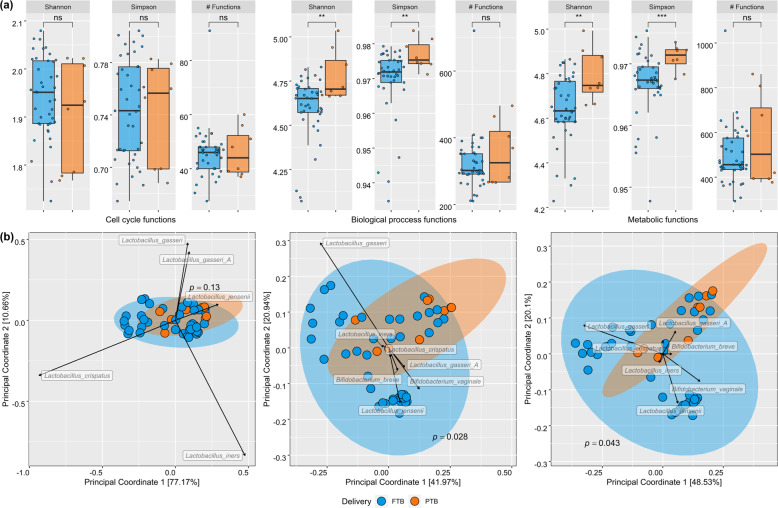
Functional diversity of the vaginal microbiome. Gene functions are divided into three higher-level functional categories based on Gene Ontology classification with both α- (**a**) and β-diversity (**b**) measures of identified content shown. Significant differences are highlighted by an asterisk where *p*-value was <0.05 as calculated by Kruskal–Wallis. Significant differences in β-diversity were determined by PERMANOVA analysis. Ellipses are generated using stat_ellipse function in R. The top seven species across all samples are labelled to highlight drivers of variation for clusters with black arrows to indicate the directionality.

**Fig. 5:**
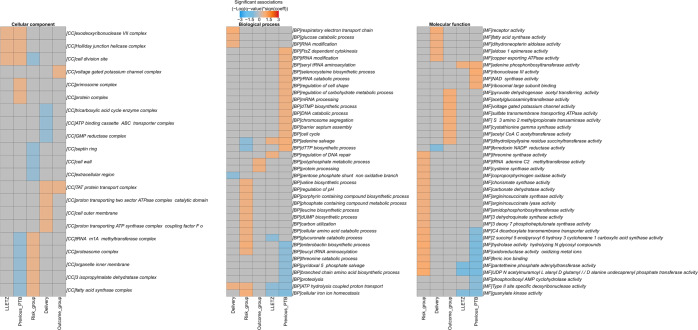
Multivariate association analysis. Each heatmap presents the independent significant associations of species to grouping as determined by MaAsLiN2 with *q*-value < 0.25. For biological process and molecular function on the top 50 associations are presented.

A total of 2928 gene functions were identified across all samples. Stratification of the data into the three Gene Ontology classifications of cellular component (CC), biological process (BP), and molecular functions (MFs) revealed functional diversity between the risk groupings. Both Shannon and Simpson diversity indexes determined there was a significant increase in the diversity of gene functions for both BPs and metabolic functions for PTB samples compared to FTB (Fig. 4a). When samples were stratified into risk groupings, this difference in functional diversity was not observed between the risk_FTB and no-risk_FTB groups (Supplementary Fig. 1a). The FTB and PTB samples were also functionally dissimilar to each other by Bray–Curtis measure both in the BP and MF classes (Fig. 4b; *p*-value 0.038 and 0.045, respectively). From ADONIS analysis, only *L. gasseri* was significantly influencing the variaton in gene function across all three categories (*p-*value 0.017 (MF), 0.027 (CC), and 0.013 (BP)). Stratification of the samples into the risk groupings did not show any significant differences in diversity of function (Supplementary Fig. 1b). Using multivariate analysis, 22 CCs, 154 BPs, and 299 MFs were significantly correlated to either women who had a previous PTB, large loop excisions of the transformation zone (LLETZ), were categorized with a prior risk for PTB, subgrouped to the four risk categories according to this study, or ultimately delivered preterm (Fig. 5 and Supplementary Table 3; *q*-value < 0.25 after multiple correction). For gene functions with significant association to preterm delivery, the MF category contained the most differential features. Among these were genes involved in ‘2′ 3′-bisphosphoglycerate-independent phosphoglycerate mutase activity’, ‘receptor activity’, ‘aldose 1 epimerase activity’, ‘carboxyl or carbamoyltransferase activity’, and ‘copper exporting ATPase activity’ (Fig. 5 and Supplementary Table 3). The top five gene functions most associated with preterm delivery within the BP category included ‘respiratory electron transport chain’, ‘alginic acid biosynthetic process’, ‘ATP hydrolysis coupled proton transport’, ‘folic acid biosynthetic process’, and ‘glucose catabolic process’ (Fig. 5 and Supplementary Table 3).

## Discussion

In summary, using a shotgun metagenomics approach, this study has confirmed the association of a vaginal microbiome dominated by *L. crispatus* with full-term pregnancies. In addition, evidence is provided that CST-4 is more associated with the vaginal microbiome from a spontaneous PTB. This study also identified functional differences in the vaginal microbiome of women who subsequently deliver preterm.

To date, numerous microbiome sequencing studies have been conducted with the aim of understanding the vaginal microbiota and its’ association with PTB. A limiting factor in several of these studies has been the use of targeted amplicon sequencing. In particular, it has been shown that certain key vaginal taxa can be underrepresented or not detected, depending on the gene region targeted. Of note, BV has been determined as a risk factor for PTB, yet the detection and differentiation of *B. vaginale* (*G. vaginalis*) subtypes can be difficult with the potential to omit subtle nuances when associating vaginal communities with PTB.

In our study, we have used shotgun metagenomics to determine the vaginal communities in women at high risk of PTB. This approach has enabled a higher resolution of the species of bacteria associated with high-risk pregnancies compared to previous approaches. The relatively high microbial read depth achieved, coupled with the recently updated GTDB has revealed a richness of species that are present at low abundance (<5%; Fig. 3). With use of the GTDB classification database, several species of *B. vaginale* were detected with each determined to correlate differently with pregnancy outcome (Table 2 and Fig. 2c). Importantly, these sub-species were not the dominant *B. vaginale*, which when present (18/49 occurences) had a mean relative abundance of 14%. In contrast, *B. vaginale_*D (9/49 occurences), *B. vaginale*_E (8/49 occurences), and *B. vaginale*_F (8/49 occurences), which significantly correlated with PTB outcome, were never >0.2% relative abundance. At such low abundance, it remains to be determined whether these species can exert a meaningful biological influence within the microbiota. Nonetheless, this difference within the *B. vaginale* group highlights the importance of stratifying this species when investigating associations with vaginal health and perinatal outcome. Another important member of BV-associated bacteria, *F. vaginae* (*A. vaginae*), was identified in three of the PTB samples at relatively high abundance (4.42–8.10%), yet was rare in full-term samples at a maximum relative abundance of 3% (in 3/41 samples). This species has been shown to develop strong biofilms with *B. vaginale*^34,35^. Previous studies identified high loads of this species as a risk for PTB^36,37^. Of interest in our study, there was an increased relative abundance of *F. vaginae* in the control group sample that had no perceived risk of PTB, yet delivered preterm (Fig. 3).

The species *Sneathia amnii* and *Prevotella amnii* have recently been identified as emerging candidates for poor pregnancy outcomes^38,39^. In our study, both of these were increased and had a significant association with PTB (*q*-value 0.02 and 0.05, respectively; Table 2). Although BVAB-1 (assigned UBA629 sp005465875 in GTDB) has been identified in microbiome studies as a potential problematic species in terms of vaginal health, this species was not identified in this study, perhaps reflective of the cohort studied as it has predominantly been reported in North American cohort studies^29,40,41^. Overall, however, the BV-associated CST, CST-4, was positively associated with PTB, a finding that is in agreement with previous studies in this area^21,42^.

Unlike previous reports, we did not see any significant differences in α-diversity between the samples from preterm and full-term pregnancies; however, there was a weak correlation between *L. crispatus* and full-term pregnancy, and an overall increased abundance of this species in samples from full-term pregnancies. This association is in agreement with several other studies and supports the concept that this species is a beneficial component of a healthy vaginal microbiome. Taken together, these studies may suggest a role of *L. crispatus* in providing protection from PTB, particularly in a Caucasian population. However, a mechanistic basis for such a role has yet to be revealed. Nonetheless, *L. crispatus* has previously been identified as a species that dominates a healthy vaginal tract, specifically in subjects free from BV^43^. It has also been linked to the stability of the vaginal microbiota during pregnancy^44^. Given that inflammation of the uterus has been linked with PTB^45^, there may be merit to assessing the ability of *L. crispatus* to provide a protective effect by suppressing inflammation through H_2_O_2_ signalling to control nuclear factor-κB activity^46^ or, indirectly, whereby the inhibition of BV-associated bacteria prevents the occurrence of a proinflammatory response to the vaginosis^47^. Importantly, a strong correlation with proinflammatory cytokines and BV-associated bacteria was observed by Fettweis et al.^39^ in a preterm cohort, further highlighting a potential microbial inflammatory mechanism for preterm onset. In general, lactobacilli have been regarded as a marker of a healthy vaginal microbiome and have been seen to improve pregnancy outcome even in the presence of risk associated taxa^37^. An exception to this is *L. iners*, which has previously been shown to associate with PTB in a similar cohort^48^. This observation was not repeated in the study.

Although these findings are important indications that merit repeated investigation, there are limitations to this dataset. In particular, due to the relatively low ratio of PTB samples, a much larger sample size will be necessary to investigate the link between the CSTs, species, and preterm outcome. Moreover, this study’s findings are specific to a Caucasian population, whilst ethnicity has been previously shown as major confounder in describing the vaginal microbiome. Notably, Romero et al.^20^ found that the composition of the vaginal microbiota did not differ between PTB and FTB across a cohort of predominately African American women. The difference in ethnicity in terms of vaginal microbiota and PTB has been further evidenced recently in a study that indicated that the frequency of *Lactobacillus*, *Gardnerella*, and *Ureaplasma* varies significantly between preterm and full-term deliveries within a Caucasian cohort but not within and African American cohort^49^. Unfortunately, socioeconomic data was not collected as part of this work, and as has been previously reported^50,51^ could be a confounder in the different microbiome profile observed. In addition, there were women in both the risk_FTB and risk_PTB groups that had received antibiotic treatment in the 6 months prior to sample collection, yet due to the low numbers is was not possible to determine the influence of this.

Although there is growing evidence for a microbiota-related role in spontaneous PTB, and indeed numerous descriptions of the importance for the microbiota with general vaginal health, there has been a limited understanding of the functional capacity of these microbial communities. As suggested by Heintz-Buschart and Wilmes^52^, a functional insight is required to develop and test hypothesis for mechanisms of action for host microbe interactions. Indeed, the sensitivity of community analysis using functional metagenomics has already been shown to distinguish healthy populations from cases of inflammatory bowel disease^53,54^, type 2 diabetes^55^, and obesity^56^. Within our preterm cohort, there was a clear distinction with respect to the functional profile of genes involved in both metabolic functions and BPs. The PTB samples had an increased abundance of functions relating to methionine/homocysteine and folate metabolism in addition to purine metabolism. Notably, maternal folate and homocysteine levels have been associated with poor pregnancy outcomes including PTB^57^. Moreover, there has been reductions in the concentrations of cysteine as measured in cervico-vaginal fluid from preterm samples in a previous study^58^. Taken together, it would suggest that further research into methionine/folate/cysteine homeostasis is merited in terms of a role for microbial metabolism and host interaction.

Overall, this study demonstrates the benefits of using shotgun metagenomics to understand the vaginal microbiome of women at risk of PTB. Moreover, we have shown that use of a microbial DNA enrichment kit is a feasible method to increase high-quality microbial sequencing reads to the upper limit of what has been achieved in previous studies (Supplementary Table 4) and overcome the notable problem of host contamination^31,59^. A distinct variation in both the taxonomic and functional potential of the preterm linked vaginal microbiome reinforces the concept of a microbiome role in PTB. However, the inability of both this study and others to determine a strict signature highlights the need for much larger controlled studies with the ability to examine confounding factors like ethnicity, biogeography, and predetermined risk factors using high-resolution, strain level analysis. This study provides evidence for the functional role of the microbiota in spontaneous PTB. Direct gene expression studies are now required if we are to fully elucidate any microbial–host interactions, which are involved in the spontaneous onset of PTB.

## Methods

### Study participants and sampling

This was a prospective cohort study with institutional ethics approval by the National Maternity Hospital Research Ethics Committee and maternal written consent. Women were determined to be at risk of spontaneous PTB if they had either a history of previous spontaneous PTB, and therefore at high risk of subsequent spontaneous PTB (*n* = 29), or had two previous LLETZ (*n* = 11). High-risk participants in the study were recruited from women attending the PTB clinic at The National Maternity Hospital Dublin, Ireland. Anonymized patient data were collected from patient charts by an independent researcher and pregnancy outcome data collected from patient charts and a computerized database in the National Maternity Hospital, Dublin. Low-risk controls (*n* = 14) were women who had previously delivered a full-term baby with no prior history of PTB or LLETZ, and were attending routine antenatal care. Inclusion criteria were women over 18 years of age and pregnant. Exclusion criteria were women currently on antibiotic treatment. Following informed consent, a high vaginal swab was taken using a speculum from the posterior vaginal fornix and external orifice of the cervix prior to transvaginal ultrasonography using a dry cotton swab and delivered to the laboratory within 24 h.

### DNA extraction/purification

One millilitre of sterile phosphate-buffered saline was added to each swab followed by rigorous vortexing for 1 min. Total microbial DNA was extracted using the MoBio PowerFood Microbial DNA isolation kit and manufacturer’s instructions. Briefly, 700 μl of swab material was centrifuged and the resulting pellet was disrupted by mechanical and enzymatic treatment. DNA was eluted at 100 μl in elution buffer and samples were stored at −20°C. DNA concentrations were determined using the Qubit high sensitivity kit as per manufacturer’s instructions. To reduce sequencing reads mapping to eukaryotic DNA downstream, DNA from each swab was treated with the Microbiome Enrichment kit (NEB) and purified with AMPure magnetic beads (Beckman Coulter). A negative sterile water control was included during the enrichment and carried forward through subsequent sequencing steps. Finally, all samples were normalized to 0.2 ng µl^−1^.

### Shotgun metagenomic sequencing

Libraries of DNA were prepared according to standard Illumina protocols. Briefly, DNA was sheared by heating to 55 °C for 7 min. Paired-end indexes were added and amplification occurred for 12 cycles before samples were purified with AMPure magnetic beads. DNA was quantified by Qubit dsDNA HS assay kit and the Agilent Bioanalyser 2100 with high sensitivity DNA chips before being pooled to 2 mM. Quality of the sample pool was confirmed by quantitative PCR. A sample of sterile water was processed in parallel with the DNA during library preparation to act as a negative control (Supplementary Table 5). Libraries were sequenced using 2 × 150 bp paired-end kit on the Illumina NextSeq platform.

### Bioinformatic analysis

Raw sequencing data were base-called using Illumina’s bcl2fastq software (v 2.19) (https://support.illumina.com/sequencing/sequencing_software/bcl2fastq-conversion-software.html). TrimGalore (v 0.6.0) (http://www.bioinformatics.babraham.ac.uk/projects/trim_galore/), a perl wrapper for Cutadapt (v. 1.18)^60^ and FastQC (v. 0.11.8) (http://www.bioinformatics.babraham.ac.uk/projects/fastqc/), was used to remove adapter sequences and low-quality sequences using default parameters. Removal of human DNA contamination was performed by aligning all high-quality paired-end reads to the latest draft of the human genome (hg38) using Bowtie2 (v. 2.3.4)^61^. The resulting SAM files were converted to BAM format and filtered to keep only unmapped paired-end reads using SAMtools^62^. Bedtools^63^ was used to convert the remaining reads from BAM to FASTQ format. Taxonomic assignment of paired-end reads was performed using Kraken2^64^ alignment against the GTDB_54k database created by Méric et al.^65^ (https://github.com/rrwick/Metagenomics-Index-Correction). Functional profiling was performed using the HUMAnN2 pipeline (v. 2.8.1)^66^. The gene families output were renormalized as copies per million reads and regrouped according to Gene Ontology terms.

Data were visualized using both Graphpad Prism 6 and RStudio (R v.3.6.0). Heatmaps were generated using the ‘ComplexHeatmap’ package^67^ with samples clustered using the Pearson’s distance metric and columns split by *k*-means clustering to visualize CSTs, assigned based on previous reported definitions^24^. Plots for diversity analysis were generated using the ggplot2 package (https://cran.r-project.org/web/packages/ggplot2/index.html). Statistical analysis was carried out in R using the vegan package (https://cran.r-project.org/web/packages/vegan/index.html) and RVAideMemoire (https://cran.r-project.org/web/packages/RVAideMemoire/index.html). Multivariate Association with Linear Models 2 (MaAsLiN2, R V.1.2.0) was used to determine independent associations of species and functions with metadata factors. A *q*-value of >0.25 was considered significant in our analysis.

### Reporting summary

Further information on research design is available in the Nature Research Reporting Summary linked to this article.

## Supplementary information

Supplementary Information

Reporting Summary

## Data Availability

Sequence data have been deposited in the European Nucleotide Archive (ENA) under the study accession number PRJEB34536.
