# Supplementary material for: Shotgun sequencing of the vaginal microbiome reveals both a species and functional potential signature of preterm birth
Source: NPJ Biofilms Microbiomes. 2020 Nov 12;6:50. doi: 10.1038/s41522-020-00162-8 (PMC7665020; doi:10.1038/s41522-020-00162-8)
Supplement: Supplementary file 1 — Supplementary Information [file 41522_2020_162_MOESM1_ESM.pdf]

Supplementary Table 1 | Determination of associations between risk groups and microbiota

	no-risk_FTB	risk_FTB	risk_PTB	FTB/PTB
CST-1	0.08	0.76	0.08	0.09
CST-2	0.42	0.23	0.32	1
CST-3	1	1	1	1
CST-4	0.31	0.64	0.02*	0.02*
CST-5	1	0.30	0.09	0.12
CST-8	1	0.62	1	1

*Asterisk highlights significant associations as determined by Fishers exact test using fisher.test function in R

Supplementary Table 2 | Full MaAsLiN2 output for significant associations of species and sample groupings

**metadata**	**feature**	**value**	**coef**	**stderr**	**N**	**N.not.0**	**pval**	**qval**
Outcome_group	Alloscardovia.omnicolens	no-risk_PTB	0.569544	0.049605	49	0	1.11E-14	5.33E-11
Outcome_group	Clostridium_K.cellulovorans	no-risk_PTB	0.37881	0.050281	49	0	2.19E-09	5.27E-06
LLETZ	Paracoccus.sp000967825	Yes	0.399805	0.065555	49	0	2.63E-07	0.000422
Previous_PTB	Paracoccus.sp000967825	Yes	0.381937	0.068191	49	0	1.39E-06	0.00067
Risk_group	Paracoccus.sp000967825	Risk	-0.36568	0.073786	49	0	1.17E-05	0.00067
LLETZ	X2.01.FULL.66.17.sp001803875	Yes	0.106814	0.022004	49	0	1.63E-05	0.00067
LLETZ	Achromobacter.sp001299435	Yes	0.106814	0.022004	49	0	1.63E-05	0.00067
LLETZ	Agrobacterium.sp900013535	Yes	0.106814	0.022004	49	0	1.63E-05	0.00067
LLETZ	Akkermansia.glycaniphila	Yes	0.106814	0.022004	49	0	1.63E-05	0.00067
LLETZ	Aliarcobacter.cryaerophilus_A	Yes	0.106814	0.022004	49	0	1.63E-05	0.00067

LLETZ	Alistipes.onderdonkii	Yes	0.106814	0.022004	49	0	1.63E-05	0.00067
LLETZ	Alistipes.senegalensis	Yes	0.106814	0.022004	49	0	1.63E-05	0.00067
LLETZ	Alistipes_A.indistinctus	Yes	0.106814	0.022004	49	0	1.63E-05	0.00067
LLETZ	ASF356.sp000364165	Yes	0.106814	0.022004	49	0	1.63E-05	0.00067
LLETZ	Bact.08.sp000473305	Yes	0.106814	0.022004	49	0	1.63E-05	0.00067
LLETZ	Bacteroides_A.plebeius	Yes	0.106814	0.022004	49	0	1.63E-05	0.00067
LLETZ	Baumannia.cicadellinicola_C	Yes	0.106814	0.022004	49	0	1.63E-05	0.00067
Previous_PTB	Bifidobacterium.callitrichos	Yes	-0.11309	0.022683	49	0	1.06E-05	0.00067
Risk_group	Bifidobacterium.callitrichos	Risk	0.119702	0.024544	49	0	1.51E-05	0.00067
Previous_PTB	Bifidobacterium.gallinarum	Yes	-0.11309	0.022683	49	0	1.06E-05	0.00067
Risk_group	Bifidobacterium.gallinarum	Risk	0.119702	0.024544	49	0	1.51E-05	0.00067
Previous_PTB	Bifidobacterium.sp002451435	Yes	-0.11309	0.022683	49	0	1.06E-05	0.00067

Risk_group	Bifidobacterium.sp002451435	Risk	0.119702	0.024544	49	0	1.51E-05	0.00067
LLETZ	Blastococcus.sp003075095	Yes	0.106814	0.022004	49	0	1.63E-05	0.00067
LLETZ	Blautia_A.sp000285855	Yes	0.106814	0.022004	49	0	1.63E-05	0.00067
LLETZ	Brevundimonas.sp002157625	Yes	0.106814	0.022004	49	0	1.63E-05	0.00067
LLETZ	Brevundimonas.sp002484265	Yes	0.106814	0.022004	49	0	1.63E-05	0.00067
LLETZ	Butyricicoccus.pullicaecorum	Yes	0.106814	0.022004	49	0	1.63E-05	0.00067
LLETZ	Butyricimonas.sp002161485	Yes	0.106814	0.022004	49	0	1.63E-05	0.00067
LLETZ	Butyrivibrio_A.crossotus	Yes	0.106814	0.022004	49	0	1.63E-05	0.00067
LLETZ	CAG.110.sp002437585	Yes	0.106814	0.022004	49	0	1.63E-05	0.00067
LLETZ	CAG.194.sp000432915	Yes	0.106814	0.022004	49	0	1.63E-05	0.00067
LLETZ	CAG.302.sp002375205	Yes	0.106814	0.022004	49	0	1.63E-05	0.00067
Previous_PTB	CAG.590.sp003530125	Yes	-0.16936	0.034292	49	0	1.24E-05	0.00067
LLETZ	CAG.83.sp000435555	Yes	0.106814	0.022004	49	0	1.63E-05	0.00067

LLETZ	CAG.83.sp001916855	Yes	0.106814	0.022004	49	0	1.63E-05	0.00067
LLETZ	Campylobacter_D.jejuni	Yes	0.106814	0.022004	49	0	1.63E-05	0.00067
LLETZ	Clostridium.taeniosporum	Yes	0.106814	0.022004	49	0	1.63E-05	0.00067
LLETZ	Clostridium_AN.bornimense	Yes	0.106814	0.022004	49	0	1.63E-05	0.00067
Previous_PTB	Clostridium_M.clostridioforme	Yes	-0.11309	0.022683	49	0	1.06E-05	0.00067
Risk_group	Clostridium_M.clostridioforme	Risk	0.119702	0.024544	49	0	1.51E-05	0.00067
LLETZ	Clostridium_Q.sp003024715	Yes	0.106814	0.022004	49	0	1.63E-05	0.00067
LLETZ	Corynebacterium.appendicis	Yes	0.106814	0.022004	49	0	1.63E-05	0.00067
Previous_PTB	Corynebacterium.coyleae	Yes	-0.11309	0.022683	49	0	1.06E-05	0.00067
Risk_group	Corynebacterium.coyleae	Risk	0.119702	0.024544	49	0	1.51E-05	0.00067
LLETZ	Corynebacterium.kefirresidentii	Yes	0.106814	0.022004	49	0	1.63E-05	0.00067
LLETZ	Corynebacterium.otitidis	Yes	0.106814	0.022004	49	0	1.63E-05	0.00067
LLETZ	Cupriavidus.gilardii	Yes	0.106	0.022	49	0	1.63E	0.000

			814	004			-05	67
LLETZ	Dorea.formicigenerans	Yes	0.106814	0.022004	49	0	1.63E-05	0.00067
LLETZ	Dorea.sp900066555	Yes	0.106814	0.022004	49	0	1.63E-05	0.00067
LLETZ	DTU078.sp002385415	Yes	0.106814	0.022004	49	0	1.63E-05	0.00067
LLETZ	Erysipelatoclostridium.spiroforme	Yes	0.106814	0.022004	49	0	1.63E-05	0.00067
LLETZ	Eubacterium_E.hallii_A	Yes	0.106814	0.022004	49	0	1.63E-05	0.00067
LLETZ	Eubacterium_H.cellulosolvens	Yes	0.106814	0.022004	49	0	1.63E-05	0.00067
LLETZ	Eubacterium_S.uniforme	Yes	0.106814	0.022004	49	0	1.63E-05	0.00067
LLETZ	Faecalibacterium.prausnitzii_C	Yes	0.106814	0.022004	49	0	1.63E-05	0.00067
LLETZ	Faecalibacterium.prausnitzii_D	Yes	0.106814	0.022004	49	0	1.63E-05	0.00067
Previous_PTB	Faecalicatena.glycyrrhizinilyticum	Yes	-0.11309	0.022683	49	0	1.06E-05	0.00067
Risk_group	Faecalicatena.glycyrrhizinilyticum	Risk	0.119702	0.024544	49	0	1.51E-05	0.00067
Previous_PTB	Faecalicatena.sp000403295	Yes	-0.11309	0.022683	49	0	1.06E-05	0.00067

Risk_group	Faecalicatena.sp000403295	Risk	0.119702	0.024544	49	0	1.51E-05	0.00067
LLETZ	Fastidiosipila.sanguinis	Yes	0.106814	0.022004	49	0	1.63E-05	0.00067
LLETZ	Flavonifractor.sp002161085	Yes	0.106814	0.022004	49	0	1.63E-05	0.00067
LLETZ	GCA.900066575.sp900066385	Yes	0.106814	0.022004	49	0	1.63E-05	0.00067
LLETZ	Gordonia.jacobaea	Yes	0.106814	0.022004	49	0	1.63E-05	0.00067
LLETZ	GWC2.39.14.sp000996245	Yes	0.106814	0.022004	49	0	1.63E-05	0.00067
LLETZ	Hungatella.effluvii	Yes	0.106814	0.022004	49	0	1.63E-05	0.00067
LLETZ	Ileibacterium.valens	Yes	0.106814	0.022004	49	0	1.63E-05	0.00067
LLETZ	KLE1615.sp900066985	Yes	0.106814	0.022004	49	0	1.63E-05	0.00067
Previous_PTB	Lachnospira.eligens_B	Yes	-0.11309	0.022683	49	0	1.06E-05	0.00067
Risk_group	Lachnospira.eligens_B	Risk	0.119702	0.024544	49	0	1.51E-05	0.00067
Outcome_group	Lactobacillus.intestinalis	no-risk_PTB	1.734946	0.336891	49	0	6.19E-06	0.00067
LLETZ	Lysinibacillus.fusiformi	Yes	0.106	0.022	49	0	1.63E	0.000

	s_D		814	004			-05	67
LLETZ	Marvinbryantia.sp900066075	Yes	0.106814	0.022004	49	0	1.63E-05	0.00067
LLETZ	Methylobacterium.organophilum	Yes	0.106814	0.022004	49	0	1.63E-05	0.00067
LLETZ	Micromonospora.sp003176735	Yes	0.106814	0.022004	49	0	1.63E-05	0.00067
LLETZ	Negativibacillus.massiliensis	Yes	0.106814	0.022004	49	0	1.63E-05	0.00067
LLETZ	OEMS01.sp900199405	Yes	0.106814	0.022004	49	0	1.63E-05	0.00067
LLETZ	Oxalobacter.formigenes_B	Yes	0.106814	0.022004	49	0	1.63E-05	0.00067
LLETZ	Paludibacter_A.jiangxiensis	Yes	0.106814	0.022004	49	0	1.63E-05	0.00067
LLETZ	Parabacteroides.timonensis	Yes	0.106814	0.022004	49	0	1.63E-05	0.00067
LLETZ	Parasporobacterium.paucivorans	Yes	0.106814	0.022004	49	0	1.63E-05	0.00067
LLETZ	PeH17.sp000435055	Yes	0.430891	0.081758	49	0	4.16E-06	0.00067
Previous_PTB	PeH17.sp000435055	Yes	0.4132	0.085046	49	0	1.61E-05	0.00067
Delivery	Prevotella.bivia	PTB	0.776951	0.153928	49	0	8.66E-06	0.00067
LLETZ	Prevotella.sp002251365	Yes	0.106814	0.022004	49	0	1.63E-05	0.00067

LLETZ	Pseudomonas_E.hunanensis	Yes	0.106814	0.022004	49	0	1.63E-05	0.00067
LLETZ	QAMH01.sp003149935	Yes	0.219881	0.038891	49	0	1.16E-06	0.00067
Previous_PTB	QAMH01.sp003149935	Yes	0.222808	0.040455	49	0	1.89E-06	0.00067
LLETZ	Ralstonia.pickettii	Yes	0.106814	0.022004	49	0	1.63E-05	0.00067
LLETZ	Ralstonia.pseudosolanacearum	Yes	0.106814	0.022004	49	0	1.63E-05	0.00067
LLETZ	RC9.sp000434935	Yes	0.106814	0.022004	49	0	1.63E-05	0.00067
LLETZ	Rubellimicrobium.thermophilum	Yes	0.106814	0.022004	49	0	1.63E-05	0.00067
LLETZ	RUG210.sp003265305	Yes	0.106814	0.022004	49	0	1.63E-05	0.00067
LLETZ	RUG350.sp900315995	Yes	0.106814	0.022004	49	0	1.63E-05	0.00067
LLETZ	Ruminococcus_F.champanellensis	Yes	0.106814	0.022004	49	0	1.63E-05	0.00067
LLETZ	Soleaferrea.massiliensis	Yes	0.106814	0.022004	49	0	1.63E-05	0.00067
LLETZ	Sporobacter.termitidis	Yes	0.106814	0.022004	49	0	1.63E-05	0.00067
LLETZ	Stenotrophomonas.maltophilia	Yes	0.106814	0.022004	49	0	1.63E-05	0.00067

LLETZ	Stenotrophomonas.rhizophila_A	Yes	0.106814	0.022004	49	0	1.63E-05	0.00067
LLETZ	Streptococcus.thermophilus	Yes	0.106814	0.022004	49	0	1.63E-05	0.00067
LLETZ	Streptomyces.papulosa	Yes	0.106814	0.022004	49	0	1.63E-05	0.00067
LLETZ	Sutterella.wadsworthensis_B	Yes	0.106814	0.022004	49	0	1.63E-05	0.00067
LLETZ	SZUA.51.sp003232965	Yes	0.106814	0.022004	49	0	1.63E-05	0.00067
LLETZ	Tepidanaerobacter.syntrophicus	Yes	0.106814	0.022004	49	0	1.63E-05	0.00067
LLETZ	TF01.11.sp003524945	Yes	0.106814	0.022004	49	0	1.63E-05	0.00067
LLETZ	Tidjanibacter.inops	Yes	0.106814	0.022004	49	0	1.63E-05	0.00067
Previous_PTB	Tyzzerella.nexilis	Yes	-0.11309	0.022683	49	0	1.06E-05	0.00067
Risk_group	Tyzzerella.nexilis	Risk	0.119702	0.024544	49	0	1.51E-05	0.00067
LLETZ	UBA11774.sp003507655	Yes	0.106814	0.022004	49	0	1.63E-05	0.00067
LLETZ	UBA1191.sp900066305	Yes	0.106814	0.022004	49	0	1.63E-05	0.00067
LLETZ	UBA1259.sp900320065	Yes	0.106814	0.022004	49	0	1.63E-05	0.00067

LLETZ	UBA3566.sp002376065	Yes	0.106814	0.022004	49	0	1.63E-05	0.00067
LLETZ	UBA7524.sp002478275	Yes	0.106814	0.022004	49	0	1.63E-05	0.00067
LLETZ	UBA9655.sp001767835	Yes	0.106814	0.022004	49	0	1.63E-05	0.00067
LLETZ	Veillonella.rogosae	Yes	0.106814	0.022004	49	0	1.63E-05	0.00067
LLETZ	W11650.sp002999035	Yes	0.106814	0.022004	49	0	1.63E-05	0.00067
LLETZ	Mycolicibacterium.sp001665685	Yes	0.602987	0.127271	49	0	2.38E-05	0.00097
Previous_PTB	X2.01.FULL.66.17.sp001803875	Yes	0.10318	0.022889	49	0	4.98E-05	0.001105
Previous_PTB	Achromobacter.sp001299435	Yes	0.10318	0.022889	49	0	4.98E-05	0.001105
Previous_PTB	Agrobacterium.sp900013535	Yes	0.10318	0.022889	49	0	4.98E-05	0.001105
Previous_PTB	Akkermansia.glycaniphila	Yes	0.10318	0.022889	49	0	4.98E-05	0.001105
Previous_PTB	Aliarcobacter.cryaerophilus_A	Yes	0.10318	0.022889	49	0	4.98E-05	0.001105
Previous_PTB	Alistipes.onderdonkii	Yes	0.10318	0.022889	49	0	4.98E-05	0.001105
LLETZ	Alistipes.putredinis	Yes	0.361541	0.079416	49	0	4.32E-05	0.001105

Previous_ PTB	Alistipes.senegalensis	Yes	0.103 18	0.022 889	49	0	4.98E -05	0.001 105
Previous_ PTB	Alistipes_A.indistinctus	Yes	0.103 18	0.022 889	49	0	4.98E -05	0.001 105
Previous_ PTB	ASF356.sp000364165	Yes	0.103 18	0.022 889	49	0	4.98E -05	0.001 105
Previous_ PTB	Bact.08.sp000473305	Yes	0.103 18	0.022 889	49	0	4.98E -05	0.001 105
Previous_ PTB	Bacteroides_A.plebeius	Yes	0.103 18	0.022 889	49	0	4.98E -05	0.001 105
Previous_ PTB	Baumannia.cicadellinic ola_C	Yes	0.103 18	0.022 889	49	0	4.98E -05	0.001 105
LLETZ	Bifidobacterium.callitric hos	Yes	- 0.100 21	0.021 806	49	0	3.76E -05	0.001 105
LLETZ	Bifidobacterium.gallinar um	Yes	- 0.100 21	0.021 806	49	0	3.76E -05	0.001 105
LLETZ	Bifidobacterium.sp0024 51435	Yes	- 0.100 21	0.021 806	49	0	3.76E -05	0.001 105
Previous_ PTB	Blastococcus.sp003075 095	Yes	0.103 18	0.022 889	49	0	4.98E -05	0.001 105
Previous_ PTB	Blautia_A.sp000285855	Yes	0.103 18	0.022 889	49	0	4.98E -05	0.001 105
Previous_ PTB	Brevundimonas.sp0021 57625	Yes	0.103 18	0.022 889	49	0	4.98E -05	0.001 105

Previous_PTB	Brevundimonas.sp002484265	Yes	0.10318	0.022889	49	0	4.98E-05	0.001105
Previous_PTB	Butyricicoccus.pullicaecorum	Yes	0.10318	0.022889	49	0	4.98E-05	0.001105
Previous_PTB	Butyricimonas.sp002161485	Yes	0.10318	0.022889	49	0	4.98E-05	0.001105
Previous_PTB	Butyrivibrio_A.crossotus	Yes	0.10318	0.022889	49	0	4.98E-05	0.001105
Previous_PTB	CAG.110.sp002437585	Yes	0.10318	0.022889	49	0	4.98E-05	0.001105
Previous_PTB	CAG.194.sp000432915	Yes	0.10318	0.022889	49	0	4.98E-05	0.001105
Previous_PTB	CAG.302.sp002375205	Yes	0.10318	0.022889	49	0	4.98E-05	0.001105
Previous_PTB	CAG.83.sp000435555	Yes	0.10318	0.022889	49	0	4.98E-05	0.001105
Previous_PTB	CAG.83.sp001916855	Yes	0.10318	0.022889	49	0	4.98E-05	0.001105
Previous_PTB	Campylobacter_D.jejuni	Yes	0.10318	0.022889	49	0	4.98E-05	0.001105
Previous_PTB	Clostridium.taeniosporum	Yes	0.10318	0.022889	49	0	4.98E-05	0.001105
Previous_PTB	Clostridium_AN.bornimense	Yes	0.10318	0.022889	49	0	4.98E-05	0.001105
LLETZ	Clostridium_M.clostridioforme	Yes	-0.10021	0.021806	49	0	3.76E-05	0.001105

Previous_PTB	Clostridium_Q.sp003024715	Yes	0.10318	0.022889	49	0	4.98E-05	0.001105
Previous_PTB	Corynebacterium.appendicis	Yes	0.10318	0.022889	49	0	4.98E-05	0.001105
LLETZ	Corynebacterium.coyleae	Yes	-0.10021	0.021806	49	0	3.76E-05	0.001105
Previous_PTB	Corynebacterium.kefirresidentii	Yes	0.10318	0.022889	49	0	4.98E-05	0.001105
Previous_PTB	Corynebacterium.otitidis	Yes	0.10318	0.022889	49	0	4.98E-05	0.001105
Previous_PTB	Cupriavidus.gilardii	Yes	0.10318	0.022889	49	0	4.98E-05	0.001105
Delivery	Dialister.micraerophilus	PTB	0.573332	0.123143	49	0	3.10E-05	0.001105
Previous_PTB	Dorea.formicigenerans	Yes	0.10318	0.022889	49	0	4.98E-05	0.001105
Previous_PTB	Dorea.sp900066555	Yes	0.10318	0.022889	49	0	4.98E-05	0.001105
Previous_PTB	DTU078.sp002385415	Yes	0.10318	0.022889	49	0	4.98E-05	0.001105
Previous_PTB	Erysipelatoclostridium.spiroforme	Yes	0.10318	0.022889	49	0	4.98E-05	0.001105
Previous_PTB	Eubacterium_E.hallii_A	Yes	0.10318	0.022889	49	0	4.98E-05	0.001105
Previous_PTB	Eubacterium_H.cellulosolvens	Yes	0.10318	0.022889	49	0	4.98E-05	0.001105

Previous_PTB	Eubacterium_S.uniforme	Yes	0.10318	0.022889	49	0	4.98E-05	0.001105
Previous_PTB	Faecalibacterium.prausnitzii_C	Yes	0.10318	0.022889	49	0	4.98E-05	0.001105
Previous_PTB	Faecalibacterium.prausnitzii_D	Yes	0.10318	0.022889	49	0	4.98E-05	0.001105
LLETZ	Faecalicatena.glycyrrhizinilyticum	Yes	-0.10021	0.021806	49	0	3.76E-05	0.001105
LLETZ	Faecalicatena.sp000403295	Yes	-0.10021	0.021806	49	0	3.76E-05	0.001105
Previous_PTB	Fastidiosipila.sanguinis	Yes	0.10318	0.022889	49	0	4.98E-05	0.001105
Previous_PTB	Flavonifractor.sp002161085	Yes	0.10318	0.022889	49	0	4.98E-05	0.001105
Previous_PTB	GCA.900066575.sp900066385	Yes	0.10318	0.022889	49	0	4.98E-05	0.001105
Previous_PTB	Gordonia.jacobaea	Yes	0.10318	0.022889	49	0	4.98E-05	0.001105
Previous_PTB	GWC2.39.14.sp000996245	Yes	0.10318	0.022889	49	0	4.98E-05	0.001105
Previous_PTB	Hungatella.effluvii	Yes	0.10318	0.022889	49	0	4.98E-05	0.001105
Previous_PTB	Ileibacterium.valens	Yes	0.10318	0.022889	49	0	4.98E-05	0.001105
Previous_	KLE1615.sp900066985	Yes	0.103	0.022	49	0	4.98E	0.001

PTB			18	889			-05	105
LLETZ	Lachnospira.eligens_B	Yes	-0.10021	0.021806	49	0	3.76E-05	0.001105
Previous_PTB	Lysinibacillus.fusiformis_D	Yes	0.10318	0.022889	49	0	4.98E-05	0.001105
Previous_PTB	Marvinbryantia.sp900066075	Yes	0.10318	0.022889	49	0	4.98E-05	0.001105
Previous_PTB	Methylobacterium.organophilum	Yes	0.10318	0.022889	49	0	4.98E-05	0.001105
Previous_PTB	Micromonospora.sp003176735	Yes	0.10318	0.022889	49	0	4.98E-05	0.001105
Previous_PTB	Mycolicibacterium.sp001665685	Yes	0.597135	0.132389	49	0	4.94E-05	0.001105
Previous_PTB	Negativibacillus.massiliensis	Yes	0.10318	0.022889	49	0	4.98E-05	0.001105
Previous_PTB	OEMS01.sp900199405	Yes	0.10318	0.022889	49	0	4.98E-05	0.001105
Previous_PTB	Oxalobacter.formigenes_B	Yes	0.10318	0.022889	49	0	4.98E-05	0.001105
Previous_PTB	Paludibacter_A.jiangxiensis	Yes	0.10318	0.022889	49	0	4.98E-05	0.001105
Previous_PTB	Parabacteroides.timonensis	Yes	0.10318	0.022889	49	0	4.98E-05	0.001105
Previous_PTB	Parasporobacterium.paucivorans	Yes	0.10318	0.022889	49	0	4.98E-05	0.001105
Previous_	Prevotella.sp002251365	Yes	0.103	0.022	49	0	4.98E	0.001

PTB			18	889				-05	105
Previous_ PTB	Pseudomonas_E.hunane nsis	Yes	0.103 18	0.022 889	49	0		4.98E -05	0.001 105
Previous_ PTB	Ralstonia.pickettii	Yes	0.103 18	0.022 889	49	0		4.98E -05	0.001 105
Previous_ PTB	Ralstonia.pseudosolana cearum	Yes	0.103 18	0.022 889	49	0		4.98E -05	0.001 105
Previous_ PTB	RC9.sp000434935	Yes	0.103 18	0.022 889	49	0		4.98E -05	0.001 105
Previous_ PTB	Rubellimicrobium.ther mophilum	Yes	0.103 18	0.022 889	49	0		4.98E -05	0.001 105
Previous_ PTB	RUG210.sp003265305	Yes	0.103 18	0.022 889	49	0		4.98E -05	0.001 105
Previous_ PTB	RUG350.sp900315995	Yes	0.103 18	0.022 889	49	0		4.98E -05	0.001 105
Previous_ PTB	Ruminococcus_F.cham panellensis	Yes	0.103 18	0.022 889	49	0		4.98E -05	0.001 105
Previous_ PTB	Soleaferrea.massiliensis	Yes	0.103 18	0.022 889	49	0		4.98E -05	0.001 105
Previous_ PTB	Sporobacter.termitidis	Yes	0.103 18	0.022 889	49	0		4.98E -05	0.001 105
Previous_ PTB	Stenotrophomonas.malt ophilia	Yes	0.103 18	0.022 889	49	0		4.98E -05	0.001 105
Previous_ PTB	Stenotrophomonas.rhizo phila_A	Yes	0.103 18	0.022 889	49	0		4.98E -05	0.001 105
Previous_ PTB	Streptococcus.thermoph ilus	Yes	0.103 18	0.022 889	49	0		4.98E -05	0.001 105

Previous_PTB	Streptomyces.papulosa	Yes	0.10318	0.022889	49	0	4.98E-05	0.001105
Previous_PTB	Sutterella.wadsworthensis_B	Yes	0.10318	0.022889	49	0	4.98E-05	0.001105
Previous_PTB	SZUA.51.sp003232965	Yes	0.10318	0.022889	49	0	4.98E-05	0.001105
Previous_PTB	Tepidanaerobacter.syntrophicus	Yes	0.10318	0.022889	49	0	4.98E-05	0.001105
Previous_PTB	TF01.11.sp003524945	Yes	0.10318	0.022889	49	0	4.98E-05	0.001105
Previous_PTB	Tidjanibacter.inops	Yes	0.10318	0.022889	49	0	4.98E-05	0.001105
LLETZ	Tyzzerella.nexilis	Yes	-0.10021	0.021806	49	0	3.76E-05	0.001105
Previous_PTB	UBA11774.sp003507655	Yes	0.10318	0.022889	49	0	4.98E-05	0.001105
Previous_PTB	UBA1191.sp900066305	Yes	0.10318	0.022889	49	0	4.98E-05	0.001105
Previous_PTB	UBA1259.sp900320065	Yes	0.10318	0.022889	49	0	4.98E-05	0.001105
Previous_PTB	UBA3566.sp002376065	Yes	0.10318	0.022889	49	0	4.98E-05	0.001105
Previous_PTB	UBA7524.sp002478275	Yes	0.10318	0.022889	49	0	4.98E-05	0.001105
Previous_PTB	UBA9655.sp001767835	Yes	0.10318	0.022889	49	0	4.98E-05	0.001105

Previous_PTB	Veillonella.rogosae	Yes	0.10318	0.022889	49	0	4.98E-05	0.001105
Previous_PTB	W11650.sp002999035	Yes	0.10318	0.022889	49	0	4.98E-05	0.001105
LLETZ	TF01.11.sp001414325	Yes	0.327155	0.072806	49	0	5.21E-05	0.001152
Risk_group	QAMH01.sp003149935	Risk	-0.19646	0.043775	49	0	5.31E-05	0.001167
Risk_group	CAG.590.sp003530125	Risk	0.165181	0.037106	49	0	5.96E-05	0.001304
LLETZ	Helicobacter.pylori	Yes	0.237067	0.053404	49	0	6.20E-05	0.001351
Previous_PTB	Alistipes.putredinis	Yes	0.363906	0.082609	49	0	6.91E-05	0.001498
LLETZ	Alistipes.sp000434235	Yes	0.282823	0.064433	49	0	7.26E-05	0.00152
LLETZ	Bacteroides.helcogenes	Yes	0.282823	0.064433	49	0	7.26E-05	0.00152
LLETZ	CAG.462.sp900291465	Yes	0.282823	0.064433	49	0	7.26E-05	0.00152
LLETZ	Coprobacter.fastidiosus	Yes	0.282823	0.064433	49	0	7.26E-05	0.00152
LLETZ	Parabacteroides.sp900155425	Yes	0.282823	0.064433	49	0	7.26E-05	0.00152
LLETZ	RUG762.sp900316495	Yes	0.282823	0.064433	49	0	7.26E-05	0.00152

LLETZ	Senegalimassilia.anaerobia	Yes	0.282823	0.064433	49	0	7.26E-05	0.00152
LLETZ	Turicibacter.sp001543345	Yes	0.282823	0.064433	49	0	7.26E-05	0.00152
LLETZ	Blautia_A.sp900066145	Yes	0.343238	0.078591	49	0	7.79E-05	0.001621
Previous_PTB	TF01.11.sp001414325	Yes	0.330689	0.075734	49	0	7.81E-05	0.001621
LLETZ	GWF2.35.48.sp001769125	Yes	0.09401	0.021652	49	0	8.44E-05	0.001729
LLETZ	Methanobrevibacter.ruminantium	Yes	0.09401	0.021652	49	0	8.44E-05	0.001729
LLETZ	TMED.70.sp002171675	Yes	0.09401	0.021652	49	0	8.44E-05	0.001729
Previous_PTB	Alistipes.sp000434235	Yes	0.287866	0.067024	49	0	9.79E-05	0.001939
Previous_PTB	Bacteroides.helcogenes	Yes	0.287866	0.067024	49	0	9.79E-05	0.001939
Previous_PTB	CAG.462.sp900291465	Yes	0.287866	0.067024	49	0	9.79E-05	0.001939
Previous_PTB	Coprobacter.fastidiosus	Yes	0.287866	0.067024	49	0	9.79E-05	0.001939
Previous_PTB	Parabacteroides.sp900155425	Yes	0.287866	0.067024	49	0	9.79E-05	0.001939
Previous_PTB	RUG762.sp900316495	Yes	0.287866	0.067024	49	0	9.79E-05	0.001939

Previous_PTB	Senegalimassilia.anaerobia	Yes	0.287866	0.067024	49	0	9.79E-05	0.001939
Previous_PTB	Turicibacter.sp001543345	Yes	0.287866	0.067024	49	0	9.79E-05	0.001939
LLETZ	CAG.110.sp003525905	Yes	0.298906	0.071164	49	0	0.000132	0.002599
LLETZ	Acetatifactor.sp900066365	Yes	0.22034	0.053059	49	0	0.000153	0.002797
LLETZ	Alistipes.sp900021155	Yes	0.22034	0.053059	49	0	0.000153	0.002797
LLETZ	Bacteroides.ndongoniae	Yes	0.22034	0.053059	49	0	0.000153	0.002797
LLETZ	Bacteroides.sp002160055	Yes	0.22034	0.053059	49	0	0.000153	0.002797
Delivery	Bifidobacterium.vaginale_F	PTB	0.434851	0.104444	49	0	0.000148	0.002797
LLETZ	Blautia_A.wexlerae	Yes	0.22034	0.053059	49	0	0.000153	0.002797
LLETZ	Brevundimonas.sp002434505	Yes	0.22034	0.053059	49	0	0.000153	0.002797
LLETZ	CAG.354.sp001915925	Yes	0.22034	0.053059	49	0	0.000153	0.002797
LLETZ	CAG.56.sp900066615	Yes	0.22034	0.053059	49	0	0.000153	0.002797
LLETZ	Delftia.acidovorans	Yes	0.22034	0.053059	49	0	0.000153	0.002797

LLETZ	F0540.sp000466585	Yes	0.22034	0.053059	49	0	0.000153	0.002797
LLETZ	Faecalibacterium.prausnitzii_G	Yes	0.22034	0.053059	49	0	0.000153	0.002797
LLETZ	GCA.900066135.sp900066135	Yes	0.22034	0.053059	49	0	0.000153	0.002797
LLETZ	Gemmiger.sp003476825	Yes	0.22034	0.053059	49	0	0.000153	0.002797
LLETZ	Gemmiger.variabile	Yes	0.22034	0.053059	49	0	0.000153	0.002797
LLETZ	Parasutterella.excrementihominis	Yes	0.22034	0.053059	49	0	0.000153	0.002797
LLETZ	Roseburia.hominis	Yes	0.22034	0.053059	49	0	0.000153	0.002797
LLETZ	Ruminococcus_A.sp003011855	Yes	0.22034	0.053059	49	0	0.000153	0.002797
LLETZ	UBA11524.sp000437595	Yes	0.22034	0.053059	49	0	0.000153	0.002797
Previous_PTB	Acetatifactor.sp900066365	Yes	0.22751	0.055193	49	0	0.000168	0.00288
Previous_PTB	Alistipes.sp900021155	Yes	0.22751	0.055193	49	0	0.000168	0.00288
Previous_PTB	Bacteroides.ndongoniae	Yes	0.22751	0.055193	49	0	0.000168	0.00288
Previous_PTB	Bacteroides.sp002160055	Yes	0.22751	0.055193	49	0	0.000168	0.00288

Previous_PTB	Blautia_A.wexlerae	Yes	0.22751	0.055193	49	0	0.000168	0.00288
Previous_PTB	Brevundimonas.sp002434505	Yes	0.22751	0.055193	49	0	0.000168	0.00288
Previous_PTB	CAG.354.sp001915925	Yes	0.22751	0.055193	49	0	0.000168	0.00288
Previous_PTB	CAG.56.sp900066615	Yes	0.22751	0.055193	49	0	0.000168	0.00288
Previous_PTB	Delftia.acidovorans	Yes	0.22751	0.055193	49	0	0.000168	0.00288
Previous_PTB	F0540.sp000466585	Yes	0.22751	0.055193	49	0	0.000168	0.00288
Previous_PTB	Faecalibacterium.prausnitzii_G	Yes	0.22751	0.055193	49	0	0.000168	0.00288
Previous_PTB	GCA.900066135.sp900066135	Yes	0.22751	0.055193	49	0	0.000168	0.00288
Previous_PTB	Gemmiger.sp003476825	Yes	0.22751	0.055193	49	0	0.000168	0.00288
Previous_PTB	Gemmiger.variabile	Yes	0.22751	0.055193	49	0	0.000168	0.00288
Previous_PTB	Parasutterella.excrementihominis	Yes	0.22751	0.055193	49	0	0.000168	0.00288
Previous_PTB	Roseburia.hominis	Yes	0.22751	0.055193	49	0	0.000168	0.00288
Previous_PTB	Ruminococcus_A.sp003011855	Yes	0.22751	0.055193	49	0	0.000168	0.00288

Previous_PTB	UBA11524.sp000437595	Yes	0.22751	0.055193	49	0	0.000168	0.00288
Delivery	DNF00809.sp001552935	PTB	0.698342	0.171029	49	0	0.00019	0.003231
Delivery	Prevotella.bergensis	PTB	0.102104	0.025009	49	0	0.00019	0.003231
Outcome_group	Lactobacillus_H.vaginalis	no-risk_PTB	2.616953	0.647005	49	0	0.000214	0.00362
LLETZ	Delftia.tsuruhatensis	Yes	0.305389	0.076279	49	0	0.000242	0.004095
Delivery	Prevotella.timonensis	PTB	0.651093	0.164439	49	0	0.000278	0.004672
Previous_PTB	Bacillus.atrophaeus	Yes	0.367338	0.092834	49	0	0.00028	0.004693
LLETZ	Cutibacterium.humerusii	Yes	0.328101	0.08296	49	0	0.000281	0.004705
Previous_PTB	Salinicola.sp003206695	Yes	-0.1978	0.050058	49	0	0.000284	0.00474
Previous_PTB	Delftia.tsuruhatensis	Yes	0.312945	0.079347	49	0	0.000291	0.004831
Delivery	Anaerococcus.tetradius	PTB	0.107347	0.027232	49	0	0.000293	0.004845
LLETZ	Bilophila.wadsworthia	Yes	0.234502	0.059523	49	0	0.000295	0.004845
LLETZ	Pseudoflavonifractor.sp	Yes	0.234	0.059	49	0	0.000	0.004

	900079765		502	523			295	845
Previous_PTB	Cutibacterium.humerusii	Yes	0.338188	0.086296	49	0	0.000314	0.005145
Previous_PTB	Faecalibacterium.prausnitzii_H	Yes	0.184686	0.047305	49	0	0.000329	0.005364
LLETZ	HRBIN23.sp002923335	Yes	0.437591	0.112599	49	0	0.000347	0.005645
LLETZ	Faecalibacterium.prausnitzii_H	Yes	0.176008	0.045476	49	0	0.000364	0.005904
Risk_group	X2.01.FULL.66.17.sp001803875	Risk	-0.0936	0.024767	49	0	0.00048	0.005939
Risk_group	Achromobacter.sp001299435	Risk	-0.0936	0.024767	49	0	0.00048	0.005939
LLETZ	Actinotignum.urinale	Yes	0.168492	0.044249	49	0	0.00044	0.005939
Previous_PTB	Actinotignum.urinale	Yes	0.177426	0.046028	49	0	0.000382	0.005939
Risk_group	Agrobacterium.sp900013535	Risk	-0.0936	0.024767	49	0	0.00048	0.005939
Risk_group	Akkermansia.glycaniphila	Risk	-0.0936	0.024767	49	0	0.00048	0.005939
Risk_group	Aliarcobacter.cryaerophilus_A	Risk	-0.0936	0.024767	49	0	0.00048	0.005939

Risk_group	Alistipes.onderdonkii	Risk	-0.0936	0.024767	49	0	0.00048	0.005939
Risk_group	Alistipes.senegalensis	Risk	-0.0936	0.024767	49	0	0.00048	0.005939
Risk_group	Alistipes_A.indistinctus	Risk	-0.0936	0.024767	49	0	0.00048	0.005939
Risk_group	ASF356.sp000364165	Risk	-0.0936	0.024767	49	0	0.00048	0.005939
LLETZ	Bacillus.atrophaeus	Yes	0.342008	0.089245	49	0	0.000409	0.005939
Risk_group	Bact.08.sp000473305	Risk	-0.0936	0.024767	49	0	0.00048	0.005939
Risk_group	Bacteroides_A.plebeius	Risk	-0.0936	0.024767	49	0	0.00048	0.005939
Risk_group	Baumannia.cicadellinicola_C	Risk	-0.0936	0.024767	49	0	0.00048	0.005939
Risk_group	Blastococcus.sp003075095	Risk	-0.0936	0.024767	49	0	0.00048	0.005939
Risk_group	Blautia_A.sp000285855	Risk	-0.0936	0.024767	49	0	0.00048	0.005939
Risk_group	Brevundimonas.sp002157625	Risk	-0.093	0.024767	49	0	0.00048	0.005939

			6					
Risk_group	Brevundimonas.sp002484265	Risk	-0.0936	0.024767	49	0	0.00048	0.005939
Risk_group	Butyricicoccus.pullicaecorum	Risk	-0.0936	0.024767	49	0	0.00048	0.005939
Risk_group	Butyricimonas.sp002161485	Risk	-0.0936	0.024767	49	0	0.00048	0.005939
Risk_group	Butyrivibrio_A.crossotus	Risk	-0.0936	0.024767	49	0	0.00048	0.005939
Risk_group	CAG.110.sp002437585	Risk	-0.0936	0.024767	49	0	0.00048	0.005939
Risk_group	CAG.194.sp000432915	Risk	-0.0936	0.024767	49	0	0.00048	0.005939
Risk_group	CAG.302.sp002375205	Risk	-0.0936	0.024767	49	0	0.00048	0.005939
Risk_group	CAG.83.sp000435555	Risk	-0.0936	0.024767	49	0	0.00048	0.005939
Risk_group	CAG.83.sp001916855	Risk	-0.0936	0.024767	49	0	0.00048	0.005939
Risk_group	Campylobacter_D.jejuni	Risk	-0.0936	0.024767	49	0	0.00048	0.005939

Risk_group	Clostridium.taeniosporum	Risk	-0.0936	0.024767	49	0	0.00048	0.005939
Risk_group	Clostridium_AN.bornimense	Risk	-0.0936	0.024767	49	0	0.00048	0.005939
Risk_group	Clostridium_Q.sp003024715	Risk	-0.0936	0.024767	49	0	0.00048	0.005939
Risk_group	Corynebacterium.appendicis	Risk	-0.0936	0.024767	49	0	0.00048	0.005939
Risk_group	Corynebacterium.kefirresidentii	Risk	-0.0936	0.024767	49	0	0.00048	0.005939
Risk_group	Corynebacterium.otitidis	Risk	-0.0936	0.024767	49	0	0.00048	0.005939
Risk_group	Cupriavidus.gilardii	Risk	-0.0936	0.024767	49	0	0.00048	0.005939
Risk_group	Dorea.formicigenerans	Risk	-0.0936	0.024767	49	0	0.00048	0.005939
Risk_group	Dorea.sp900066555	Risk	-0.0936	0.024767	49	0	0.00048	0.005939
Risk_group	DTU078.sp002385415	Risk	-0.0936	0.024767	49	0	0.00048	0.005939
Risk_grou	Erysipelatoclostridium.s	Risk	-0.093	0.024	49	0	0.000	0.005

p	piroforme		6	767			48	939
Risk_group	Eubacterium_E.hallii_A	Risk	-0.0936	0.024767	49	0	0.00048	0.005939
Risk_group	Eubacterium_H.cellulosolvens	Risk	-0.0936	0.024767	49	0	0.00048	0.005939
Risk_group	Eubacterium_S.uniforme	Risk	-0.0936	0.024767	49	0	0.00048	0.005939
Risk_group	Faecalibacterium.prausnitzii_C	Risk	-0.0936	0.024767	49	0	0.00048	0.005939
Risk_group	Faecalibacterium.prausnitzii_D	Risk	-0.0936	0.024767	49	0	0.00048	0.005939
Risk_group	Fastidiosipila.sanguinis	Risk	-0.0936	0.024767	49	0	0.00048	0.005939
Risk_group	Flavonifractor.sp002161085	Risk	-0.0936	0.024767	49	0	0.00048	0.005939
Risk_group	GCA.900066575.sp900066385	Risk	-0.0936	0.024767	49	0	0.00048	0.005939
Risk_group	Gordonia.jacobaea	Risk	-0.0936	0.024767	49	0	0.00048	0.005939
Risk_group	GWC2.39.14.sp000996245	Risk	-0.0936	0.024767	49	0	0.00048	0.005939

Risk_group	Hungatella.effluvii	Risk	-0.0936	0.024767	49	0	0.00048	0.005939
Risk_group	Ileibacterium.valens	Risk	-0.0936	0.024767	49	0	0.00048	0.005939
Risk_group	KLE1615.sp900066985	Risk	-0.0936	0.024767	49	0	0.00048	0.005939
Risk_group	Lysinibacillus.fusiformis_D	Risk	-0.0936	0.024767	49	0	0.00048	0.005939
Risk_group	Marvinbryantia.sp900066075	Risk	-0.0936	0.024767	49	0	0.00048	0.005939
Risk_group	Methylobacterium.organophilum	Risk	-0.0936	0.024767	49	0	0.00048	0.005939
Risk_group	Micromonospora.sp003176735	Risk	-0.0936	0.024767	49	0	0.00048	0.005939
Risk_group	Negativibacillus.massiliensis	Risk	-0.0936	0.024767	49	0	0.00048	0.005939
Risk_group	OEMS01.sp900199405	Risk	-0.0936	0.024767	49	0	0.00048	0.005939
LLETZ	Oscillibacter.sp000403435	Yes	0.236424	0.061481	49	0	0.000393	0.005939
Risk_group	Oxalobacter.formigenes_B	Risk	-0.093	0.024767	49	0	0.00048	0.005939

			6					
Risk_group	Paludibacter_A.jiangxiensis	Risk	-0.0936	0.024767	49	0	0.00048	0.005939
Risk_group	Parabacteroides.timonensis	Risk	-0.0936	0.024767	49	0	0.00048	0.005939
Risk_group	Parasporobacterium.paucivorans	Risk	-0.0936	0.024767	49	0	0.00048	0.005939
Risk_group	PeH17.sp000435055	Risk	-0.35521	0.092024	49	0	0.000376	0.005939
Risk_group	Prevotella.sp002251365	Risk	-0.0936	0.024767	49	0	0.00048	0.005939
Risk_group	Pseudomonas_E.hunanensis	Risk	-0.0936	0.024767	49	0	0.00048	0.005939
Risk_group	Ralstonia.pickettii	Risk	-0.0936	0.024767	49	0	0.00048	0.005939
Risk_group	Ralstonia.pseudosolanacearum	Risk	-0.0936	0.024767	49	0	0.00048	0.005939
Risk_group	RC9.sp000434935	Risk	-0.0936	0.024767	49	0	0.00048	0.005939
Risk_group	Rubellimicrobium.thermophilum	Risk	-0.0936	0.024767	49	0	0.00048	0.005939

Risk_group	RUG210.sp003265305	Risk	-0.0936	0.024767	49	0	0.00048	0.005939
Risk_group	RUG350.sp900315995	Risk	-0.0936	0.024767	49	0	0.00048	0.005939
Risk_group	Ruminococcus_F.champanellensis	Risk	-0.0936	0.024767	49	0	0.00048	0.005939
Risk_group	Soleaferrea.massiliensis	Risk	-0.0936	0.024767	49	0	0.00048	0.005939
Risk_group	Sporobacter.termitidis	Risk	-0.0936	0.024767	49	0	0.00048	0.005939
Risk_group	Stenotrophomonas.maltophilia	Risk	-0.0936	0.024767	49	0	0.00048	0.005939
Risk_group	Stenotrophomonas.rhizophila_A	Risk	-0.0936	0.024767	49	0	0.00048	0.005939
Risk_group	Streptococcus.thermophilus	Risk	-0.0936	0.024767	49	0	0.00048	0.005939
Risk_group	Streptomyces.papulosa	Risk	-0.0936	0.024767	49	0	0.00048	0.005939
Risk_group	Sutterella.wadsworthensis_B	Risk	-0.0936	0.024767	49	0	0.00048	0.005939
Risk_grou	SZUA.51.sp003232965	Risk	-0.093	0.024	49	0	0.000	0.005

p			6	767			48	939
Risk_group	Tepidanaerobacter.syntrophicus	Risk	-0.0936	0.024767	49	0	0.00048	0.005939
Risk_group	TF01.11.sp003524945	Risk	-0.0936	0.024767	49	0	0.00048	0.005939
Risk_group	Tidjanibacter.inops	Risk	-0.0936	0.024767	49	0	0.00048	0.005939
Risk_group	UBA11774.sp003507655	Risk	-0.0936	0.024767	49	0	0.00048	0.005939
Risk_group	UBA1191.sp900066305	Risk	-0.0936	0.024767	49	0	0.00048	0.005939
Risk_group	UBA1259.sp900320065	Risk	-0.0936	0.024767	49	0	0.00048	0.005939
Risk_group	UBA3566.sp002376065	Risk	-0.0936	0.024767	49	0	0.00048	0.005939
Risk_group	UBA7524.sp002478275	Risk	-0.0936	0.024767	49	0	0.00048	0.005939
Risk_group	UBA9655.sp001767835	Risk	-0.0936	0.024767	49	0	0.00048	0.005939
Risk_group	Veillonella.rogosae	Risk	-0.0936	0.024767	49	0	0.00048	0.005939

Risk_group	W11650.sp002999035	Risk	-0.0936	0.024767	49	0	0.00048	0.005939
Previous_PTB	GWF2.35.48.sp001769125	Yes	0.084758	0.022522	49	0	0.000503	0.006182
Previous_PTB	Methanobrevibacter.ruminantium	Yes	0.084758	0.022522	49	0	0.000503	0.006182
Previous_PTB	TMED.70.sp002171675	Yes	0.084758	0.022522	49	0	0.000503	0.006182
Delivery	Bifidobacterium.vaginale_E	PTB	0.750174	0.200071	49	0	0.000524	0.006408
Previous_PTB	Blautia_A.sp900066145	Yes	0.306564	0.081752	49	0	0.000524	0.006408
Delivery	X28L.sp002892445	PTB	0.251108	0.067993	49	0	0.000621	0.007566
Risk_group	Mycolicibacterium.sp001665685	Risk	-0.52697	0.143251	49	0	0.000648	0.00788
Delivery	Fannyhessea.vaginae_A	PTB	0.265381	0.072234	49	0	0.000657	0.007972
LLETZ	Coraliomargarita.akajimensis	Yes	0.268237	0.073326	49	0	0.000689	0.008333
Delivery	Prevotella.buccalis	PTB	0.209837	0.057649	49	0	0.000727	0.008774
Delivery	Parvimonas.sp001552895	PTB	0.289622	0.0797	49	0	0.00074	0.008909
Delivery	X28L.sp000177555	PTB	0.806	0.222	49	0	0.000	0.009

			192	178			752	028
LLETZ	CAG.45.sp900066395	Yes	0.282422	0.078001	49	0	0.00077	0.009194
Previous_PTB	HRBIN23.sp002923335	Yes	0.424166	0.117128	49	0	0.000768	0.009194
LLETZ	CAG.269.sp001916055	Yes	0.258636	0.071575	49	0	0.000786	0.00937
Risk_group	GWF2.35.48.sp001769125	Risk	-0.08773	0.02437	49	0	0.000818	0.00968
Risk_group	Methanobrevibacter.ruminantium	Risk	-0.08773	0.02437	49	0	0.000818	0.00968
Risk_group	TMED.70.sp002171675	Risk	-0.08773	0.02437	49	0	0.000818	0.00968
Risk_group	Salinicola.sp003206695	Risk	0.194619	0.054165	49	0	0.000835	0.009853
LLETZ	Ureaplasma.urealyticum	Yes	0.298074	0.083471	49	0	0.000891	0.01049
LLETZ	Caloramator.mitchellensis	Yes	0.459771	0.128949	49	0	0.000905	0.010633
LLETZ	Blautia_A.sp900066165	Yes	0.293655	0.082395	49	0	0.00091	0.010655
Previous_PTB	CAG.110.sp003525905	Yes	0.263741	0.074026	49	0	0.000913	0.010666
LLETZ	CAG.590.sp003530125	Yes	-0.117	0.032966	49	0	0.000926	0.010791

LLETZ	Stenotrophomonas.maltophilia_AM	Yes	0.503236	0.141711	49	0	0.000944	0.010984
Previous_PTB	Helicobacter.pylori	Yes	0.197029	0.055552	49	0	0.000957	0.011101
Risk_group	Alistipes.putredinis	Risk	-0.31541	0.089387	49	0	0.001009	0.011651
LLETZ	Nocardiopsis.sp900143625	Yes	0.34383	0.097436	49	0	0.001008	0.011651
LLETZ	CAG.196.sp002102975	Yes	0.277596	0.079141	49	0	0.001073	0.012356
Delivery	Prevotella.sp000479005	PTB	0.259485	0.074234	49	0	0.001111	0.012769
Delivery	Prevotella.jejuni	PTB	0.155996	0.04481	49	0	0.001158	0.013248
Risk_group	TF01.11.sp001414325	Risk	-0.28527	0.081947	49	0	0.001158	0.013248
LLETZ	Faecalicatena.lactaris	Yes	0.359502	0.103574	49	0	0.001193	0.013615
Previous_PTB	Stenotrophomonas.maltophilia_AM	Yes	0.510273	0.14741	49	0	0.001226	0.013958
Delivery	Prevotella.timonensis_A	PTB	0.600839	0.173694	49	0	0.001235	0.014022
LLETZ	Caecibacter.massiliensis	Yes	0.279517	0.080984	49	0	0.001263	0.014303

Delivery	Sneathia.amnii	PTB	0.299569	0.087813	49	0	0.001417	0.01602
Risk_group	Alistipes.sp000434235	Risk	-0.24643	0.072523	49	0	0.001474	0.016348
Risk_group	Bacteroides.helcogenes	Risk	-0.24643	0.072523	49	0	0.001474	0.016348
Risk_group	CAG.462.sp900291465	Risk	-0.24643	0.072523	49	0	0.001474	0.016348
Risk_group	Coprobacter.fastidiosus	Risk	-0.24643	0.072523	49	0	0.001474	0.016348
Risk_group	Parabacteroides.sp900155425	Risk	-0.24643	0.072523	49	0	0.001474	0.016348
Risk_group	RUG762.sp900316495	Risk	-0.24643	0.072523	49	0	0.001474	0.016348
Risk_group	Senegalimassilia.anaerobia	Risk	-0.24643	0.072523	49	0	0.001474	0.016348
Risk_group	Turicibacter.sp001543345	Risk	-0.24643	0.072523	49	0	0.001474	0.016348
Previous_PTB	Prevotella.corporis	Yes	-0.30666	0.090338	49	0	0.001488	0.01647
LLETZ	CAG.81.sp900066785	Yes	0.401941	0.118503	49	0	0.0015	0.016561

Previous_PTB	Caloramator.mitchellensis	Yes	0.454094	0.134135	49	0	0.001528	0.016834
Delivery	Amphibacillus.xylanus	PTB	0.080718	0.023909	49	0	0.001569	0.017252
Risk_group	Delftia.tsuruhatensis	Risk	-0.28905	0.085857	49	0	0.001612	0.017682
LLETZ	Staphylococcus.capitis	Yes	0.274818	0.082376	49	0	0.001759	0.019244
Previous_PTB	X32.111.sp001508335	Yes	0.12433	0.03748	49	0	0.001856	0.019342
Previous_PTB	Akkermansia.sp001580195	Yes	0.12433	0.03748	49	0	0.001856	0.019342
Previous_PTB	Alistipes_A.sp900240235	Yes	0.12433	0.03748	49	0	0.001856	0.019342
Previous_PTB	Anaerosporobacter.mobilis	Yes	0.12433	0.03748	49	0	0.001856	0.019342
Previous_PTB	Bifidobacterium.animalis	Yes	0.12433	0.03748	49	0	0.001856	0.019342
Previous_PTB	Bilophila.wadsworthia	Yes	0.206267	0.061917	49	0	0.001783	0.019342
Previous_PTB	CAG.313.sp003539625	Yes	0.12433	0.03748	49	0	0.001856	0.019342
Previous_PTB	Eubacterium_F.sp000687695	Yes	0.12433	0.03748	49	0	0.001856	0.019342
Previous_PTB	Faecalibacterium.prausnitzii_K	Yes	0.12433	0.03748	49	0	0.001856	0.019342

Previous_ PTB	Faecalicatena.faecis	Yes	0.124 33	0.037 48	49	0	0.001 856	0.019 342
Previous_ PTB	Neoehrlichia.lotoris	Yes	0.124 33	0.037 48	49	0	0.001 856	0.019 342
Previous_ PTB	Oscillibacter.ruminantiu m	Yes	0.124 33	0.037 48	49	0	0.001 856	0.019 342
Previous_ PTB	Pseudoflavonifractor.sp 900079765	Yes	0.206 267	0.061 917	49	0	0.001 783	0.019 342
Previous_ PTB	QALS01.sp003150575	Yes	0.124 33	0.037 48	49	0	0.001 856	0.019 342
Previous_ PTB	Raoultella.planticola	Yes	0.124 33	0.037 48	49	0	0.001 856	0.019 342
Previous_ PTB	Rikenella.microfusus	Yes	0.124 33	0.037 48	49	0	0.001 856	0.019 342
Previous_ PTB	Ruminococcus_E.bromi i	Yes	0.124 33	0.037 48	49	0	0.001 856	0.019 342
Previous_ PTB	Ruthenibacterium.lactati formans	Yes	0.124 33	0.037 48	49	0	0.001 856	0.019 342
Previous_ PTB	Shuttleworthia.satelles	Yes	0.124 33	0.037 48	49	0	0.001 856	0.019 342
Previous_ PTB	SK.Y3.sp002252565	Yes	0.124 33	0.037 48	49	0	0.001 856	0.019 342
Delivery	Sneathia.sanguinegens	PTB	0.541 659	0.162 948	49	0	0.001 82	0.019 342
Previous_ PTB	Turicibacter.sanguinis	Yes	0.124 33	0.037 48	49	0	0.001 856	0.019 342

Risk_group	Cutibacterium.humerusii	Risk	-0.30863	0.093377	49	0	0.00192	0.019969
Previous_PTB	Staphylococcus.capitis	Yes	0.282914	0.085688	49	0	0.00194	0.02013
LLETZ	Staphylococcus.auricularis	Yes	0.12141	0.036832	49	0	0.001969	0.020392
Previous_PTB	Bifidobacterium.scardovii	Yes	-0.31564	0.096312	49	0	0.002078	0.021476
LLETZ	Bacteroides.ovatus	Yes	0.414697	0.126728	49	0	0.002108	0.021732
LLETZ	Helicobacter.pylori_C	Yes	0.306905	0.093849	49	0	0.002121	0.021818
Previous_PTB	Agathobacter.faecis	Yes	0.116875	0.036424	49	0	0.002521	0.024718
Previous_PTB	Agathobacter.rectale	Yes	0.116875	0.036424	49	0	0.002521	0.024718
Previous_PTB	Bacteroides.uniformis	Yes	0.116875	0.036424	49	0	0.002521	0.024718
Risk_group	Acetatifactor.sp900066365	Risk	-0.19168	0.059721	49	0	0.002515	0.024718
Risk_group	Alistipes.sp900021155	Risk	-0.19168	0.059721	49	0	0.002515	0.024718
Risk_group	Bacteroides.ndongoniae	Risk	-0.19168	0.059721	49	0	0.002515	0.024718

Risk_group	Bacteroides.sp002160055	Risk	-0.19168	0.059721	49	0	0.002515	0.024718
Delivery	Bifidobacterium.vaginale_A	PTB	0.253033	0.078619	49	0	0.002453	0.024718
Risk_group	Blautia_A.wexlerae	Risk	-0.19168	0.059721	49	0	0.002515	0.024718
Risk_group	Brevundimonas.sp002434505	Risk	-0.19168	0.059721	49	0	0.002515	0.024718
Risk_group	CAG.354.sp001915925	Risk	-0.19168	0.059721	49	0	0.002515	0.024718
Risk_group	CAG.56.sp900066615	Risk	-0.19168	0.059721	49	0	0.002515	0.024718
Previous_PTB	Cutibacterium.granulosum	Yes	0.301245	0.093476	49	0	0.002424	0.024718
Risk_group	Delftia.acidovorans	Risk	-0.19168	0.059721	49	0	0.002515	0.024718
Risk_group	F0540.sp000466585	Risk	-0.19168	0.059721	49	0	0.002515	0.024718
Risk_group	Faecalibacterium.prausnitzii_G	Risk	-0.19168	0.059721	49	0	0.002515	0.024718
Risk_group	GCA.900066135.sp900066135	Risk	-0.19168	0.059721	49	0	0.002515	0.024718

Risk_group	Gemmiger.sp003476825	Risk	-0.19168	0.059721	49	0	0.002515	0.024718
Risk_group	Gemmiger.variabile	Risk	-0.19168	0.059721	49	0	0.002515	0.024718
Risk_group	Parasutterella.excrementihominis	Risk	-0.19168	0.059721	49	0	0.002515	0.024718
Risk_group	Roseburia.hominis	Risk	-0.19168	0.059721	49	0	0.002515	0.024718
Risk_group	Ruminococcus_A.sp003011855	Risk	-0.19168	0.059721	49	0	0.002515	0.024718
Risk_group	UBA11524.sp000437595	Risk	-0.19168	0.059721	49	0	0.002515	0.024718
Previous_PTB	Faecalicatena.lactaris	Yes	0.345191	0.10774	49	0	0.002555	0.025001
LLETZ	Eisenbergiella.sp900066775	Yes	0.278976	0.087109	49	0	0.002564	0.025043
Outcome_group	Lactobacillus.gasseri	no-risk_PTB	4.529959	1.424649	49	0	0.002733	0.026589
Previous_PTB	Oscillibacter.sp000403435	Yes	0.203385	0.063954	49	0	0.00273	0.026589
Risk_group	Bacillus.atrophaeus	Risk	-0.31932	0.100451	49	0	0.00274	0.026595

LLETZ	X32.111.sp001508335	Yes	0.113526	0.036031	49	0	0.002962	0.027694
LLETZ	Akkermansia.sp001580195	Yes	0.113526	0.036031	49	0	0.002962	0.027694
LLETZ	Alistipes_A.sp900240235	Yes	0.113526	0.036031	49	0	0.002962	0.027694
LLETZ	Anaerosporobacter.mobilis	Yes	0.113526	0.036031	49	0	0.002962	0.027694
LLETZ	Bifidobacterium.animalis	Yes	0.113526	0.036031	49	0	0.002962	0.027694
LLETZ	CAG.313.sp003539625	Yes	0.113526	0.036031	49	0	0.002962	0.027694
LLETZ	Eubacterium_F.sp000687695	Yes	0.113526	0.036031	49	0	0.002962	0.027694
LLETZ	Faecalibacterium.prausnitzii_K	Yes	0.113526	0.036031	49	0	0.002962	0.027694
LLETZ	Faecalicatena.faecis	Yes	0.113526	0.036031	49	0	0.002962	0.027694
LLETZ	Neoehrlichia.lotoris	Yes	0.113526	0.036031	49	0	0.002962	0.027694
LLETZ	Oscillibacter.ruminantium	Yes	0.113526	0.036031	49	0	0.002962	0.027694
LLETZ	QALS01.sp003150575	Yes	0.113526	0.036031	49	0	0.002962	0.027694
LLETZ	Raoultella.planticola	Yes	0.113526	0.036031	49	0	0.002962	0.027694

LLETZ	Rikenella.microfusus	Yes	0.113526	0.036031	49	0	0.002962	0.027694
LLETZ	Ruminococcus_E.bromii	Yes	0.113526	0.036031	49	0	0.002962	0.027694
LLETZ	Ruthenibacterium.lactatiformans	Yes	0.113526	0.036031	49	0	0.002962	0.027694
LLETZ	Shuttleworthia.satelles	Yes	0.113526	0.036031	49	0	0.002962	0.027694
LLETZ	SK.Y3.sp002252565	Yes	0.113526	0.036031	49	0	0.002962	0.027694
LLETZ	Turicibacter.sanguinis	Yes	0.113526	0.036031	49	0	0.002962	0.027694
Previous_PTB	CAG.269.sp001916055	Yes	0.233089	0.074454	49	0	0.003132	0.029226
LLETZ	CAG.452.sp000434035	Yes	0.433227	0.13904	49	0	0.003263	0.030389
LLETZ	Veillonella.montpellierensis	Yes	0.181225	0.058214	49	0	0.003288	0.030564
Delivery	Aerococcus.christensenii	PTB	0.654685	0.213594	49	0	0.003751	0.034802
Delivery	Bifidobacterium.vaginale_C	PTB	0.620222	0.202806	49	0	0.003822	0.035327
Delivery	Dialister.sp001553355	PTB	0.213668	0.069852	49	0	0.003816	0.035327
LLETZ	Eubacterium_I.ramulus	Yes	0.271168	0.089034	49	0	0.003955	0.036486

LLETZ	Virgibacillus_G.profundi	Yes	0.516555	0.169831	49	0	0.004	0.036825
LLETZ	Agathobacter.faecis	Yes	0.105809	0.035016	49	0	0.004222	0.038566
LLETZ	Agathobacter.rectale	Yes	0.105809	0.035016	49	0	0.004222	0.038566
LLETZ	Bacteroides.uniformis	Yes	0.105809	0.035016	49	0	0.004222	0.038566
Previous_PTB	Helicobacter.pylori_C	Yes	0.294939	0.097624	49	0	0.004228	0.038566
Delivery	Finegoldia.magna_H	PTB	0.125291	0.041472	49	0	0.004229	0.038566
Outcome_group	Lactobacillus.taiwanensis	no-risk_PTB	2.619815	0.873129	49	0	0.004472	0.040708
Risk_group	Faecalibacterium.prausnitzii_H	Risk	-0.15283	0.051186	49	0	0.004654	0.042278
LLETZ	Parabacteroides.johnsonii	Yes	0.526389	0.178751	49	0	0.005196	0.047119
Risk_group	Blautia_A.sp900066145	Risk	-0.2603	0.088459	49	0	0.005227	0.047305
Delivery	Prevotella.colorans	PTB	0.397373	0.135189	49	0	0.005272	0.047629
Risk_group	Prevotella.corporis	Risk	0.287219	0.09775	49	0	0.005288	0.047678

Risk_group	Actinotignum.urinale	Risk	-0.14625	0.049805	49	0	0.005315	0.047835
Risk_group	UBA1033.sp001695555	Risk	-0.11725	0.04008	49	0	0.005472	0.049159
Delivery	Arcanobacterium_A.sp000758825	PTB	0.049648	0.017256	49	0	0.006223	0.051307
Delivery	Atopobium.deltae	PTB	0.049648	0.017256	49	0	0.006223	0.051307
Delivery	Bartonella.sp000278115	PTB	0.049648	0.017256	49	0	0.006223	0.051307
Previous_PTB	Bifidobacterium.callitrichidarum	Yes	-0.11309	0.038992	49	0	0.005851	0.051307
Previous_PTB	Bifidobacterium.myosotis	Yes	-0.14683	0.05051	49	0	0.00575	0.051307
Previous_PTB	Bifidobacterium.reuteri	Yes	-0.11309	0.038992	49	0	0.005851	0.051307
LLETZ	Bifidobacterium.scardovii	Yes	-0.26663	0.092589	49	0	0.006181	0.051307
Risk_group	Bifidobacterium.scardovii	Risk	0.300058	0.104214	49	0	0.006189	0.051307
Delivery	Clostridium.gasigenes	PTB	0.049648	0.017256	49	0	0.006223	0.051307
LLETZ	Cutibacterium.granulos	Yes	0.258	0.089	49	0	0.006	0.051

	um		735	862			189	307
Delivery	Dubosiella.sp000403415	PTB	0.049648	0.017256	49	0	0.006223	0.051307
Delivery	Facklamia.languida	PTB	0.049648	0.017256	49	0	0.006223	0.051307
Delivery	Fusobacterium_C.necrophorum	PTB	0.049648	0.017256	49	0	0.006223	0.051307
Delivery	GWA2.31.9.sp001768155	PTB	0.049648	0.017256	49	0	0.006223	0.051307
Delivery	Histophilus.somni	PTB	0.049648	0.017256	49	0	0.006223	0.051307
Delivery	KA00274.sp001552885	PTB	0.049648	0.017256	49	0	0.006223	0.051307
Delivery	Metamycoplasma.hominis	PTB	0.049648	0.017256	49	0	0.006223	0.051307
Delivery	Peptoanaerobacter.stomatis	PTB	0.049648	0.017256	49	0	0.006223	0.051307
Delivery	PGYU01.sp002839825	PTB	0.049648	0.017256	49	0	0.006223	0.051307
Delivery	Porphyromonas.asaccharolytica	PTB	0.049648	0.017256	49	0	0.006223	0.051307
Delivery	Porphyromonas.gingivalis	PTB	0.049648	0.017256	49	0	0.006223	0.051307
Delivery	Porphyromonas.somerae	PTB	0.049648	0.017256	49	0	0.006223	0.051307
Delivery	Porphyromonas.uenonis	PTB	0.049648	0.017256	49	0	0.006223	0.051307

Delivery	Porphyromonas.uenonis_A	PTB	0.049648	0.017256	49	0	0.006223	0.051307
Delivery	Prevotella.amnii	PTB	0.049648	0.017256	49	0	0.006223	0.051307
Delivery	Prevotella.denticola	PTB	0.049648	0.017256	49	0	0.006223	0.051307
Delivery	Prevotella.enoeca	PTB	0.049648	0.017256	49	0	0.006223	0.051307
Delivery	Prevotella.histicola	PTB	0.049648	0.017256	49	0	0.006223	0.051307
Delivery	Prevotella.ihumii	PTB	0.049648	0.017256	49	0	0.006223	0.051307
Delivery	Prevotella.intermedia	PTB	0.049648	0.017256	49	0	0.006223	0.051307
Delivery	Prevotella.melaninogenica	PTB	0.049648	0.017256	49	0	0.006223	0.051307
Delivery	Prevotella.nanceiensis	PTB	0.049648	0.017256	49	0	0.006223	0.051307
Delivery	Prevotella.nigrescens	PTB	0.049648	0.017256	49	0	0.006223	0.051307
Delivery	Prevotella.oris	PTB	0.049648	0.017256	49	0	0.006223	0.051307
Delivery	Prevotella.oulorum	PTB	0.049648	0.017256	49	0	0.006223	0.051307
Delivery	Prevotella.pallens	PTB	0.049648	0.017256	49	0	0.006223	0.051307

Delivery	Prevotella.pleuritidis	PTB	0.049648	0.017256	49	0	0.006223	0.051307
Delivery	Prevotella.scopos	PTB	0.049648	0.017256	49	0	0.006223	0.051307
Delivery	Prevotella.sp000163055	PTB	0.049648	0.017256	49	0	0.006223	0.051307
Delivery	Prevotella.sp000758925	PTB	0.049648	0.017256	49	0	0.006223	0.051307
Delivery	Prevotella.sp001552765	PTB	0.049648	0.017256	49	0	0.006223	0.051307
Delivery	Prevotella.sp001553265	PTB	0.049648	0.017256	49	0	0.006223	0.051307
Delivery	Prevotella.sp002251295	PTB	0.049648	0.017256	49	0	0.006223	0.051307
Delivery	Prevotella.sp003043945	PTB	0.049648	0.017256	49	0	0.006223	0.051307
Delivery	Prevotella.sp900199655	PTB	0.049648	0.017256	49	0	0.006223	0.051307
Delivery	Streptococcus.equi	PTB	0.049648	0.017256	49	0	0.006223	0.051307
Delivery	Streptococcus.pyogenes	PTB	0.049648	0.017256	49	0	0.006223	0.051307
Delivery	Tenacibaculum.agarivorans	PTB	0.049648	0.017256	49	0	0.006223	0.051307
Previous_PTB	Alistipes.finegoldii	Yes	0.166301	0.058094	49	0	0.006467	0.05323

LLETZ	Desulfuromonas_D.acetexigens	Yes	0.524241	0.183254	49	0	0.0065	0.053407
LLETZ	Eubacterium_F.xylanophilum	Yes	0.25393	0.088792	49	0	0.006515	0.053443
Outcome_group	Ureaplasma.parvum	no-risk_PTB	1.561247	0.548489	49	0	0.006749	0.055269
LLETZ	UBA1033.sp001695555	Yes	0.10083	0.035609	49	0	0.007018	0.057371
Outcome_group	Fannyhessea.vaginae	no-risk_PTB	1.371393	0.485163	49	0	0.00711	0.058024
Delivery	Prevotella.corporis	PTB	0.172711	0.061521	49	0	0.007479	0.060933
Previous_PTB	CAG.45.sp900066395	Yes	0.227589	0.081138	49	0	0.007526	0.061112
Risk_group	Helicobacter.pylori	Risk	-0.16862	0.06011	49	0	0.007522	0.061112
LLETZ	Prevotella.copri	Yes	0.127688	0.04587	49	0	0.007956	0.064383
LLETZ	Slackia_A.piriformis	Yes	0.127688	0.04587	49	0	0.007956	0.064383
Risk_group	CAG.110.sp003525905	Risk	-0.22146	0.080099	49	0	0.008356	0.06751
Delivery	Empedobacter.falsenii	PTB	0.088986	0.03221	49	0	0.008401	0.06776

LLETZ	Ruminococcus_C.sp000433635	Yes	0.434554	0.159298	49	0	0.009192	0.073887
Delivery	Gemella_A.asaccharolytica	PTB	0.164245	0.060203	49	0	0.009186	0.073887
LLETZ	Ruminococcus_E.bromii_B	Yes	0.3079	0.113287	49	0	0.009433	0.075697
LLETZ	Amphibacillus.xylanus	Yes	0.091696	0.033751	49	0	0.009458	0.075773
LLETZ	Clostridium_H.botulinum_A	Yes	0.180382	0.066431	49	0	0.009494	0.075937
LLETZ	Chryseobacterium.oncorhynchi	Yes	0.418288	0.154422	49	0	0.009657	0.076981
LLETZ	Alistipes.finegoldii	Yes	0.151291	0.055848	49	0	0.00965	0.076981
Delivery	Prevotella.disiens	PTB	0.230797	0.085797	49	0	0.010131	0.080626
LLETZ	Bifidobacterium.callitrichidarum	Yes	-0.10021	0.037485	49	0	0.010574	0.08386
LLETZ	Bifidobacterium.reuteri	Yes	-0.10021	0.037485	49	0	0.010574	0.08386
LLETZ	UBA690.sp003487005	Yes	0.129609	0.048494	49	0	0.010589	0.08386
LLETZ	Alistipes.obesi	Yes	0.276505	0.103628	49	0	0.01071	0.084583
LLETZ	Erysipelatoclostridium.s	Yes	0.256	0.096	49	0	0.010	0.084

	p003024675		82	259			716	583
Risk_group	HRBIN23.sp002923335	Risk	-0.33456	0.126738	49	0	0.011512	0.090721
LLETZ	CAG.217.sp000436335	Yes	0.292325	0.110867	49	0	0.011601	0.091276
Delivery	Stomatobaculum.sp002892395	PTB	0.229072	0.087068	49	0	0.011772	0.092468
Delivery	Bifidobacterium.vaginale_D	PTB	0.344719	0.131974	49	0	0.012347	0.096829
LLETZ	Empedobacter.falsenii	Yes	0.11818	0.045468	49	0	0.012752	0.09984
LLETZ	Staphylococcus_A.sciuri	Yes	0.111931	0.043112	49	0	0.012844	0.100398
LLETZ	Micrococcus.luteus	Yes	0.145994	0.056267	49	0	0.012898	0.100651
Risk_group	Caloramator.mitchellensis	Risk	-0.37476	0.14514	49	0	0.013311	0.103712
Previous_PTB	Amphibacillus.xylanus	Yes	0.090078	0.035109	49	0	0.013865	0.107855
Previous_PTB	Tepidimonas.taiwanensis	Yes	0.093685	0.036543	49	0	0.013934	0.108213
Previous_PTB	Coraliomargarita.akajimensis	Yes	0.19543	0.076275	49	0	0.013988	0.10846
LLETZ	Prevotella.sp001275135	Yes	0.427682	0.167371	49	0	0.014229	0.109975

Previous_PTB	Eubacterium_F.xylanophilum	Yes	0.236054	0.092363	49	0	0.014214	0.109975
LLETZ	Prevotella.sp002350805	Yes	0.261989	0.103149	49	0	0.014783	0.114074
Previous_PTB	CAG.217.sp000436335	Yes	0.292365	0.115325	49	0	0.014959	0.115248
Risk_group	Bilophila.wadsworthia	Risk	-0.16969	0.066997	49	0	0.015046	0.115543
Risk_group	Pseudoflavonifractor.sp900079765	Risk	-0.16969	0.066997	49	0	0.015046	0.115543
Outcome_group	Ureaplasma.urealyticum	no-risk_PTB	0.386234	0.153387	49	0	0.015605	0.119645
LLETZ	Tepidimonas.taiwanensis	Yes	0.088058	0.03513	49	0	0.016049	0.122853
Outcome_group	Bifidobacterium.longum	no-risk_PTB	1.869065	0.74654	49	0	0.016168	0.123569
Risk_group	Staphylococcus.capitis	Risk	-0.23127	0.092719	49	0	0.016544	0.126246
Risk_group	Helicobacter.pylori_C	Risk	-0.26333	0.105634	49	0	0.016602	0.126486
LLETZ	Anaerostipes.hadrus	Yes	0.222979	0.089595	49	0	0.01677	0.127561
LLETZ	Lactococcus.lactis_E	Yes	0.201	0.081	49	0	0.016	0.127

			673	085			835	852
Previous_PTB	CAG.196.sp002102975	Yes	0.204649	0.082324	49	0	0.016887	0.128052
Risk_group	CAG.269.sp001916055	Risk	-0.19956	0.080562	49	0	0.017257	0.130646
LLETZ	Bacillus_F.enclensis	Yes	0.477174	0.192805	49	0	0.017347	0.131123
Delivery	Eubacterium_B.sulci	PTB	0.077901	0.031517	49	0	0.017483	0.131948
Risk_group	Peptoniphilus_E.obesi	Risk	-0.12864	0.052221	49	0	0.017847	0.134478
Previous_PTB	Ruminococcus_E.bromii_B	Yes	0.290014	0.117843	49	0	0.017945	0.135006
Risk_group	Faecalicatena.lactaris	Risk	-0.28654	0.116579	49	0	0.01808	0.135809
Previous_PTB	Bifidobacterium.breve	Yes	-1.8149	0.739335	49	0	0.018219	0.136611
Previous_PTB	UBA1033.sp001695555	Yes	0.090907	0.037041	49	0	0.018243	0.136611
Delivery	Fannyhessea.vaginae	PTB	0.458525	0.187032	49	0	0.018361	0.137278
Risk_group	Alistipes.finegoldii	Risk	-0.15381	0.06286	49	0	0.018576	0.138671

LLETZ	UBA7862.sp002500135	Yes	0.46078	0.189902	49	0	0.019516	0.142708
Risk_group	X32.111.sp001508335	Risk	-0.09808	0.040555	49	0	0.019893	0.142708
Delivery	X4484.107.sp002059205	PTB	0.044031	0.018284	49	0	0.020391	0.142708
Risk_group	Akkermansia.sp001580195	Risk	-0.09808	0.040555	49	0	0.019893	0.142708
Risk_group	Alistipes_A.sp900240235	Risk	-0.09808	0.040555	49	0	0.019893	0.142708
Risk_group	Anaerosporobacter.mobilis	Risk	-0.09808	0.040555	49	0	0.019893	0.142708
Delivery	Bacillus_W.sp002585305	PTB	0.044031	0.018284	49	0	0.020391	0.142708
LLETZ	Bacteroides.cellulosilyticus	Yes	0.478769	0.198167	49	0	0.020012	0.142708
Risk_group	Bifidobacterium.animalis	Risk	-0.09808	0.040555	49	0	0.019893	0.142708
Risk_group	CAG.313.sp003539625	Risk	-0.09808	0.040555	49	0	0.019893	0.142708
Delivery	CAG.590.sp003530125	PTB	0.0565	0.023353	49	0	0.019852	0.142708
Delivery	CAG.791.sp900317475	PTB	0.044	0.018	49	0	0.020	0.142

			031	284			391	708
Delivery	Cetobacterium.ceti	PTB	0.044 031	0.018 284	49	0	0.020 391	0.142 708
Delivery	Chitinophaga.niastensis	PTB	0.044 031	0.018 284	49	0	0.020 391	0.142 708
Risk_grou p	Eubacterium_F.sp00068 7695	Risk	- 0.098 08	0.040 555	49	0	0.019 893	0.142 708
Delivery	Ezakiella.peruensis	PTB	0.044 031	0.018 284	49	0	0.020 391	0.142 708
Risk_grou p	Faecalibacterium.prausn itzii_K	Risk	- 0.098 08	0.040 555	49	0	0.019 893	0.142 708
Risk_grou p	Faecalicatena.faecis	Risk	- 0.098 08	0.040 555	49	0	0.019 893	0.142 708
Delivery	Fibrobacter.sp90021532 5	PTB	0.044 031	0.018 284	49	0	0.020 391	0.142 708
LLETZ	JB111.sp900163555	Yes	0.174 178	0.072 13	49	0	0.020 071	0.142 708
Delivery	Lactobacillus.sp002417 825	PTB	0.044 031	0.018 284	49	0	0.020 391	0.142 708
Delivery	Lancefieldella.rimae	PTB	0.044 031	0.018 284	49	0	0.020 391	0.142 708
Delivery	Lancefieldella.sp000564 995	PTB	0.044 031	0.018 284	49	0	0.020 391	0.142 708
Delivery	Maribacter.orientalis	PTB	0.044 031	0.018 284	49	0	0.020 391	0.142 708

Delivery	Mesorhizobium.sp002294725	PTB	0.044031	0.018284	49	0	0.020391	0.142708
Risk_group	Neoehrlichia.lotoris	Risk	-0.09808	0.040555	49	0	0.019893	0.142708
Risk_group	Oscillibacter.ruminantium	Risk	-0.09808	0.040555	49	0	0.019893	0.142708
Risk_group	Oscillibacter.sp000403435	Risk	-0.16671	0.069201	49	0	0.020351	0.142708
Delivery	Peptostreptococcus.sp000758885	PTB	0.044031	0.018284	49	0	0.020391	0.142708
Delivery	Prevotella.aurantiaca	PTB	0.044031	0.018284	49	0	0.020391	0.142708
Risk_group	QALS01.sp003150575	Risk	-0.09808	0.040555	49	0	0.019893	0.142708
Risk_group	Raoultella.planticola	Risk	-0.09808	0.040555	49	0	0.019893	0.142708
Risk_group	Rikenella.microfusus	Risk	-0.09808	0.040555	49	0	0.019893	0.142708
Risk_group	Ruminococcus_E.bromii	Risk	-0.09808	0.040555	49	0	0.019893	0.142708
Risk_group	Ruthenibacterium.lactatiformans	Risk	-0.09808	0.040555	49	0	0.019893	0.142708

Delivery	Scardovia.wiggsiae	PTB	0.044031	0.018284	49	0	0.020391	0.142708
Risk_group	Shuttleworthia.satelles	Risk	-0.09808	0.040555	49	0	0.019893	0.142708
Risk_group	SK.Y3.sp002252565	Risk	-0.09808	0.040555	49	0	0.019893	0.142708
Delivery	Streptococcus.infantarius	PTB	0.044031	0.018284	49	0	0.020391	0.142708
Delivery	Streptococcus.pseudopneumoniae_L	PTB	0.044031	0.018284	49	0	0.020391	0.142708
Risk_group	Turicibacter.sanguinis	Risk	-0.09808	0.040555	49	0	0.019893	0.142708
Delivery	Veillonella.parvula_A	PTB	0.044031	0.018284	49	0	0.020391	0.142708
Delivery	W5053.sp000467935	PTB	0.044031	0.018284	49	0	0.020391	0.142708
Previous_PTB	Caecibacter.massiliensis	Yes	0.201767	0.084241	49	0	0.021042	0.147051
Risk_group	Amphibacillus.xylanus	Risk	-0.0906	0.037989	49	0	0.021566	0.150492
Previous_PTB	Prevotella.sp001275135	Yes	0.414764	0.174102	49	0	0.021698	0.151192
Delivery	Bifidobacterium.bifidum	PTB	0.270573	0.113951	49	0	0.022108	0.15383

Risk_group	Stenotrophomonas.maltophilia_AM	Risk	-0.37861	0.159505	49	0	0.022151	0.153904
Previous_PTB	Bifidobacterium.infantis	Yes	-0.5684	0.239855	49	0	0.022356	0.155108
Outcome_group	Lactobacillus.johnsonii	no-risk_PTB	1.973156	0.835039	49	0	0.022723	0.157425
Risk_group	Streptococcus.pneumoniae_C	Risk	-0.13198	0.056129	49	0	0.023352	0.161552
Risk_group	CAG.45.sp900066395	Risk	-0.2054	0.087796	49	0	0.024023	0.165958
LLETZ	Alloprevotella.rava	Yes	0.482313	0.206303	49	0	0.024115	0.166351
Outcome_group	Helicobacter.pylori_C	no-risk_PTB	0.402228	0.172458	49	0	0.024434	0.168313
LLETZ	Dethiosulfatibacter.aminovorans	Yes	0.486204	0.208758	49	0	0.024625	0.169385
Risk_group	Agathobacter.faecis	Risk	-0.09132	0.039413	49	0	0.025334	0.173271
Risk_group	Agathobacter.rectale	Risk	-0.09132	0.039413	49	0	0.025334	0.173271
Risk_group	Bacteroides.uniformis	Risk	-0.09132	0.039413	49	0	0.025334	0.173271

Previous_PTB	Desulfuromonas_D.acetexigens	Yes	0.441684	0.190624	49	0	0.025331	0.173271
Previous_PTB	Eubacterium_I.ramulus	Yes	0.214291	0.092614	49	0	0.025524	0.174325
Previous_PTB	Lactobacillus_C.rhamnosus	Yes	0.584448	0.253828	49	0	0.026209	0.178613
LLETZ	Peptoniphilus_E.obesi	Yes	0.106814	0.046396	49	0	0.026226	0.178613
Risk_group	Bifidobacterium.myosotis	Risk	0.125764	0.054655	49	0	0.026299	0.178853
LLETZ	Faecalibacterium.prausnitzii_I	Yes	0.305765	0.133079	49	0	0.026512	0.180052
Risk_group	Bifidobacterium.breve	Risk	1.835733	0.799995	49	0	0.026695	0.181039
Previous_PTB	Erysipelatoclostridium.sp003024675	Yes	0.229171	0.10013	49	0	0.027069	0.183316
LLETZ	Intestinibacter.bartlettii	Yes	0.365722	0.162448	49	0	0.02953	0.199702
LLETZ	Photobacterium.sanguinicancri	Yes	0.212867	0.09476	49	0	0.029869	0.201711
Delivery	Lactobacillus.crispatus	PTB	-1.64796	0.734709	49	0	0.030103	0.203007
Delivery	Lactobacillus.johnsonii	PTB	-0.71769	0.32191	49	0	0.031057	0.208856
LLETZ	Coprococcus.eutactus_	Yes	0.435	0.195	49	0	0.031	0.208

	A		236	214			054	856
LLETZ	Duncaniella.muris	Yes	0.430356	0.193506	49	0	0.031452	0.211213
Risk_group	Bifidobacterium.callitrichidarum	Risk	0.093547	0.042191	49	0	0.031945	0.213929
Risk_group	Bifidobacterium.reuteri	Risk	0.093547	0.042191	49	0	0.031945	0.213929
LLETZ	Oscillibacter.sp900115635	Yes	0.377501	0.17125	49	0	0.032898	0.220007
LLETZ	Cronobacter.sakazakii	Yes	0.536466	0.243437	49	0	0.032948	0.220036
Previous_PTB	Virgibacillus_G.profundi	Yes	0.388729	0.176661	49	0	0.033197	0.221391
Risk_group	Cutibacterium.granulosum	Risk	-0.22183	0.101145	49	0	0.03375	0.224768
Risk_group	Empedobacter.falsenii	Risk	-0.11198	0.051177	49	0	0.034143	0.227067
Delivery	Lactobacillus.gasseri	PTB	-1.19999	0.549206	49	0	0.034389	0.228393
LLETZ	Odoribacter.splanchnicus	Yes	0.454874	0.209268	49	0	0.035285	0.233745
Risk_group	Coraliomargarita.akajimensis	Risk	-0.17939	0.082533	49	0	0.035292	0.233745
LLETZ	Hungatella.celerecresce	Yes	0.203	0.093	49	0	0.035	0.233

	ns_B		738	775			365	907
LLETZ	Bacillus.halotolerans	Yes	0.151587	0.069935	49	0	0.035777	0.236305
Previous_PTB	Prevotella.copri	Yes	0.103087	0.047715	49	0	0.036353	0.239454
Previous_PTB	Slackia_A.piriformis	Yes	0.103087	0.047715	49	0	0.036353	0.239454
Delivery	Streptococcus.sanguinis	PTB	0.090926	0.04231	49	0	0.037302	0.24537
Previous_PTB	CAG.81.sp900066785	Yes	0.264766	0.123269	49	0	0.037401	0.245681
Previous_PTB	Bacteroides.cellulosilyticus	Yes	0.442547	0.206137	49	0	0.037487	0.24591
Outcome_group	Streptococcus.pyogenes_A	no-risk_PTB	0.356685	0.166424	49	0	0.037794	0.247591
LLETZ	Streptococcus.pneumoniae_C	Yes	0.106814	0.049867	49	0	0.037901	0.247955
LLETZ	Paraprevotella.clara	Yes	0.492458	0.230173	49	0	0.038112	0.248994
Previous_PTB	Peptoniphilus_E.obesi	Yes	0.10318	0.048262	49	0	0.038248	0.249546

Supplementary Table 3 | MaAsLiN analysis of significant associations of gene ontology functions with sample groupings

metadata	feature	value	coef	stderr	pval	qval
**Molecular function**						
Delivery	[MF]receptor activity	PTB	1.0311	0.2733	0.0005	0.1331
Delivery	[MF]ferredoxin NADP reductase activity	PTB	-2.3282	0.6451	0.0008	0.1332
Delivery	[MF]2 3 bisphosphoglycerate independent phosphoglycerate mutase activity	PTB	0.6209	0.2040	0.0040	0.1652
Delivery	[MF]aldose 1 epimerase activity	PTB	0.6846	0.2129	0.0025	0.1652
Delivery	[MF]carbohydrate transmembrane transporter activity	PTB	-1.9152	0.5667	0.0016	0.1652
Delivery	[MF]carboxyl or carbamoyltransferase activity	PTB	0.5346	0.1741	0.0037	0.1652
Delivery	[MF]copper exporting ATPase activity	PTB	1.8530	0.6179	0.0045	0.1652
Delivery	[MF]dihydroneopterin aldolase activity	PTB	0.7876	0.2518	0.0032	0.1652
Delivery	[MF]fatty acid synthase activity	PTB	0.3188	0.1056	0.0043	0.1652
Delivery	[MF]histidine ammonia lyase activity	PTB	0.4058	0.1318	0.0036	0.1652
Delivery	[MF]hydrolase activity acting on carbon nitrogen but not peptide bonds in linear amides	PTB	-1.7480	0.5867	0.0047	0.1652
Delivery	[MF]isochorismate synthase activity	PTB	0.6178	0.1966	0.0030	0.1652
Delivery	[MF]oxidized purine nucleobase lesion DNA N glycosylase activity	PTB	-0.7326	0.2213	0.0019	0.1652
Delivery	[MF]tRNA cytidylyltransferase activity	PTB	-1.7988	0.6007	0.0045	0.1652
Delivery	[MF]oxidoreductase activity acting on NAD P H quinone or similar compound as acceptor	PTB	0.6947	0.2339	0.0049	0.1658
Delivery	[MF]histone acetyltransferase activity	PTB	0.3381	0.1149	0.0052	0.1665
Delivery	[MF]amino acid transmembrane transporter activity	PTB	-0.2386	0.0818	0.0056	0.1671
Delivery	[MF]beta phosphoglucomutase activity	PTB	-0.7542	0.2631	0.0064	0.1671
Delivery	[MF]D lactate dehydrogenase activity	PTB	-0.9749	0.3401	0.0064	0.1671
Delivery	[MF]monosaccharide transporting ATPase activity	PTB	0.3059	0.1056	0.0059	0.1671
Delivery	[MF]organic phosphonate transmembrane transporting ATPase activity	PTB	-1.1617	0.4068	0.0066	0.1671

Delivery	[MF]pantoate beta alanine ligase activity	PTB	0.5836	0.2083	0.0076	0.1719
Delivery	[MF]1 deoxy D xylulose 5 phosphate synthase activity	PTB	-1.5307	0.5548	0.0085	0.1727
Delivery	[MF]NADH dehydrogenase ubiquinone activity	PTB	0.6738	0.2444	0.0085	0.1727
Delivery	[MF]potassium ion transmembrane transporter activity	PTB	-0.6823	0.2466	0.0083	0.1727
Delivery	[MF]GMP reductase activity	PTB	-1.4396	0.5240	0.0087	0.1728
Delivery	[MF]purine nucleobase transmembrane transporter activity	PTB	-0.5632	0.2054	0.0089	0.1728
Delivery	[MF]protein N PI phosphohistidine sugar phosphotransferase activity	PTB	-0.2638	0.0968	0.0093	0.1745
Delivery	[MF]oxo acid lyase activity	PTB	0.6760	0.2493	0.0096	0.1752
Delivery	[MF]thiamine phosphate kinase activity	PTB	0.6114	0.2256	0.0096	0.1752
Delivery	[MF]4 hydroxy 3 methylbut 2 en 1 yl diphosphate synthase activity	PTB	0.7625	0.2873	0.0111	0.1801
Delivery	[MF]deoxyribonucleoside 5 monophosphate N glycosidase activity	PTB	-0.5576	0.2100	0.0111	0.1801
Delivery	[MF]nucleoside deoxyribosyltransferase activity	PTB	-0.5576	0.2100	0.0111	0.1801
Delivery	[MF]transferase activity transferring pentosyl groups	PTB	0.3570	0.1339	0.0108	0.1801
Delivery	[MF]metal ion binding	PTB	0.1058	0.0403	0.0120	0.1816
Delivery	[MF]quinone binding	PTB	0.5979	0.2292	0.0124	0.1823
Delivery	[MF]uracil phosphoribosyltransferase activity	PTB	-0.1699	0.0650	0.0123	0.1823
Delivery	[MF]adenosylmethionine 8 amino 7 oxononanoate transaminase activity	PTB	0.5588	0.2162	0.0132	0.1837
Delivery	[MF]GTP diphosphokinase activity	PTB	0.2749	0.1073	0.0140	0.1837
Delivery	[MF]phosphorelay response regulator activity	PTB	0.3933	0.1517	0.0130	0.1837
Delivery	[MF]ribose 5 phosphate isomerase activity	PTB	-0.1176	0.0463	0.0148	0.1885
Delivery	[MF]catalytic activity	PTB	0.1694	0.0669	0.0151	0.1899
Delivery	[MF]hydrogen ion transmembrane transporter activity	PTB	0.6339	0.2511	0.0154	0.1912
Delivery	[MF]cob I yrinic acid a c diamide adenosyltransferase activity	PTB	0.4868	0.1938	0.0159	0.1933
Delivery	[MF]2 3 bisphosphoglycerate dependent phosphoglycerate mutase activity	PTB	-0.3120	0.1248	0.0163	0.1959

Delivery	[MF]protein transmembrane transporter activity	PTB	0.3915	0.1578	0.0171	0.2016
Delivery	[MF]intramolecular transferase activity phosphotransferases	PTB	1.4728	0.5948	0.0173	0.2028
Delivery	[MF]glycerol 3 phosphate cytidylyltransferase activity	PTB	-0.5993	0.2427	0.0176	0.2038
Delivery	[MF]hydrolase activity acting on acid anhydrides	PTB	-1.4520	0.5919	0.0183	0.2068
Delivery	[MF]magnesium ion transmembrane transporter activity	PTB	0.5479	0.2228	0.0180	0.2068
Delivery	[MF]ATP dependent 5 3 DNA helicase activity	PTB	1.2697	0.5222	0.0193	0.2088
Delivery	[MF]6 phospho beta glucosidase activity	PTB	-0.8775	0.3626	0.0198	0.2113
Delivery	[MF]organic phosphonate transmembrane transporter activity	PTB	-0.8699	0.3594	0.0198	0.2113
Delivery	[MF]2 C methyl D erythritol 2 4 cyclodiphosphate synthase activity	PTB	0.6645	0.2765	0.0207	0.2137
Delivery	[MF]DNA 3 methyladenine glycosylase activity	PTB	-1.3957	0.5862	0.0218	0.2198
Delivery	[MF]phosphatidate cytidylyltransferase activity	PTB	1.1491	0.4825	0.0217	0.2198
Delivery	[MF]5 nucleotidase activity	PTB	0.5104	0.2157	0.0226	0.2230
Delivery	[MF]biotin synthase activity	PTB	0.5042	0.2131	0.0225	0.2230
Delivery	[MF]phosphoribosylglycinamide formyltransferase 2 activity	PTB	0.7532	0.3199	0.0232	0.2263
Delivery	[MF]protein disulfide oxidoreductase activity	PTB	0.2590	0.1101	0.0232	0.2265
Delivery	[MF]phosphoglycerate dehydrogenase activity	PTB	0.7599	0.3238	0.0236	0.2291
Delivery	[MF]starch binding	PTB	0.7298	0.3140	0.0249	0.2324
Delivery	[MF]metallocarboxypeptidase activity	PTB	0.5769	0.2490	0.0253	0.2345
Delivery	[MF]3 dehydroquinate dehydratase activity	PTB	1.3900	0.6009	0.0256	0.2352
Delivery	[MF]phospho N acetylmuramoyl pentapeptide transferase activity	PTB	0.0989	0.0428	0.0256	0.2352
Delivery	[MF]succinate CoA ligase ADP forming activity	PTB	0.4075	0.1788	0.0277	0.2414
Delivery	[MF]cardiolipin synthase activity	PTB	-1.0802	0.4750	0.0280	0.2429
Delivery	[MF]serine type endopeptidase activity	PTB	0.1314	0.0578	0.0282	0.2431
Delivery	[MF]oxygen dependent protoporphyrinogen oxidase activity	PTB	0.4856	0.2148	0.0289	0.2468

Delivery	[MF]undecaprenyl diphosphatase activity	PTB	1.2131	0.5380	0.0293	0.2480
Delivery	[MF]2 C methyl D erythritol 4 phosphate cytidylyltransferase activity	PTB	0.6563	0.2913	0.0294	0.2481
LLETZ	[MF]UDP N acetylmuramoyl L alanyl D glutamyl meso 2 6 diaminopimelyl D alanyl D alanine undecaprenyl phosphate transferase activity	Yes	-0.3495	0.0738	0.0000	0.0991
LLETZ	[MF]pantetheine phosphate adenylyltransferase activity	Yes	-0.8030	0.1969	0.0002	0.1123
LLETZ	[MF]guanylate kinase activity	Yes	-0.4110	0.1138	0.0008	0.1332
LLETZ	[MF]adenine phosphoribosyltransferase activity	Yes	0.5745	0.1674	0.0013	0.1635
LLETZ	[MF]cobalamin binding	Yes	0.8708	0.2917	0.0047	0.1652
LLETZ	[MF]ferric iron binding	Yes	-0.3635	0.1196	0.0040	0.1652
LLETZ	[MF]hydrolase activity hydrolyzing N glycosyl compounds	Yes	-0.8851	0.2904	0.0039	0.1652
LLETZ	[MF]NAD synthase activity	Yes	0.3680	0.1180	0.0032	0.1652
LLETZ	[MF]oxidoreductase activity oxidizing metal ions	Yes	-0.3644	0.1195	0.0039	0.1652
LLETZ	[MF]ribonuclease III activity	Yes	0.4410	0.1472	0.0045	0.1652
LLETZ	[MF]ribosomal large subunit binding	Yes	0.3288	0.1080	0.0040	0.1652
LLETZ	[MF]nicotinate nucleotide adenylyltransferase activity	Yes	-0.2956	0.1031	0.0064	0.1671
LLETZ	[MF]pyridoxal phosphate binding	Yes	-0.2681	0.0936	0.0064	0.1671
LLETZ	[MF]oxidoreductase activity acting on the aldehyde or oxo group of donors NAD or NADP as acceptor	Yes	0.2045	0.0726	0.0073	0.1673
LLETZ	[MF]6 phosphofructokinase activity	Yes	0.6856	0.2486	0.0085	0.1727
LLETZ	[MF]C4 dicarboxylate transmembrane transporter activity	Yes	-0.9360	0.3382	0.0083	0.1727
LLETZ	[MF]glucosamine 6 phosphate deaminase activity	Yes	0.4877	0.1789	0.0092	0.1745
LLETZ	[MF]protein serine threonine tyrosine kinase activity	Yes	1.0974	0.4029	0.0093	0.1745
LLETZ	[MF]mismatched DNA binding	Yes	0.7083	0.2628	0.0100	0.1752
LLETZ	[MF]pseudouridine synthase activity	Yes	0.1789	0.0668	0.0104	0.1784
LLETZ	[MF]5 10 methylenetetrahydrofolate dependent tRNA m5U54 methyltransferase activity	Yes	0.5630	0.2135	0.0116	0.1809

LLETZ	[MF]methylenetetrahydrofolate tRNA uracil 5 methyltransferase FADH2 oxidizing activity	Yes	0.5630	0.2135	0.0116	0.1809
LLETZ	[MF]tRNA adenylyltransferase activity	Yes	0.6771	0.2559	0.0113	0.1809
LLETZ	[MF]putrescine importing ATPase activity	Yes	1.2014	0.4621	0.0127	0.1836
LLETZ	[MF]spermidine importing ATPase activity	Yes	1.2015	0.4621	0.0127	0.1836
LLETZ	[MF]protein methyltransferase activity	Yes	0.4189	0.1631	0.0138	0.1837
LLETZ	[MF]tripeptide aminopeptidase activity	Yes	0.7819	0.3051	0.0140	0.1837
LLETZ	[MF]ADP binding	Yes	0.6052	0.2384	0.0148	0.1885
LLETZ	[MF]methionyl tRNA formyltransferase activity	Yes	0.2857	0.1125	0.0148	0.1885
LLETZ	[MF]phosphoribosyl AMP cyclohydrolase activity	Yes	-0.2837	0.1115	0.0146	0.1885
LLETZ	[MF]thymidine kinase activity	Yes	0.3975	0.1563	0.0147	0.1885
LLETZ	[MF]phosphotransferase activity phosphate group as acceptor	Yes	0.6031	0.2386	0.0152	0.1903
LLETZ	[MF]acyl phosphate glycerol 3 phosphate acyltransferase activity	Yes	0.5787	0.2327	0.0169	0.1997
LLETZ	[MF]tagatose 6 phosphate kinase activity	Yes	1.2006	0.4850	0.0173	0.2028
LLETZ	[MF]aminopeptidase activity	Yes	0.7422	0.3022	0.0182	0.2068
LLETZ	[MF]D alanine poly phosphoribitol ligase activity	Yes	1.2139	0.4967	0.0187	0.2081
LLETZ	[MF]asparagine tRNA ligase activity	Yes	0.6171	0.2541	0.0194	0.2088
LLETZ	[MF]metallopeptidase activity	Yes	0.6630	0.2728	0.0193	0.2088
LLETZ	[MF]sulfurtransferase activity	Yes	0.2492	0.1026	0.0194	0.2088
LLETZ	[MF]2 succinyl 5 enolpyruvyl 6 hydroxy 3 cyclohexene 1 carboxylic acid synthase activity	Yes	-0.9746	0.4040	0.0202	0.2119
LLETZ	[MF]glutamate tRNA ligase activity	Yes	-0.2188	0.0907	0.0202	0.2119
LLETZ	[MF]glycerol 3 phosphate cytidylyltransferase activity	Yes	0.8265	0.3426	0.0202	0.2119
LLETZ	[MF]1 phosphofructokinase activity	Yes	0.4732	0.2050	0.0258	0.2352
LLETZ	[MF]carbon nitrogen ligase activity with glutamine as amido N donor	Yes	0.7002	0.3033	0.0258	0.2352
LLETZ	[MF]D alanyl carrier activity	Yes	1.0650	0.4609	0.0257	0.2352
LLETZ	[MF]thymidylate synthase activity	Yes	0.2401	0.1039	0.0257	0.2352
LLETZ	[MF]dCMP deaminase activity	Yes	0.5722	0.2486	0.0263	0.2368

LLETZ	[MF]Type II site specific deoxyribonuclease activity	Yes	-1.2244	0.5329	0.0265	0.2373
LLETZ	[MF]alpha 1 4 glucosidase activity	Yes	-0.2498	0.1092	0.0271	0.2403
LLETZ	[MF]maltose alpha glucosidase activity	Yes	-0.2498	0.1092	0.0271	0.2403
LLETZ	[MF]3 tRNA processing endoribonuclease activity	Yes	1.0406	0.4577	0.0280	0.2429
LLETZ	[MF]ribonuclease P activity	Yes	0.5615	0.2482	0.0288	0.2468
LLETZ	[MF]serine tRNA ligase activity	Yes	0.3032	0.1348	0.0296	0.2487
Outcome_group	[MF]pyruvate dehydrogenase acetyl transferring activity	no-risk_PTB	1.3623	0.3551	0.0004	0.1331
Outcome_group	[MF]acetylglucosaminyltransferase activity	no-risk_PTB	1.2557	0.3423	0.0007	0.1332
Outcome_group	[MF]voltage gated potassium channel activity	no-risk_PTB	1.9382	0.5387	0.0008	0.1332
Outcome_group	[MF] S 3 amino 2 methylpropionate transaminase activity	no-risk_PTB	2.5220	0.7191	0.0011	0.1499
Outcome_group	[MF]cystathionine gamma synthase activity	no-risk_PTB	2.2987	0.6529	0.0010	0.1499
Outcome_group	[MF]sulfate transmembrane transporting ATPase activity	no-risk_PTB	1.6792	0.4805	0.0011	0.1499
Outcome_group	[MF]6 aminohexanoate cyclic dimer hydrolase activity	no-risk_PTB	1.9404	0.6273	0.0035	0.1652
Outcome_group	[MF]acetoin dehydrogenase activity	no-risk_PTB	1.6547	0.5492	0.0043	0.1652
Outcome_group	[MF]acetyl CoA C acetyltransferase activity	no-risk_PTB	1.2783	0.3963	0.0024	0.1652
Outcome_group	[MF]androstan 3 alpha 17 beta diol dehydrogenase activity	no-risk_PTB	1.5625	0.4887	0.0026	0.1652
Outcome_group	[MF]dihydrolipoyllysine residue succinyltransferase activity	no-risk_PTB	1.9677	0.5922	0.0018	0.1652
Outcome_group	[MF]histidine ammonia lyase activity	no-risk_PTB	1.0607	0.3419	0.0034	0.1652
Outcome_group	[MF]hydro lyase activity	no-risk_PTB	1.5968	0.4980	0.0025	0.1652

Outcome_group	[MF]morphine 6 dehydrogenase activity	no-risk_PTB	1.4763	0.4747	0.0033	0.1652
Outcome_group	[MF]nitric oxide synthase activity	no-risk_PTB	1.5153	0.5057	0.0045	0.1652
Outcome_group	[MF]oxidoreductase activity acting on the CH CH group of donors	no-risk_PTB	1.8028	0.6002	0.0044	0.1652
Outcome_group	[MF]ADP ribose diphosphatase activity	no-risk_PTB	1.3049	0.4594	0.0069	0.1671
Outcome_group	[MF]antioxidant activity	no-risk_PTB	1.7251	0.5962	0.0060	0.1671
Outcome_group	[MF]gamma glutamyl gamma aminobutyrate hydrolase activity	no-risk_PTB	1.5828	0.5542	0.0066	0.1671
Outcome_group	[MF]histone acetyltransferase activity	no-risk_PTB	0.8404	0.2981	0.0072	0.1671
Outcome_group	[MF]hydrolase activity acting on acid anhydrides	no-risk_PTB	4.3338	1.5355	0.0072	0.1671
Outcome_group	[MF]L threonine 3 dehydrogenase activity	no-risk_PTB	1.9384	0.6663	0.0057	0.1671
Outcome_group	[MF]metallocarboxypeptidase activity	no-risk_PTB	1.7823	0.6459	0.0085	0.1727
Outcome_group	[MF]maltose epimerase activity	no-risk_PTB	1.8019	0.6557	0.0087	0.1728
Outcome_group	[MF]inositol monophosphate 1 phosphatase activity	no-risk_PTB	1.3585	0.5040	0.0100	0.1752
Outcome_group	[MF]inositol monophosphate 3 phosphatase activity	no-risk_PTB	1.3585	0.5040	0.0100	0.1752
Outcome_group	[MF]inositol monophosphate 4 phosphatase activity	no-risk_PTB	1.3585	0.5040	0.0100	0.1752
Outcome_group	[MF]aldose 1 epimerase activity	no-risk_PTB	1.4808	0.5524	0.0104	0.1780
Outcome_group	[MF]protein transmembrane transporter activity	no-risk_PTB	1.0737	0.4093	0.0120	0.1816

Outcome_group	[MF]galactonate dehydratase activity	no-risk_PTB	1.2551	0.4790	0.0121	0.1821
Outcome_group	[MF]mannosyl glycoprotein endo beta N acetylglucosaminidase activity	no-risk_PTB	1.5242	0.5841	0.0124	0.1823
Outcome_group	[MF]phosphoglycerate mutase activity	no-risk_PTB	1.8270	0.7108	0.0137	0.1837
Outcome_group	[MF]gluconokinase activity	no-risk_PTB	2.0793	0.8267	0.0157	0.1929
Outcome_group	[MF]oxo acid lyase activity	no-risk_PTB	1.5824	0.6467	0.0186	0.2081
Outcome_group	[MF]pantoate beta alanine ligase activity	no-risk_PTB	1.3003	0.5403	0.0205	0.2128
Outcome_group	[MF]glutamate synthase NADPH activity	no-risk_PTB	1.4494	0.6104	0.0221	0.2217
Outcome_group	[MF]intramolecular lyase activity	no-risk_PTB	2.8218	1.1907	0.0223	0.2230
Outcome_group	[MF]carboxylic ester hydrolase activity	no-risk_PTB	1.1397	0.4904	0.0249	0.2324
Outcome_group	[MF]metalloexopeptidase activity	no-risk_PTB	1.3373	0.5894	0.0284	0.2444
Outcome_group	[MF]2 C methyl D erythritol 4 phosphate cytidylyltransferase activity	no-risk_PTB	1.7074	0.7555	0.0289	0.2468
Outcome_group	[MF]4 hydroxy 3 methylbut 2 en 1 yl diphosphate synthase activity	no-risk_PTB	1.6747	0.7453	0.0298	0.2492
Previous_PTB	[MF]UDP N acetylmuramoyl L alanyl D glutamyl meso 2 6 diaminopimelyl D alanyl D alanine undecaprenyl phosphate transferase activity	Yes	-0.3368	0.0767	0.0001	0.1006
Previous_PTB	[MF]ferric iron binding	Yes	-0.5020	0.1244	0.0002	0.1123
Previous_PTB	[MF]hydrolase activity hydrolyzing N glycosyl compounds	Yes	-1.2145	0.3021	0.0002	0.1123
Previous_PTB	[MF]pantetheine phosphate adenylyltransferase activity	Yes	-0.8603	0.2048	0.0001	0.1123

Previous_PTB	[MF]ribonuclease III activity	Yes	0.6112	0.1531	0.0003	0.1123
Previous_PTB	[MF]oxidoreductase activity oxidizing metal ions	Yes	-0.4888	0.1243	0.0003	0.1144
Previous_PTB	[MF]adenine phosphoribosyltransferase activity	Yes	0.6610	0.1741	0.0005	0.1331
Previous_PTB	[MF]2 succinyl 5 enolpyruvyl 6 hydroxy 3 cyclohexene 1 carboxylic acid synthase activity	Yes	-1.5244	0.4203	0.0008	0.1332
Previous_PTB	[MF]guanylate kinase activity	Yes	-0.4395	0.1184	0.0006	0.1332
Previous_PTB	[MF]NAD synthase activity	Yes	0.4412	0.1227	0.0008	0.1332
Previous_PTB	[MF]ribosomal large subunit binding	Yes	0.4128	0.1124	0.0007	0.1332
Previous_PTB	[MF]C4 dicarboxylate transmembrane transporter activity	Yes	-1.2475	0.3518	0.0010	0.1482
Previous_PTB	[MF]phosphoribosyl AMP cyclohydrolase activity	Yes	-0.3987	0.1160	0.0013	0.1635
Previous_PTB	[MF]Type II site specific deoxyribonuclease activity	Yes	-1.9134	0.5543	0.0013	0.1635
Previous_PTB	[MF]1 4 dihydroxy 2 naphthoate octaprenyltransferase activity	Yes	-1.6545	0.5493	0.0043	0.1652
Previous_PTB	[MF]1 deoxy D xylulose 5 phosphate reductoisomerase activity	Yes	-1.7175	0.5728	0.0045	0.1652
Previous_PTB	[MF]3 isopropylmalate dehydratase activity	Yes	-1.6919	0.5634	0.0044	0.1652
Previous_PTB	[MF]alpha 1 4 glucosidase activity	Yes	-0.3522	0.1136	0.0034	0.1652
Previous_PTB	[MF]D alanine poly phosphoribitol ligase activity	Yes	1.5417	0.5167	0.0047	0.1652
Previous_PTB	[MF]fatty acid synthase activity	Yes	-0.5006	0.1551	0.0024	0.1652
Previous_PTB	[MF]maltose alpha glucosidase activity	Yes	-0.3522	0.1136	0.0034	0.1652
Previous_PTB	[MF]nicotinate nucleotide adenylyltransferase activity	Yes	-0.3403	0.1072	0.0028	0.1652
Previous_PTB	[MF]oxidoreductase activity acting on the aldehyde or oxo group of donors NAD or NADP as acceptor	Yes	0.2267	0.0755	0.0045	0.1652
Previous_PTB	[MF]protein methyltransferase activity	Yes	0.5621	0.1696	0.0019	0.1652
Previous_PTB	[MF]protein serine threonine tyrosine kinase activity	Yes	1.2845	0.4191	0.0038	0.1652
Previous_PTB	[MF]pyridoxal phosphate binding	Yes	-0.3268	0.0973	0.0017	0.1652
Previous_PTB	[MF]rRNA adenine C2 methyltransferase activity	Yes	-1.8586	0.5710	0.0022	0.1652
Previous_PTB	[MF]tRNA adenine C2 methyltransferase activity	Yes	-1.8586	0.5710	0.0022	0.1652
Previous_PTB	[MF]3 isopropylmalate dehydrogenase activity	Yes	-1.4875	0.5041	0.0051	0.1665
Previous_PTB	[MF]cobalamin binding	Yes	0.8894	0.3035	0.0054	0.1665

Previous_PTB	[MF]D alanyl carrier activity	Yes	1.4089	0.4795	0.0053	0.1665
Previous_PTB	[MF]dihydroxy acid dehydratase activity	Yes	-1.6306	0.5534	0.0052	0.1665
Previous_PTB	[MF]leucine tRNA ligase activity	Yes	-1.9199	0.6548	0.0054	0.1665
Previous_PTB	[MF]oxidoreductase activity acting on the CH NH2 group of donors NAD or NADP as acceptor	Yes	-1.9426	0.6602	0.0052	0.1665
Previous_PTB	[MF]putrescine importing ATPase activity	Yes	1.4107	0.4807	0.0053	0.1665
Previous_PTB	[MF]pyridoxal kinase activity	Yes	-1.8902	0.6382	0.0050	0.1665
Previous_PTB	[MF]spermidine importing ATPase activity	Yes	1.4105	0.4807	0.0053	0.1665
Previous_PTB	[MF]sulfurtransferase activity	Yes	0.3158	0.1067	0.0050	0.1665
Previous_PTB	[MF]3 iron 4 sulfur cluster binding	Yes	-1.3532	0.4735	0.0066	0.1671
Previous_PTB	[MF]4 hydroxy 3 methylbut 2 en 1 yl diphosphate reductase activity	Yes	-1.3532	0.4735	0.0066	0.1671
Previous_PTB	[MF]aminoacylase activity	Yes	-0.2673	0.0934	0.0065	0.1671
Previous_PTB	[MF]anthranilate phosphoribosyltransferase activity	Yes	-1.3808	0.4796	0.0062	0.1671
Previous_PTB	[MF]carbon nitrogen ligase activity with glutamine as amido N donor	Yes	0.9186	0.3155	0.0057	0.1671
Previous_PTB	[MF]carbonate dehydratase activity	Yes	-1.4249	0.5033	0.0070	0.1671
Previous_PTB	[MF]deoxyribonuclease IV phage T4 induced activity	Yes	-1.7218	0.6092	0.0071	0.1671
Previous_PTB	[MF]DNA directed DNA polymerase activity	Yes	0.2879	0.1004	0.0064	0.1671
Previous_PTB	[MF]endoribonuclease activity	Yes	0.2135	0.0743	0.0063	0.1671
Previous_PTB	[MF]formate C acetyltransferase activity	Yes	-1.5634	0.5439	0.0063	0.1671
Previous_PTB	[MF]glucosamine 6 phosphate deaminase activity	Yes	0.5287	0.1861	0.0068	0.1671
Previous_PTB	[MF]isocitrate dehydrogenase NADP activity	Yes	-0.9969	0.3430	0.0058	0.1671
Previous_PTB	[MF]ketol acid reductoisomerase activity	Yes	-1.7283	0.6091	0.0069	0.1671
Previous_PTB	[MF]L threonine ammonia lyase activity	Yes	-1.8400	0.6477	0.0069	0.1671
Previous_PTB	[MF]methionyl tRNA formyltransferase activity	Yes	0.3310	0.1171	0.0071	0.1671
Previous_PTB	[MF]N methyltransferase activity	Yes	0.6370	0.2246	0.0069	0.1671
Previous_PTB	[MF]NAD binding	Yes	-0.2429	0.0862	0.0073	0.1671
Previous_PTB	[MF]shikimate kinase activity	Yes	-2.0763	0.7132	0.0057	0.1671
Previous_PTB	[MF]thymidylate synthase activity	Yes	0.3135	0.1081	0.0059	0.1671

Previous_PTB	[MF]tRNA guanine 37 N 1 methyltransferase activity	Yes	0.3449	0.1211	0.0067	0.1671
Previous_PTB	[MF]serine tRNA ligase activity	Yes	0.3928	0.1402	0.0076	0.1719
Previous_PTB	[MF]1 5 phosphoribosyl 5 5 phosphoribosylamino methylideneamino imidazole 4 carboxamide isomerase activity	Yes	-0.7796	0.2787	0.0077	0.1726
Previous_PTB	[MF]dinitrosyl iron complex binding	Yes	-2.2160	0.7955	0.0079	0.1726
Previous_PTB	[MF]protein dimerization activity	Yes	-1.8085	0.6481	0.0078	0.1726
Previous_PTB	[MF]calcium transporting ATPase activity	Yes	-0.3599	0.1296	0.0081	0.1727
Previous_PTB	[MF]imidazoleglycerol phosphate synthase activity	Yes	-1.0720	0.3874	0.0083	0.1727
Previous_PTB	[MF]phosphoribosylanthranilate isomerase activity	Yes	-1.6286	0.5854	0.0080	0.1727
Previous_PTB	[MF]tagatose 6 phosphate kinase activity	Yes	1.3939	0.5045	0.0084	0.1727
Previous_PTB	[MF]5 10 methylenetetrahydrofolate dependent tRNA m5U54 methyltransferase activity	Yes	0.6092	0.2221	0.0089	0.1728
Previous_PTB	[MF]ligase activity forming carbon nitrogen bonds	Yes	0.2566	0.0935	0.0088	0.1728
Previous_PTB	[MF]methylenetetrahydrofolate tRNA uracil 5 methyltransferase FADH2 oxidizing activity	Yes	0.6092	0.2221	0.0089	0.1728
Previous_PTB	[MF]N2 acetyl L ornithine 2 oxoglutarate 5 aminotransferase activity	Yes	-1.6402	0.5986	0.0089	0.1728
Previous_PTB	[MF]thiaminase activity	Yes	-1.3926	0.5063	0.0087	0.1728
Previous_PTB	[MF]tRNA adenylyltransferase activity	Yes	0.7315	0.2662	0.0087	0.1728
Previous_PTB	[MF]glucose 1 phosphate thymidylyltransferase activity	Yes	-1.0741	0.3930	0.0091	0.1738
Previous_PTB	[MF]acetylglutamate kinase activity	Yes	-1.2770	0.4695	0.0094	0.1746
Previous_PTB	[MF]DNA N glycosylase activity	Yes	0.6836	0.2518	0.0095	0.1746
Previous_PTB	[MF]ATP phosphoribosyltransferase activity	Yes	-1.6337	0.6043	0.0098	0.1752
Previous_PTB	[MF]phosphotransferase activity phosphate group as acceptor	Yes	0.6698	0.2482	0.0099	0.1752
Previous_PTB	[MF]argininosuccinate synthase activity	Yes	-1.7961	0.6673	0.0101	0.1760
Previous_PTB	[MF]ADP binding	Yes	0.6667	0.2480	0.0102	0.1765
Previous_PTB	[MF]aminopeptidase activity	Yes	0.8447	0.3143	0.0102	0.1765

Previous_PTB	[MF]acetyl CoA L glutamate N acetyltransferase activity	Yes	-1.6297	0.6144	0.0111	0.1801
Previous_PTB	[MF]glutamate N acetyltransferase activity	Yes	-1.6297	0.6144	0.0111	0.1801
Previous_PTB	[MF]N acetylglucosamine transmembrane transporter activity	Yes	-1.6703	0.6275	0.0109	0.1801
Previous_PTB	[MF]tripeptide aminopeptidase activity	Yes	0.8458	0.3174	0.0108	0.1801
Previous_PTB	[MF]metallopeptidase activity	Yes	0.7521	0.2838	0.0112	0.1805
Previous_PTB	[MF]cystathionine beta lyase activity	Yes	-1.1583	0.4402	0.0118	0.1809
Previous_PTB	[MF]deoxyguanosine kinase activity	Yes	1.3601	0.5177	0.0119	0.1809
Previous_PTB	[MF]glutamate racemase activity	Yes	1.2222	0.4648	0.0118	0.1809
Previous_PTB	[MF]manganese ion transmembrane transporter activity	Yes	-1.3799	0.5231	0.0116	0.1809
Previous_PTB	[MF]mismatched DNA binding	Yes	0.7208	0.2734	0.0116	0.1809
Previous_PTB	[MF]oxidoreductase activity acting on a sulfur group of donors NAD P as acceptor	Yes	-1.5456	0.5883	0.0119	0.1809
Previous_PTB	[MF]phosphate ion binding	Yes	-1.2844	0.4880	0.0117	0.1809
Previous_PTB	[MF]pseudouridine synthase activity	Yes	0.1829	0.0695	0.0117	0.1809
Previous_PTB	[MF]threonine synthase activity	Yes	-1.9621	0.7447	0.0117	0.1809
Previous_PTB	[MF]coproporphyrinogen oxidase activity	Yes	-2.0443	0.7827	0.0123	0.1823
Previous_PTB	[MF]glucose 1 phosphate adenylyltransferase activity	Yes	-2.0299	0.7776	0.0124	0.1823
Previous_PTB	[MF]glycerol 3 phosphate cytidylyltransferase activity	Yes	0.9295	0.3564	0.0125	0.1823
Previous_PTB	[MF]histidinol dehydrogenase activity	Yes	-1.7100	0.6533	0.0122	0.1823
Previous_PTB	[MF]NADPH quinone reductase activity	Yes	0.8788	0.3370	0.0125	0.1823
Previous_PTB	[MF]3 dehydroquinate synthase activity	Yes	-2.2193	0.8567	0.0130	0.1837
Previous_PTB	[MF]alpha L fucosidase activity	Yes	-0.8368	0.3243	0.0134	0.1837
Previous_PTB	[MF]aminoacyl tRNA editing activity	Yes	-0.2788	0.1086	0.0138	0.1837
Previous_PTB	[MF]chorismate synthase activity	Yes	-2.1784	0.8498	0.0139	0.1837
Previous_PTB	[MF]dGTPase activity	Yes	-1.9902	0.7701	0.0132	0.1837
Previous_PTB	[MF]enzyme regulator activity	Yes	-1.1474	0.4464	0.0137	0.1837
Previous_PTB	[MF]fructose bisphosphate aldolase activity	Yes	-2.1528	0.8328	0.0132	0.1837
Previous_PTB	[MF]protein glutamine N methyltransferase activity	Yes	-1.8627	0.7232	0.0135	0.1837

Previous_PTB	[MF]ribonuclease P activity	Yes	0.6634	0.2582	0.0137	0.1837
Previous_PTB	[MF]sedoheptulose 7 phosphate D glyceraldehyde 3 phosphate glyceronetransferase activity	Yes	-1.1811	0.4581	0.0134	0.1837
Previous_PTB	[MF]single stranded DNA endodeoxyribonuclease activity	Yes	-0.9900	0.3815	0.0129	0.1837
Previous_PTB	[MF]tRNA nucleotidyltransferase activity	Yes	-1.3537	0.5254	0.0135	0.1837
Previous_PTB	[MF]tRNA specific ribonuclease activity	Yes	-1.3537	0.5254	0.0135	0.1837
Previous_PTB	[MF]UDP N acetylmuramate L alanine ligase activity	Yes	0.3541	0.1370	0.0132	0.1837
Previous_PTB	[MF]thymidine kinase activity	Yes	0.4155	0.1626	0.0142	0.1852
Previous_PTB	[MF]exoribonuclease activity producing 5 phosphomonoesters	Yes	-1.3823	0.5439	0.0147	0.1885
Previous_PTB	[MF]imidazoleglycerol phosphate dehydratase activity	Yes	-1.4450	0.5692	0.0148	0.1885
Previous_PTB	[MF]UDP N acetyl D glucosamine N acetylmuramoyl L alanyl D glutamyl meso 2 6 diaminopimelyl D alanyl D alanine diphosphoundecaprenol 4 beta N acetylglucosaminlytransferase activity	Yes	0.2998	0.1183	0.0150	0.1896
Previous_PTB	[MF]undecaprenyldiphospho muramoylpentapeptide beta N acetylglucosaminyltransferase activity	Yes	0.2998	0.1183	0.0150	0.1896
Previous_PTB	[MF]exodeoxyribonuclease VII activity	Yes	0.2710	0.1071	0.0151	0.1899
Previous_PTB	[MF]UDP N acetylmuramoylalanyl D glutamyl 2 6 diaminopimelate D alanyl D alanine ligase activity	Yes	0.2498	0.0988	0.0152	0.1903
Previous_PTB	[MF]6 phosphofructokinase activity	Yes	0.6508	0.2586	0.0157	0.1929
Previous_PTB	[MF]penicillin binding	Yes	1.1874	0.4722	0.0157	0.1929
Previous_PTB	[MF]phosphoglucomutase activity	Yes	-2.1446	0.8528	0.0157	0.1929
Previous_PTB	[MF]tryptophan synthase activity	Yes	-1.6991	0.6759	0.0158	0.1929
Previous_PTB	[MF]hydroxymethylglutaryl CoA synthase activity	Yes	0.7918	0.3156	0.0159	0.1939
Previous_PTB	[MF]cysteine synthase activity	Yes	-1.6492	0.6590	0.0162	0.1954
Previous_PTB	[MF]glutamate tRNA ligase activity	Yes	-0.2363	0.0944	0.0162	0.1954
Previous_PTB	[MF]tyrosine based site specific recombinase activity	Yes	0.4027	0.1611	0.0164	0.1959
Previous_PTB	[MF]pyridoxal 5 phosphate synthase glutamine hydrolysing activity	Yes	-1.8618	0.7483	0.0168	0.1996

Previous_PTB	[MF]uracil DNA N glycosylase activity	Yes	1.2790	0.5173	0.0174	0.2038
Previous_PTB	[MF]L isoleucine transaminase activity	Yes	-1.5534	0.6332	0.0183	0.2068
Previous_PTB	[MF]L leucine transaminase activity	Yes	-1.5534	0.6332	0.0183	0.2068
Previous_PTB	[MF]L valine transaminase activity	Yes	-1.5534	0.6332	0.0183	0.2068
Previous_PTB	[MF]peroxiredoxin activity	Yes	-1.6932	0.6898	0.0182	0.2068
Previous_PTB	[MF]hydroxyethylthiazole kinase activity	Yes	-1.8922	0.7719	0.0184	0.2072
Previous_PTB	[MF]serine O acetyltransferase activity	Yes	-1.7896	0.7332	0.0188	0.2084
Previous_PTB	[MF]5 3 exoribonuclease activity	Yes	1.6427	0.6743	0.0191	0.2088
Previous_PTB	[MF]5 carboxyamino imidazole ribonucleotide mutase activity	Yes	-1.7723	0.7278	0.0191	0.2088
Previous_PTB	[MF]5 carboxyamino imidazole ribonucleotide synthase activity	Yes	-1.6736	0.6868	0.0190	0.2088
Previous_PTB	[MF]aspartate kinase activity	Yes	-2.5118	1.0338	0.0194	0.2088
Previous_PTB	[MF]thymidylate kinase activity	Yes	1.0134	0.4186	0.0198	0.2113
Previous_PTB	[MF]3 deoxy 7 phosphoheptulonate synthase activity	Yes	-2.2299	0.9248	0.0202	0.2119
Previous_PTB	[MF]histidinol phosphate transaminase activity	Yes	-2.1192	0.8785	0.0202	0.2119
Previous_PTB	[MF]transferase activity transferring amino acyl groups	Yes	-1.4676	0.6077	0.0201	0.2119
Previous_PTB	[MF]acetolactate synthase activity	Yes	-1.8041	0.7534	0.0211	0.2166
Previous_PTB	[MF]amidophosphoribosyltransferase activity	Yes	-1.7522	0.7329	0.0213	0.2178
Previous_PTB	[MF]acyl phosphate glycerol 3 phosphate acyltransferase activity	Yes	0.5767	0.2421	0.0217	0.2198
Previous_PTB	[MF]coenzyme binding	Yes	0.1675	0.0703	0.0216	0.2198
Previous_PTB	[MF]UDP N acetylmuramoyl tripeptide D alanyl D alanine ligase activity	Yes	0.2361	0.0990	0.0216	0.2198
Previous_PTB	[MF]transferase activity transferring hexosyl groups	Yes	-1.8901	0.7968	0.0222	0.2223
Previous_PTB	[MF]3 oxoacyl acyl carrier protein reductase NADPH activity	Yes	-2.0717	0.8752	0.0225	0.2230
Previous_PTB	[MF]glutamate 5 semialdehyde dehydrogenase activity	Yes	-1.7832	0.7544	0.0227	0.2236
Previous_PTB	[MF]peptide transporter activity	Yes	1.4469	0.6140	0.0231	0.2263
Previous_PTB	[MF]deoxyadenosine kinase activity	Yes	1.0516	0.4482	0.0236	0.2291

Previous_PTB	[MF]ribose 5 phosphate isomerase activity	Yes	-0.1593	0.0680	0.0238	0.2293
Previous_PTB	[MF]phosphoribosylaminoimidazolesuccinocarboxamide synthase activity	Yes	-1.7991	0.7678	0.0238	0.2294
Previous_PTB	[MF]IMP cyclohydrolase activity	Yes	-1.9794	0.8482	0.0244	0.2318
Previous_PTB	[MF]phosphoribosylaminoimidazolecarboxamide formyltransferase activity	Yes	-1.9794	0.8482	0.0244	0.2318
Previous_PTB	[MF]sodium ion transmembrane transporter activity	Yes	-1.4888	0.6373	0.0242	0.2318
Previous_PTB	[MF]argininosuccinate lyase activity	Yes	-1.9023	0.8168	0.0246	0.2319
Previous_PTB	[MF]dTDP 4 dehydrorhamnose 3 5 epimerase activity	Yes	-1.5132	0.6495	0.0246	0.2319
Previous_PTB	[MF]2 3 4 5 tetrahydropyridine 2 6 dicarboxylate N succinyltransferase activity	Yes	-1.4475	0.6227	0.0249	0.2324
Previous_PTB	[MF]rRNA cytosine N4 methyltransferase activity	Yes	-0.1998	0.0861	0.0251	0.2333
Previous_PTB	[MF]L phenylalanine 2 oxoglutarate aminotransferase activity	Yes	-2.0886	0.9016	0.0254	0.2345
Previous_PTB	[MF]glucose 6 phosphate dehydrogenase activity	Yes	0.3382	0.1470	0.0264	0.2368
Previous_PTB	[MF]tetrahydrofolylpolyglutamate synthase activity	Yes	1.0875	0.4728	0.0263	0.2368
Previous_PTB	[MF]N acetyl gamma glutamyl phosphate reductase activity	Yes	-2.1352	0.9287	0.0264	0.2370
Previous_PTB	[MF]phosphomevalonate kinase activity	Yes	1.7425	0.7602	0.0269	0.2398
Previous_PTB	[MF]4 hydroxy tetrahydrodipicolinate synthase	Yes	-2.0091	0.8791	0.0273	0.2403
Previous_PTB	[MF]ferrous iron transmembrane transporter activity	Yes	-1.1128	0.4870	0.0273	0.2403
Previous_PTB	[MF]succinyl diaminopimelate desuccinylase activity	Yes	-1.4336	0.6273	0.0273	0.2403
Previous_PTB	[MF]phosphorelay sensor kinase activity	Yes	0.1669	0.0732	0.0277	0.2414
Previous_PTB	[MF]phosphoribosylformylglycinamidine cyclo ligase activity	Yes	-1.9864	0.8713	0.0276	0.2414
Previous_PTB	[MF]amidase activity	Yes	1.4995	0.6583	0.0278	0.2417
Previous_PTB	[MF]dCMP deaminase activity	Yes	0.5844	0.2586	0.0289	0.2468
Previous_PTB	[MF]phosphoribosylamine glycine ligase activity	Yes	-2.1732	0.9620	0.0290	0.2469
Previous_PTB	[MF]phosphoribosylaminoimidazole carboxylase activity	Yes	-2.0045	0.8882	0.0292	0.2476
Previous_PTB	[MF]S ribosylhomocysteine lyase activity	Yes	-1.6315	0.7237	0.0293	0.2480

Previous_PTB	[MF]nicotinate nucleotide diphosphorylase carboxylating activity	Yes	-0.2552	0.1134	0.0296	0.2487
Risk_group	[MF]UDP N acetylmuramoyl L alanyl D glutamyl meso 2 6 diaminopimelyl D alanyl D alanine undecaprenyl phosphate transferase activity	Risk	0.3651	0.0830	0.0001	0.1006
Risk_group	[MF]hydrolase activity hydrolyzing N glycosyl compounds	Risk	1.3274	0.3269	0.0002	0.1123
Risk_group	[MF]pantetheine phosphate adenylyltransferase activity	Risk	0.8795	0.2216	0.0003	0.1123
Risk_group	[MF]C4 dicarboxylate transmembrane transporter activity	Risk	1.4286	0.3807	0.0005	0.1331
Risk_group	[MF]ferric iron binding	Risk	0.5124	0.1346	0.0004	0.1331
Risk_group	[MF]oxidoreductase activity oxidizing metal ions	Risk	0.5028	0.1345	0.0005	0.1331
Risk_group	[MF]2 succinyl 5 enolpyruvyl 6 hydroxy 3 cyclohexene 1 carboxylic acid synthase activity	Risk	1.6531	0.4548	0.0007	0.1332
Risk_group	[MF]threonine synthase activity	Risk	2.8189	0.8058	0.0011	0.1499
Risk_group	[MF]3 dehydroquinate synthase activity	Risk	2.9333	0.9270	0.0029	0.1652
Risk_group	[MF]3 deoxy 7 phosphoheptulonate synthase activity	Risk	3.1471	1.0007	0.0030	0.1652
Risk_group	[MF]5 carboxyamino imidazole ribonucleotide synthase activity	Risk	2.2673	0.7432	0.0039	0.1652
Risk_group	[MF]amidophosphoribosyltransferase activity	Risk	2.3673	0.7930	0.0047	0.1652
Risk_group	[MF]argininosuccinate lyase activity	Risk	2.7091	0.8838	0.0038	0.1652
Risk_group	[MF]argininosuccinate synthase activity	Risk	2.3875	0.7221	0.0019	0.1652
Risk_group	[MF]carbonate dehydratase activity	Risk	1.7574	0.5446	0.0024	0.1652
Risk_group	[MF]chorismate synthase activity	Risk	3.0240	0.9195	0.0020	0.1652
Risk_group	[MF]coproporphyrinogen oxidase activity	Risk	2.5362	0.8469	0.0045	0.1652
Risk_group	[MF]crossover junction endodeoxyribonuclease activity	Risk	2.4662	0.7721	0.0026	0.1652
Risk_group	[MF]cysteine synthase activity	Risk	2.1803	0.7131	0.0038	0.1652
Risk_group	[MF]dGTPase activity	Risk	2.5615	0.8333	0.0037	0.1652
Risk_group	[MF]dinitrosyl iron complex binding	Risk	2.5701	0.8608	0.0047	0.1652

Risk_group	[MF]fatty acid synthase activity	Risk	0.5065	0.1678	0.0043	0.1652
Risk_group	[MF]formate C acetyltransferase activity	Risk	1.7699	0.5885	0.0044	0.1652
Risk_group	[MF]glucose 1 phosphate thymidylyltransferase activity	Risk	1.2684	0.4252	0.0047	0.1652
Risk_group	[MF]glutamate 5 semialdehyde dehydrogenase activity	Risk	2.5240	0.8163	0.0035	0.1652
Risk_group	[MF]guanylate kinase activity	Risk	0.4373	0.1281	0.0014	0.1652
Risk_group	[MF]histidinol phosphate transaminase activity	Risk	2.9065	0.9506	0.0038	0.1652
Risk_group	[MF]hydroxyethylthiazole kinase activity	Risk	2.5949	0.8353	0.0033	0.1652
Risk_group	[MF]L isoleucine transaminase activity	Risk	2.1240	0.6852	0.0034	0.1652
Risk_group	[MF]L leucine transaminase activity	Risk	2.1240	0.6852	0.0034	0.1652
Risk_group	[MF]L valine transaminase activity	Risk	2.1240	0.6852	0.0034	0.1652
Risk_group	[MF]leucine tRNA ligase activity	Risk	2.3567	0.7085	0.0018	0.1652
Risk_group	[MF]nicotinate nucleotide adenylyltransferase activity	Risk	0.3857	0.1160	0.0018	0.1652
Risk_group	[MF]phosphate ion binding	Risk	1.6105	0.5280	0.0039	0.1652
Risk_group	[MF]phosphoglucomutase activity	Risk	2.8437	0.9228	0.0036	0.1652
Risk_group	[MF]phosphoribosyl AMP cyclohydrolase activity	Risk	0.3738	0.1255	0.0048	0.1652
Risk_group	[MF]phosphoribosylaminoimidazole carboxylase activity	Risk	3.0266	0.9611	0.0030	0.1652
Risk_group	[MF]phosphoribosylaminoimidazolesuccinocarboxamide synthase activity	Risk	2.5049	0.8308	0.0043	0.1652
Risk_group	[MF]phosphoribosylformylglycinamidine cyclo ligase activity	Risk	2.9415	0.9427	0.0032	0.1652
Risk_group	[MF]protein methyltransferase activity	Risk	-0.5513	0.1835	0.0044	0.1652
Risk_group	[MF]pyridoxal 5 phosphate synthase glutamine hydrolysing activity	Risk	2.4703	0.8097	0.0039	0.1652
Risk_group	[MF]ribonuclease III activity	Risk	-0.5631	0.1657	0.0015	0.1652
Risk_group	[MF]rRNA adenine C2 methyltransferase activity	Risk	1.8450	0.6179	0.0046	0.1652
Risk_group	[MF]sedoheptulose 7 phosphate D glyceraldehyde 3 phosphate glyceronetransferase activity	Risk	1.5449	0.4957	0.0033	0.1652
Risk_group	[MF]serine O acetyltransferase activity	Risk	2.3646	0.7933	0.0047	0.1652
Risk_group	[MF]shikimate kinase activity	Risk	2.4807	0.7717	0.0025	0.1652

Risk_group	[MF]sodium ion transmembrane transporter activity	Risk	2.0641	0.6896	0.0046	0.1652
Risk_group	[MF]tRNA adenine C2 methyltransferase activity	Risk	1.8450	0.6179	0.0046	0.1652
Risk_group	[MF]tRNA nucleotidyltransferase activity	Risk	1.7182	0.5685	0.0042	0.1652
Risk_group	[MF]tRNA specific ribonuclease activity	Risk	1.7182	0.5685	0.0042	0.1652
Risk_group	[MF]Type II site specific deoxyribonuclease activity	Risk	1.8363	0.5998	0.0038	0.1652
Risk_group	[MF]5 carboxyamino imidazole ribonucleotide mutase activity	Risk	2.3407	0.7876	0.0048	0.1658
Risk_group	[MF]protein glutamine N methyltransferase activity	Risk	2.3107	0.7826	0.0051	0.1665
Risk_group	[MF]single stranded DNA endodeoxyribonuclease activity	Risk	1.2198	0.4128	0.0051	0.1665
Risk_group	[MF]1 deoxy D xylulose 5 phosphate reductoisomerase activity	Risk	1.7623	0.6198	0.0068	0.1671
Risk_group	[MF]3 oxoacyl acyl carrier protein reductase NADPH activity	Risk	2.6836	0.9470	0.0070	0.1671
Risk_group	[MF]adenine phosphoribosyltransferase activity	Risk	-0.5454	0.1884	0.0059	0.1671
Risk_group	[MF]dTDP 4 dehydrorhamnose 3 5 epimerase activity	Risk	2.0246	0.7028	0.0062	0.1671
Risk_group	[MF]glucose 1 phosphate adenylyltransferase activity	Risk	2.3956	0.8413	0.0067	0.1671
Risk_group	[MF]IMP cyclohydrolase activity	Risk	2.6137	0.9178	0.0067	0.1671
Risk_group	[MF]L phenylalanine 2 oxoglutarate aminotransferase activity	Risk	2.8154	0.9756	0.0061	0.1671
Risk_group	[MF]oxidoreductase activity acting on the aldehyde or oxo group of donors NAD or NADP as acceptor	Risk	-0.2308	0.0817	0.0072	0.1671
Risk_group	[MF]oxidoreductase activity acting on the CH NH2 group of donors NAD or NADP as acceptor	Risk	2.0891	0.7144	0.0055	0.1671
Risk_group	[MF]phosphoribosylaminoimidazolecarboxamide formyltransferase activity	Risk	2.6137	0.9178	0.0067	0.1671
Risk_group	[MF]protein dimerization activity	Risk	1.9959	0.7013	0.0068	0.1671
Risk_group	[MF]ribosomal large subunit binding	Risk	-0.3442	0.1216	0.0070	0.1671
Risk_group	[MF]tryptophan synthase activity	Risk	2.1310	0.7314	0.0056	0.1671
Risk_group	[MF]3 iron 4 sulfur cluster binding	Risk	1.4282	0.5124	0.0079	0.1726
Risk_group	[MF]3 isopropylmalate dehydratase activity	Risk	1.7017	0.6097	0.0078	0.1726

Risk_group	[MF]4 hydroxy 3 methylbut 2 en 1 yl diphosphate reductase activity	Risk	1.4282	0.5124	0.0079	0.1726
Risk_group	[MF]fructose bisphosphate aldolase activity	Risk	2.5088	0.9011	0.0079	0.1726
Risk_group	[MF]pyridoxal phosphate binding	Risk	0.2943	0.1053	0.0077	0.1726
Risk_group	[MF]anthranilate phosphoribosyltransferase activity	Risk	1.4311	0.5190	0.0085	0.1727
Risk_group	[MF]carbon nitrogen ligase activity with glutamine as amido N donor	Risk	-0.9445	0.3414	0.0083	0.1727
Risk_group	[MF]phosphoribosylanthranilate isomerase activity	Risk	1.7482	0.6335	0.0085	0.1727
Risk_group	[MF]thiaminase activity	Risk	1.5187	0.5479	0.0082	0.1727
Risk_group	[MF]1 4 dihydroxy 2 naphthoate octaprenyltransferase activity	Risk	1.6232	0.5944	0.0091	0.1738
Risk_group	[MF]dihydroxy acid dehydratase activity	Risk	1.6361	0.5988	0.0091	0.1738
Risk_group	[MF]isocitrate dehydrogenase NADP activity	Risk	1.0155	0.3711	0.0090	0.1738
Risk_group	[MF]3 isopropylmalate dehydrogenase activity	Risk	1.4820	0.5454	0.0095	0.1746
Risk_group	[MF]ketol acid reductoisomerase activity	Risk	1.7898	0.6591	0.0095	0.1746
Risk_group	[MF]metallopeptidase activity	Risk	-0.8342	0.3071	0.0095	0.1746
Risk_group	[MF]protein serine threonine tyrosine kinase activity	Risk	-1.2228	0.4535	0.0100	0.1752
Risk_group	[MF]thiamine phosphate diphosphorylase activity	Risk	2.2050	0.8166	0.0099	0.1752
Risk_group	[MF]transferase activity transferring amino acyl groups	Risk	1.7758	0.6576	0.0099	0.1752
Risk_group	[MF]D alanine poly phosphoribitol ligase activity	Risk	-1.4988	0.5591	0.0104	0.1780
Risk_group	[MF]acetylglutamate kinase activity	Risk	1.3511	0.5080	0.0110	0.1801
Risk_group	[MF]alpha 1 4 glucosidase activity	Risk	0.3282	0.1229	0.0106	0.1801
Risk_group	[MF]ATP phosphoribosyltransferase activity	Risk	1.7391	0.6539	0.0109	0.1801
Risk_group	[MF]maltose alpha glucosidase activity	Risk	0.3282	0.1229	0.0106	0.1801
Risk_group	[MF]N2 acetyl L ornithine 2 oxoglutarate 5 aminotransferase activity	Risk	1.7256	0.6477	0.0108	0.1801
Risk_group	[MF]NAD binding	Risk	0.2481	0.0932	0.0109	0.1801
Risk_group	[MF]alpha L fucosidase activity	Risk	0.9252	0.3509	0.0116	0.1809
Risk_group	[MF]oxidoreductase activity acting on a sulfur group of donors NAD P as acceptor	Risk	1.6827	0.6366	0.0114	0.1809

Risk_group	[MF]peptide transporter activity	Risk	-1.7493	0.6644	0.0117	0.1809
Risk_group	[MF]deoxyribonuclease IV phage T4 induced activity	Risk	1.7187	0.6592	0.0125	0.1823
Risk_group	[MF]L threonine ammonia lyase activity	Risk	1.8227	0.7009	0.0127	0.1836
Risk_group	[MF]1 5 phosphoribosyl 5 5 phosphoribosylamino methylideneamino imidazole 4 carboxamide isomerase activity	Risk	0.7725	0.3016	0.0140	0.1837
Risk_group	[MF]acetyl CoA L glutamate N acetyltransferase activity	Risk	1.7150	0.6648	0.0134	0.1837
Risk_group	[MF]amidase activity	Risk	-1.8237	0.7123	0.0140	0.1837
Risk_group	[MF]glutamate N acetyltransferase activity	Risk	1.7150	0.6648	0.0134	0.1837
Risk_group	[MF]glutamate tRNA ligase activity	Risk	0.2644	0.1021	0.0131	0.1837
Risk_group	[MF]histidinol dehydrogenase activity	Risk	1.8141	0.7069	0.0138	0.1837
Risk_group	[MF]imidazoleglycerol phosphate synthase activity	Risk	1.0834	0.4192	0.0132	0.1837
Risk_group	[MF]NAD synthase activity	Risk	-0.3440	0.1328	0.0131	0.1837
Risk_group	[MF]putrescine importing ATPase activity	Risk	-1.3373	0.5201	0.0137	0.1837
Risk_group	[MF]spermidine importing ATPase activity	Risk	-1.3374	0.5201	0.0137	0.1837
Risk_group	[MF]calcium transporting ATPase activity	Risk	0.3569	0.1402	0.0146	0.1885
Risk_group	[MF]D alanyl carrier activity	Risk	-1.3063	0.5188	0.0156	0.1929
Risk_group	[MF]tripeptide aminopeptidase activity	Risk	-0.8611	0.3434	0.0160	0.1942
Risk_group	[MF]tRNA adenylyltransferase activity	Risk	-0.7193	0.2880	0.0164	0.1962
Risk_group	[MF]glutamate racemase activity	Risk	-1.2524	0.5029	0.0167	0.1991
Risk_group	[MF]succinyl diaminopimelate desuccinylase activity	Risk	1.6814	0.6787	0.0172	0.2028
Risk_group	[MF]exoribonuclease activity producing 5 phosphomonoesters	Risk	1.4533	0.5885	0.0176	0.2038
Risk_group	[MF]tagatose 6 phosphate kinase activity	Risk	-1.3479	0.5459	0.0176	0.2038
Risk_group	[MF]acetolactate synthase activity	Risk	2.0064	0.8153	0.0179	0.2068
Risk_group	[MF]N acetylglucosamine transmembrane transporter activity	Risk	1.6692	0.6790	0.0181	0.2068
Risk_group	[MF]ribonuclease M5 activity	Risk	-1.3261	0.5413	0.0184	0.2072
Risk_group	[MF]imidazoleglycerol phosphate dehydratase activity	Risk	1.5051	0.6159	0.0187	0.2081
Risk_group	[MF]N methyltransferase activity	Risk	-0.5939	0.2430	0.0187	0.2081

Risk_group	[MF]cysteine type endopeptidase activity	Risk	-1.2245	0.5013	0.0188	0.2081
Risk_group	[MF]2 3 4 5 tetrahydropyridine 2 6 dicarboxylate N succinyltransferase activity	Risk	1.6399	0.6738	0.0192	0.2088
Risk_group	[MF]cystathionine beta lyase activity	Risk	1.1576	0.4763	0.0193	0.2088
Risk_group	[MF]nicotinate nucleotide diphosphorylase carboxylating activity	Risk	0.2987	0.1227	0.0192	0.2088
Risk_group	[MF]aminoacylase activity	Risk	0.2438	0.1011	0.0202	0.2119
Risk_group	[MF]rRNA cytosine N4 methyltransferase activity	Risk	0.2245	0.0931	0.0203	0.2119
Risk_group	[MF]6 phosphofructokinase activity	Risk	-0.6741	0.2798	0.0204	0.2122
Risk_group	[MF]peroxiredoxin activity	Risk	1.7941	0.7464	0.0206	0.2137
Risk_group	[MF]manganese ion transmembrane transporter activity	Risk	1.3580	0.5660	0.0208	0.2150
Risk_group	[MF]5 3 exoribonuclease activity	Risk	-1.7468	0.7296	0.0211	0.2166
Risk_group	[MF]sodium dicarboxylate symporter activity	Risk	-1.0317	0.4318	0.0213	0.2180
Risk_group	[MF]5 10 methylenetetrahydrofolate dependent tRNA m5U54 methyltransferase activity	Risk	-0.5710	0.2403	0.0220	0.2215
Risk_group	[MF]methylenetetrahydrofolate tRNA uracil 5 methyltransferase FADH2 oxidizing activity	Risk	-0.5710	0.2403	0.0220	0.2215
Risk_group	[MF]indole 3 glycerol phosphate synthase activity	Risk	1.2477	0.5274	0.0226	0.2230
Risk_group	[MF]uracil DNA N glycosylase activity	Risk	-1.3205	0.5597	0.0229	0.2256
Risk_group	[MF]transferase activity transferring acyl groups other than amino acyl groups	Risk	-0.2457	0.1043	0.0232	0.2263
Risk_group	[MF]aminoacyl tRNA editing activity	Risk	0.2756	0.1175	0.0237	0.2293
Risk_group	[MF]CDP glycerol glycerophosphotransferase activity	Risk	-1.6021	0.6867	0.0244	0.2318
Risk_group	[MF]DNA N glycosylase activity	Risk	-0.6358	0.2725	0.0244	0.2318
Risk_group	[MF]lysozyme activity	Risk	-0.9180	0.3933	0.0243	0.2318
Risk_group	[MF]aminoacyl tRNA ligase activity	Risk	-0.7924	0.3400	0.0246	0.2319
Risk_group	[MF]peptidyl prolyl cis trans isomerase activity	Risk	0.2464	0.1058	0.0246	0.2319
Risk_group	[MF]L threonylcarbamoyladenylate synthase	Risk	-1.1009	0.4730	0.0247	0.2322
Risk_group	[MF]succinate dehydrogenase activity	Risk	-1.7440	0.7528	0.0254	0.2345
Risk_group	[MF]phosphoribosylamine glycine ligase activity	Risk	2.4039	1.0410	0.0258	0.2352

Risk_group	[MF]enzyme regulator activity	Risk	1.1120	0.4830	0.0262	0.2368
Risk_group	[MF]homoserine kinase activity	Risk	2.1293	0.9250	0.0262	0.2368
Risk_group	[MF]transferase activity transferring hexosyl groups	Risk	1.9836	0.8622	0.0263	0.2368
Risk_group	[MF]malate dehydrogenase decarboxylating NAD activity	Risk	1.4561	0.6357	0.0270	0.2402
Risk_group	[MF]sulfurtransferase activity	Risk	-0.2642	0.1155	0.0271	0.2403
Risk_group	[MF]polyamine binding	Risk	-1.5592	0.6830	0.0274	0.2409
Risk_group	[MF]aspartate ammonia ligase activity	Risk	-1.3607	0.5986	0.0281	0.2429
Risk_group	[MF]deoxyguanosine kinase activity	Risk	-1.2685	0.5602	0.0287	0.2464
Risk_group	[MF]ADP binding	Risk	-0.6040	0.2683	0.0295	0.2487
Risk_group	[MF]glucosamine 6 phosphate deaminase activity	Risk	-0.4524	0.2014	0.0298	0.2492
Risk_group	[MF]rRNA methyltransferase activity	Risk	-1.6545	0.7365	0.0299	0.2492
**Biological process**						
Delivery	[BP]respiratory electron transport chain	PTB	0.9109	0.2131	0.0001	0.1243
Delivery	[BP]alginic acid biosynthetic process	PTB	0.4421	0.1508	0.0054	0.1809
Delivery	[BP]AMP salvage	PTB	-0.3240	0.0887	0.0007	0.1809
Delivery	[BP]ATP hydrolysis coupled proton transport	PTB	0.1730	0.0500	0.0012	0.1809
Delivery	[BP]folic acid biosynthetic process	PTB	0.8304	0.2748	0.0042	0.1809
Delivery	[BP]glucose catabolic process	PTB	0.6332	0.2053	0.0036	0.1809
Delivery	[BP]pentose phosphate shunt non oxidative branch	PTB	-0.1468	0.0488	0.0044	0.1809
Delivery	[BP]peptidyl L beta methylthioaspartic acid biosynthetic process from peptidyl aspartic acid	PTB	0.4434	0.1405	0.0029	0.1809
Delivery	[BP]protein tetramerization	PTB	-1.2026	0.3659	0.0020	0.1809
Delivery	[BP]RNA modification	PTB	0.5130	0.1707	0.0044	0.1809
Delivery	[BP]RNA repair	PTB	-1.8043	0.6032	0.0046	0.1809
Delivery	[BP]sporulation resulting in formation of a cellular spore	PTB	0.7151	0.2155	0.0019	0.1809
Delivery	[BP]ATP synthesis coupled electron transport	PTB	0.6788	0.2336	0.0058	0.1906
Delivery	[BP]1 deoxy D xylulose 5 phosphate biosynthetic process	PTB	-1.5067	0.5510	0.0090	0.1909

Delivery	[BP]ATP synthesis coupled proton transport	PTB	0.5726	0.2007	0.0066	0.1909
Delivery	[BP]cellular response to stress	PTB	0.4363	0.1543	0.0071	0.1909
Delivery	[BP]deoxyribonucleoside monophosphate catabolic process	PTB	-0.5483	0.2068	0.0112	0.1909
Delivery	[BP]DNA unwinding involved in DNA replication	PTB	1.5541	0.5866	0.0112	0.1909
Delivery	[BP]histidine catabolic process to glutamate and formamide	PTB	0.4948	0.1762	0.0075	0.1909
Delivery	[BP]histidine catabolic process to glutamate and formate	PTB	0.4948	0.1762	0.0075	0.1909
Delivery	[BP]intein mediated protein splicing	PTB	0.3442	0.1245	0.0084	0.1909
Delivery	[BP]misfolded or incompletely synthesized protein catabolic process	PTB	0.4180	0.1572	0.0109	0.1909
Delivery	[BP]purine nucleotide metabolic process	PTB	-0.1421	0.0527	0.0100	0.1909
Delivery	[BP]tetrapyrrole biosynthetic process	PTB	0.5733	0.2123	0.0099	0.1909
Delivery	[BP]pyridoxine biosynthetic process	PTB	0.5672	0.2196	0.0133	0.2043
Delivery	[BP]peptidoglycan turnover	PTB	0.3930	0.1534	0.0140	0.2060
Delivery	[BP]S adenosylmethionine biosynthetic process	PTB	0.1682	0.0655	0.0138	0.2060
Delivery	[BP]regulation of sporulation	PTB	-0.1571	0.0622	0.0153	0.2080
Delivery	[BP]protein transport by the Tat complex	PTB	0.4000	0.1590	0.0157	0.2110
Delivery	[BP]lactose metabolic process	PTB	-0.8066	0.3219	0.0161	0.2130
Delivery	[BP]teichoic acid biosynthetic process	PTB	-0.5893	0.2380	0.0173	0.2144
Delivery	[BP]fumarate metabolic process	PTB	-1.4314	0.5863	0.0188	0.2150
Delivery	[BP]toxin biosynthetic process	PTB	-0.6149	0.2523	0.0190	0.2150
Delivery	[BP]tRNA processing	PTB	0.1303	0.0534	0.0189	0.2150
Delivery	[BP]rRNA 5 end processing	PTB	-0.5042	0.2081	0.0197	0.2168
Delivery	[BP]iron ion transport	PTB	0.3872	0.1600	0.0198	0.2171
Delivery	[BP]biotin biosynthetic process	PTB	0.6279	0.2619	0.0209	0.2218
Delivery	[BP]iron ion homeostasis	PTB	0.4557	0.1898	0.0207	0.2218
Delivery	[BP]electron transport chain	PTB	0.6624	0.2860	0.0254	0.2404
Delivery	[BP]thiamine diphosphate biosynthetic process	PTB	0.1764	0.0774	0.0277	0.2490
Delivery	[BP]cellular metabolic process	PTB	0.6600	0.2905	0.0282	0.2493

LLETZ	[BP]adenine salvage	Yes	0.5696	0.1609	0.0010	0.1809
LLETZ	[BP]ATP hydrolysis coupled proton transport	Yes	-0.2391	0.0706	0.0015	0.1809
LLETZ	[BP]cellular iron ion homeostasis	Yes	-0.3595	0.1115	0.0024	0.1809
LLETZ	[BP]glucuronate catabolic process	Yes	-0.6367	0.2111	0.0043	0.1809
LLETZ	[BP]regulation of DNA repair	Yes	0.2939	0.0896	0.0021	0.1809
LLETZ	[BP]barrier septum assembly	Yes	0.2598	0.0980	0.0112	0.1909
LLETZ	[BP]DNA biosynthetic process	Yes	0.4012	0.1515	0.0113	0.1909
LLETZ	[BP]fructose 6 phosphate metabolic process	Yes	0.6806	0.2426	0.0075	0.1909
LLETZ	[BP]FtsZ dependent cytokinesis	Yes	0.2980	0.1060	0.0074	0.1909
LLETZ	[BP]mismatch repair	Yes	0.7032	0.2549	0.0085	0.1909
LLETZ	[BP]mRNA catabolic process	Yes	0.4943	0.1759	0.0074	0.1909
LLETZ	[BP]mRNA processing	Yes	0.3891	0.1419	0.0088	0.1909
LLETZ	[BP]regulation of carbohydrate metabolic process	Yes	1.0905	0.3972	0.0088	0.1909
LLETZ	[BP]response to redox state	Yes	0.6904	0.2557	0.0099	0.1909
LLETZ	[BP]rRNA catabolic process	Yes	0.3895	0.1408	0.0083	0.1909
LLETZ	[BP]SOS response	Yes	0.1104	0.0413	0.0106	0.1909
LLETZ	[BP]tRNA thio modification	Yes	0.6758	0.2511	0.0101	0.1909
LLETZ	[BP]protein dephosphorylation	Yes	0.6000	0.2330	0.0135	0.2053
LLETZ	[BP]ATP synthesis coupled proton transport	Yes	-0.7232	0.2834	0.0143	0.2060
LLETZ	[BP]peptide metabolic process	Yes	0.7825	0.3083	0.0148	0.2067
LLETZ	[BP]chromosome segregation	Yes	0.2397	0.0946	0.0151	0.2080
LLETZ	[BP]D gluconate metabolic process	Yes	0.6848	0.2731	0.0160	0.2130
LLETZ	[BP]D tagatose 6 phosphate catabolic process	Yes	1.1914	0.4794	0.0169	0.2141
LLETZ	[BP]dTTP biosynthetic process	Yes	0.2410	0.0971	0.0171	0.2144
LLETZ	[BP]asparaginyl tRNA aminoacylation	Yes	0.6120	0.2484	0.0178	0.2150
LLETZ	[BP]DNA catabolic process	Yes	0.3007	0.1233	0.0189	0.2150
LLETZ	[BP]dTMP biosynthetic process	Yes	0.2370	0.0963	0.0179	0.2150
LLETZ	[BP]enterobactin biosynthetic process	Yes	-0.8863	0.3608	0.0182	0.2150
LLETZ	[BP]lipoteichoic acid biosynthetic process	Yes	1.2089	0.4952	0.0188	0.2150
LLETZ	[BP]regulation of translational termination	Yes	0.6025	0.2449	0.0180	0.2150

LLETZ	[BP]teichoic acid biosynthetic process	Yes	0.8173	0.3360	0.0192	0.2150
LLETZ	[BP]lactose metabolic process	Yes	1.0899	0.4544	0.0209	0.2218
LLETZ	[BP]rRNA base methylation	Yes	-0.2190	0.0911	0.0205	0.2218
LLETZ	[BP]toxin biosynthetic process	Yes	0.8571	0.3562	0.0205	0.2218
LLETZ	[BP]tRNA 5 leader removal	Yes	0.6212	0.2614	0.0220	0.2289
LLETZ	[BP]selenocysteine biosynthetic process	Yes	0.2988	0.1271	0.0234	0.2355
LLETZ	[BP]selenocysteinyl tRNA Sec biosynthetic process	Yes	0.2988	0.1271	0.0234	0.2355
LLETZ	[BP]seryl tRNA aminoacylation	Yes	0.2986	0.1271	0.0235	0.2355
LLETZ	[BP]protein polymerization	Yes	0.3718	0.1587	0.0238	0.2367
LLETZ	[BP]DNA modification	Yes	1.0823	0.4661	0.0250	0.2402
LLETZ	[BP]purine nucleotide metabolic process	Yes	0.1722	0.0744	0.0255	0.2404
LLETZ	[BP]cell morphogenesis	Yes	0.2247	0.0976	0.0262	0.2441
LLETZ	[BP]RNA catabolic process	Yes	0.4919	0.2152	0.0272	0.2483
LLETZ	[BP]pyrimidine nucleotide metabolic process	Yes	0.5212	0.2299	0.0284	0.2493
LLETZ	[BP]tRNA modification	Yes	0.2522	0.1112	0.0283	0.2493
Outcome_group	[BP]acetoin catabolic process	no-risk_PTB	1.5942	0.5395	0.0051	0.1809
Outcome_group	[BP]polyphosphate metabolic process	no-risk_PTB	1.5008	0.4170	0.0008	0.1809
Outcome_group	[BP]protein processing	no-risk_PTB	1.9272	0.6379	0.0042	0.1809
Outcome_group	[BP]regulation of protein stability	no-risk_PTB	1.4226	0.5062	0.0074	0.1909
Outcome_group	[BP]protein transport by the Tat complex	no-risk_PTB	1.0483	0.4124	0.0147	0.2060
Outcome_group	[BP]alginic acid biosynthetic process	no-risk_PTB	0.9673	0.3912	0.0174	0.2150
Outcome_group	[BP]nucleotide salvage	no-risk_PTB	1.6248	0.6698	0.0195	0.2164
Outcome_group	[BP]D ribose catabolic process	no-risk_PTB	2.7768	1.1672	0.0219	0.2283

Previous_PTB	[BP]cellular iron ion homeostasis	Yes	-0.4977	0.1160	0.0001	0.1243
Previous_PTB	[BP]adenine salvage	Yes	0.6553	0.1674	0.0003	0.1392
Previous_PTB	[BP]dTTP biosynthetic process	Yes	0.3923	0.1010	0.0004	0.1392
Previous_PTB	[BP]enterobactin biosynthetic process	Yes	-1.4144	0.3754	0.0005	0.1521
Previous_PTB	[BP]FtsZ dependent cytokinesis	Yes	0.4144	0.1103	0.0005	0.1521
Previous_PTB	[BP]ATP hydrolysis coupled proton transport	Yes	-0.2607	0.0734	0.0009	0.1809
Previous_PTB	[BP]barrier septum assembly	Yes	0.3243	0.1020	0.0027	0.1809
Previous_PTB	[BP]branched chain amino acid biosynthetic process	Yes	-1.7570	0.5741	0.0038	0.1809
Previous_PTB	[BP]cell cycle	Yes	0.2405	0.0782	0.0036	0.1809
Previous_PTB	[BP]chromosome segregation	Yes	0.3327	0.0984	0.0016	0.1809
Previous_PTB	[BP]DNA catabolic process	Yes	0.4042	0.1283	0.0030	0.1809
Previous_PTB	[BP]dTMP biosynthetic process	Yes	0.2990	0.1001	0.0047	0.1809
Previous_PTB	[BP]leucyl tRNA aminoacylation	Yes	-1.9093	0.6478	0.0052	0.1809
Previous_PTB	[BP]lipoteichoic acid biosynthetic process	Yes	1.5375	0.5151	0.0047	0.1809
Previous_PTB	[BP]mRNA processing	Yes	0.4798	0.1476	0.0022	0.1809
Previous_PTB	[BP]proteolysis	Yes	-1.3037	0.4218	0.0035	0.1809
Previous_PTB	[BP]pyridoxal 5 phosphate salvage	Yes	-1.8951	0.6435	0.0052	0.1809
Previous_PTB	[BP]regulation of carbohydrate metabolic process	Yes	1.2717	0.4132	0.0036	0.1809
Previous_PTB	[BP]regulation of cell shape	Yes	0.2208	0.0697	0.0028	0.1809
Previous_PTB	[BP]rRNA base methylation	Yes	-0.3342	0.0947	0.0010	0.1809
Previous_PTB	[BP]rRNA catabolic process	Yes	0.4911	0.1465	0.0017	0.1809
Previous_PTB	[BP]selenocysteine biosynthetic process	Yes	0.3892	0.1322	0.0052	0.1809
Previous_PTB	[BP]selenocysteinyl tRNA Sec biosynthetic process	Yes	0.3892	0.1322	0.0052	0.1809
Previous_PTB	[BP]seryl tRNA aminoacylation	Yes	0.3875	0.1322	0.0054	0.1809
Previous_PTB	[BP]threonine catabolic process	Yes	-1.6016	0.5452	0.0053	0.1809
Previous_PTB	[BP]tRNA modification	Yes	0.3413	0.1156	0.0051	0.1809
Previous_PTB	[BP]amino acid transport	Yes	-1.6736	0.5886	0.0068	0.1909
Previous_PTB	[BP]branched chain amino acid metabolic process	Yes	-1.4346	0.5348	0.0103	0.1909
Previous_PTB	[BP]carbon utilization	Yes	-1.4011	0.5153	0.0094	0.1909
Previous_PTB	[BP]cell division	Yes	0.1895	0.0701	0.0098	0.1909

Previous_PTB	[BP]cellular amino acid catabolic process	Yes	-1.4472	0.5430	0.0108	0.1909
Previous_PTB	[BP]D gluconate metabolic process	Yes	0.7945	0.2841	0.0077	0.1909
Previous_PTB	[BP]D tagatose 6 phosphate catabolic process	Yes	1.3846	0.4986	0.0081	0.1909
Previous_PTB	[BP]dimethylallyl diphosphate biosynthetic process	Yes	-1.5814	0.5761	0.0088	0.1909
Previous_PTB	[BP]DNA biosynthetic process	Yes	0.4179	0.1576	0.0111	0.1909
Previous_PTB	[BP]GTP metabolic process	Yes	-2.0219	0.7652	0.0114	0.1909
Previous_PTB	[BP]IMP biosynthetic process	Yes	-0.8607	0.3230	0.0108	0.1909
Previous_PTB	[BP]iron sulfur cluster assembly	Yes	1.1702	0.4341	0.0100	0.1909
Previous_PTB	[BP]isocitrate metabolic process	Yes	-0.8433	0.3010	0.0076	0.1909
Previous_PTB	[BP]isopentenyl diphosphate biosynthetic process methylerythritol 4 phosphate pathway	Yes	-2.2932	0.7979	0.0063	0.1909
Previous_PTB	[BP]lactose metabolic process	Yes	1.2484	0.4727	0.0115	0.1909
Previous_PTB	[BP]leucine biosynthetic process	Yes	-1.6920	0.6147	0.0086	0.1909
Previous_PTB	[BP]lipid glycosylation	Yes	0.2913	0.1076	0.0097	0.1909
Previous_PTB	[BP]mismatch repair	Yes	0.7134	0.2652	0.0101	0.1909
Previous_PTB	[BP]mRNA catabolic process	Yes	0.5259	0.1830	0.0063	0.1909
Previous_PTB	[BP]negative regulation of transcription DNA templated	Yes	0.1977	0.0689	0.0063	0.1909
Previous_PTB	[BP]peptide metabolic process	Yes	0.8813	0.3207	0.0087	0.1909
Previous_PTB	[BP]protein dephosphorylation	Yes	0.6615	0.2424	0.0092	0.1909
Previous_PTB	[BP]regulation of DNA repair	Yes	0.2664	0.0932	0.0065	0.1909
Previous_PTB	[BP]response to redox state	Yes	0.7450	0.2660	0.0076	0.1909
Previous_PTB	[BP]teichoic acid biosynthetic process	Yes	0.9202	0.3495	0.0117	0.1909
Previous_PTB	[BP]terpenoid biosynthetic process	Yes	-2.3145	0.8521	0.0095	0.1909
Previous_PTB	[BP]toxin biosynthetic process	Yes	1.0141	0.3705	0.0090	0.1909
Previous_PTB	[BP]tRNA 5 leader removal	Yes	0.7294	0.2720	0.0103	0.1909
Previous_PTB	[BP]tRNA thio modification	Yes	0.7202	0.2612	0.0085	0.1909
Previous_PTB	[BP]valine biosynthetic process	Yes	-1.8507	0.6851	0.0098	0.1909
Previous_PTB	[BP]phosphatidylinositol phosphorylation	Yes	-2.0224	0.7691	0.0118	0.1913
Previous_PTB	[BP]phosphate containing compound metabolic	Yes	-1.9288	0.7371	0.0122	0.1962

	process					
Previous_PTB	[BP]proline biosynthetic process	Yes	-1.2920	0.4968	0.0127	0.2017
Previous_PTB	[BP]DNA methylation	Yes	0.7192	0.2788	0.0134	0.2043
Previous_PTB	[BP]peptidyl glutamine methylation	Yes	-1.8655	0.7227	0.0133	0.2043
Previous_PTB	[BP]protein targeting	Yes	0.4046	0.1568	0.0134	0.2043
Previous_PTB	[BP]protein polymerization	Yes	0.4228	0.1651	0.0140	0.2060
Previous_PTB	[BP]regulation of nitrogen utilization	Yes	-1.1399	0.4449	0.0140	0.2060
Previous_PTB	[BP]tryptophan biosynthetic process	Yes	-2.0012	0.7801	0.0139	0.2060
Previous_PTB	[BP]fructose 6 phosphate metabolic process	Yes	0.6453	0.2524	0.0142	0.2060
Previous_PTB	[BP]glutamate biosynthetic process	Yes	-1.5374	0.6039	0.0146	0.2060
Previous_PTB	[BP]modification dependent protein catabolic process	Yes	-1.2090	0.4756	0.0147	0.2060
Previous_PTB	[BP]glucose metabolic process	Yes	0.2692	0.1067	0.0154	0.2080
Previous_PTB	[BP]establishment of competence for transformation	Yes	1.1080	0.4425	0.0161	0.2130
Previous_PTB	[BP]pathogenesis	Yes	1.3252	0.5306	0.0164	0.2141
Previous_PTB	[BP]porphyrin containing compound biosynthetic process	Yes	-1.5628	0.6284	0.0168	0.2141
Previous_PTB	[BP]proteasomal protein catabolic process	Yes	-1.3215	0.5304	0.0167	0.2141
Previous_PTB	[BP]response to stress	Yes	-0.1685	0.0678	0.0168	0.2141
Previous_PTB	[BP]vitamin B6 biosynthetic process	Yes	-1.8674	0.7539	0.0173	0.2144
Previous_PTB	[BP]cell wall organization	Yes	0.1542	0.0631	0.0187	0.2150
Previous_PTB	[BP]cellular amino acid metabolic process	Yes	-0.1653	0.0672	0.0180	0.2150
Previous_PTB	[BP]dUMP biosynthetic process	Yes	-1.8463	0.7575	0.0190	0.2150
Previous_PTB	[BP]glycogen biosynthetic process	Yes	-2.0207	0.8233	0.0182	0.2150
Previous_PTB	[BP]purine nucleotide metabolic process	Yes	0.1892	0.0774	0.0186	0.2150
Previous_PTB	[BP]pyridoxal phosphate biosynthetic process	Yes	-2.0157	0.8232	0.0185	0.2150
Previous_PTB	[BP]glucuronate catabolic process	Yes	-0.5335	0.2196	0.0194	0.2157
Previous_PTB	[BP]cell morphogenesis	Yes	0.2453	0.1015	0.0200	0.2183
Previous_PTB	[BP]DNA dependent DNA replication	Yes	1.2815	0.5336	0.0207	0.2218
Previous_PTB	[BP]primary metabolic process	Yes	0.8664	0.3618	0.0211	0.2218
Previous_PTB	[BP]dTDP biosynthetic process	Yes	1.0443	0.4373	0.0214	0.2243

Previous_PTB	[BP]carbohydrate transport	Yes	-2.5026	1.0545	0.0222	0.2294
Previous_PTB	[BP]extracellular polysaccharide biosynthetic process	Yes	-1.2546	0.5293	0.0223	0.2301
Previous_PTB	[BP]positive regulation of transcription DNA templated	Yes	-1.5989	0.6770	0.0228	0.2338
Previous_PTB	[BP]regulation of pH	Yes	-1.4884	0.6353	0.0238	0.2367
Previous_PTB	[BP]arginine biosynthetic process via ornithine	Yes	-1.8856	0.8071	0.0242	0.2380
Previous_PTB	[BP]DNA modification	Yes	1.1328	0.4848	0.0242	0.2380
Previous_PTB	[BP]ATP synthesis coupled proton transport	Yes	-0.6816	0.2948	0.0256	0.2409
Previous_PTB	[BP]threonine biosynthetic process	Yes	-1.9412	0.8448	0.0265	0.2448
Previous_PTB	[BP]tRNA threonylcarbamoyladenosine modification	Yes	0.3505	0.1524	0.0264	0.2448
Previous_PTB	[BP]positive regulation of translation	Yes	-2.2586	0.9845	0.0267	0.2455
Previous_PTB	[BP]isoleucine biosynthetic process	Yes	-2.1341	0.9333	0.0272	0.2483
Previous_PTB	[BP]rRNA methylation	Yes	0.8210	0.3596	0.0274	0.2490
Previous_PTB	[BP]DNA unwinding involved in DNA replication	Yes	1.9593	0.8614	0.0280	0.2493
Previous_PTB	[BP]regulation of translational termination	Yes	0.5787	0.2548	0.0282	0.2493
Risk_group	[BP]cellular iron ion homeostasis	Risk	0.4973	0.1255	0.0003	0.1392
Risk_group	[BP]enterobactin biosynthetic process	Risk	1.5886	0.4062	0.0003	0.1392
Risk_group	[BP]adenine salvage	Risk	-0.5520	0.1811	0.0039	0.1809
Risk_group	[BP]arginine biosynthetic process via ornithine	Risk	2.6751	0.8733	0.0038	0.1809
Risk_group	[BP]ATP hydrolysis coupled proton transport	Risk	0.2629	0.0795	0.0019	0.1809
Risk_group	[BP]carbon utilization	Risk	1.7300	0.5576	0.0034	0.1809
Risk_group	[BP]cellular amino acid catabolic process	Risk	1.8369	0.5875	0.0032	0.1809
Risk_group	[BP]dTTP biosynthetic process	Risk	-0.3341	0.1093	0.0038	0.1809
Risk_group	[BP]dUMP biosynthetic process	Risk	2.4142	0.8196	0.0052	0.1809
Risk_group	[BP]FtsZ dependent cytokinesis	Risk	-0.3871	0.1193	0.0023	0.1809
Risk_group	[BP]glucuronate catabolic process	Risk	0.7547	0.2377	0.0028	0.1809
Risk_group	[BP]GTP metabolic process	Risk	2.5083	0.8280	0.0041	0.1809
Risk_group	[BP]L proline biosynthetic process	Risk	2.6888	0.9163	0.0053	0.1809
Risk_group	[BP]leucine biosynthetic process	Risk	2.1045	0.6652	0.0029	0.1809
Risk_group	[BP]leucyl tRNA aminoacylation	Risk	2.3341	0.7009	0.0018	0.1809

Risk_group	[BP]peptidyl glutamine methylation	Risk	2.3094	0.7820	0.0051	0.1809
Risk_group	[BP]phosphate containing compound metabolic process	Risk	2.4545	0.7976	0.0036	0.1809
Risk_group	[BP]phosphatidylinositol phosphorylation	Risk	2.8220	0.8322	0.0015	0.1809
Risk_group	[BP]porphyrin containing compound biosynthetic process	Risk	2.1125	0.6800	0.0033	0.1809
Risk_group	[BP]positive regulation of transcription DNA templated	Risk	2.1514	0.7325	0.0053	0.1809
Risk_group	[BP]pyridoxal phosphate biosynthetic process	Risk	2.7442	0.8908	0.0036	0.1809
Risk_group	[BP]regulation of pH	Risk	2.0576	0.6874	0.0046	0.1809
Risk_group	[BP]rRNA base methylation	Risk	0.3400	0.1025	0.0019	0.1809
Risk_group	[BP]valine biosynthetic process	Risk	2.3328	0.7413	0.0030	0.1809
Risk_group	[BP]vitamin B6 biosynthetic process	Risk	2.4856	0.8158	0.0039	0.1809
Risk_group	[BP]amino acid transport	Risk	1.7043	0.6369	0.0105	0.1909
Risk_group	[BP]branched chain amino acid biosynthetic process	Risk	1.6925	0.6212	0.0093	0.1909
Risk_group	[BP]branched chain amino acid metabolic process	Risk	1.5539	0.5787	0.0103	0.1909
Risk_group	[BP]cysteine biosynthetic process from serine	Risk	2.0424	0.7572	0.0099	0.1909
Risk_group	[BP]dimethylallyl diphosphate biosynthetic process	Risk	1.7232	0.6233	0.0084	0.1909
Risk_group	[BP]establishment of competence for transformation	Risk	-1.2927	0.4788	0.0099	0.1909
Risk_group	[BP]glutamate biosynthetic process	Risk	1.7215	0.6534	0.0117	0.1909
Risk_group	[BP]glycogen biosynthetic process	Risk	2.4962	0.8908	0.0076	0.1909
Risk_group	[BP]histidine biosynthetic process	Risk	0.8959	0.3270	0.0089	0.1909
Risk_group	[BP]IMP biosynthetic process	Risk	0.9589	0.3495	0.0088	0.1909
Risk_group	[BP]isopentenyl diphosphate biosynthetic process methylerythritol 4 phosphate pathway	Risk	2.2918	0.8634	0.0111	0.1909
Risk_group	[BP]lipoteichoic acid biosynthetic process	Risk	-1.5061	0.5574	0.0098	0.1909
Risk_group	[BP]mRNA catabolic process	Risk	-0.5238	0.1980	0.0114	0.1909
Risk_group	[BP]positive regulation of translation	Risk	2.9867	1.0652	0.0075	0.1909
Risk_group	[BP]proline biosynthetic process	Risk	1.4834	0.5376	0.0085	0.1909
Risk_group	[BP]proteolysis	Risk	1.3090	0.4565	0.0064	0.1909
Risk_group	[BP]regulation of carbohydrate metabolic process	Risk	-1.2217	0.4471	0.0091	0.1909

Risk_group	[BP]response to redox state	Risk	-0.7579	0.2878	0.0117	0.1909
Risk_group	[BP]threonine catabolic process	Risk	1.5691	0.5899	0.0109	0.1909
Risk_group	[BP]tryptophan biosynthetic process	Risk	2.2818	0.8441	0.0098	0.1909
Risk_group	[BP]peptide metabolic process	Risk	-0.9063	0.3470	0.0124	0.1973
Risk_group	[BP]isocitrate metabolic process	Risk	0.8451	0.3256	0.0129	0.2030
Risk_group	[BP]chromosome segregation	Risk	-0.2760	0.1065	0.0130	0.2036
Risk_group	[BP]DNA methylation	Risk	-0.7668	0.3017	0.0147	0.2060
Risk_group	[BP]response to stress	Risk	0.1873	0.0733	0.0142	0.2060
Risk_group	[BP]tRNA thio modification	Risk	-0.7196	0.2826	0.0145	0.2060
Risk_group	[BP]regulation of cell shape	Risk	-0.1903	0.0754	0.0153	0.2080
Risk_group	[BP]toxin biosynthetic process	Risk	-1.0130	0.4009	0.0153	0.2080
Risk_group	[BP]D tagatose 6 phosphate catabolic process	Risk	-1.3504	0.5396	0.0162	0.2130
Risk_group	[BP]D gluconate metabolic process	Risk	-0.7665	0.3074	0.0166	0.2141
Risk_group	[BP]fructose 6 phosphate metabolic process	Risk	-0.6805	0.2731	0.0166	0.2141
Risk_group	[BP]barrier septum assembly	Risk	-0.2736	0.1103	0.0171	0.2144
Risk_group	[BP] de novo AMP biosynthetic process	Risk	-0.1648	0.0669	0.0178	0.2150
Risk_group	[BP]modification dependent protein catabolic process	Risk	1.2533	0.5147	0.0191	0.2150
Risk_group	[BP]proteasomal protein catabolic process	Risk	1.3959	0.5739	0.0192	0.2150
Risk_group	[BP]purine nucleobase biosynthetic process	Risk	2.5489	1.0442	0.0188	0.2150
Risk_group	[BP]pathogenesis	Risk	-1.3755	0.5741	0.0210	0.2218
Risk_group	[BP]sodium ion transport	Risk	-1.5148	0.6419	0.0229	0.2338
Risk_group	[BP]protein polymerization	Risk	-0.4209	0.1786	0.0231	0.2347
Risk_group	[BP]lactose metabolic process	Risk	-1.1979	0.5115	0.0239	0.2367
Risk_group	[BP]DNA biosynthetic process	Risk	-0.3969	0.1705	0.0247	0.2401
Risk_group	[BP]protein dephosphorylation	Risk	-0.6103	0.2623	0.0247	0.2401
Risk_group	[BP]selenocysteine biosynthetic process	Risk	-0.3326	0.1431	0.0249	0.2401
Risk_group	[BP]selenocysteinyl tRNA Sec biosynthetic process	Risk	-0.3326	0.1431	0.0249	0.2401
Risk_group	[BP]tRNA modification	Risk	-0.2908	0.1251	0.0249	0.2401
Risk_group	[BP]septin ring assembly	Risk	-1.7383	0.7492	0.0251	0.2403
Risk_group	[BP]seryl tRNA aminoacylation	Risk	-0.3317	0.1431	0.0253	0.2404

Risk_group	[BP]cell wall macromolecule catabolic process	Risk	-0.9346	0.4057	0.0261	0.2441
Risk_group	[BP]regulation of nitrogen utilization	Risk	1.1059	0.4814	0.0265	0.2448
Risk_group	[BP]polyamine transport	Risk	-1.5578	0.6837	0.0277	0.2490
Risk_group	[BP]primary metabolic process	Risk	-0.8922	0.3915	0.0277	0.2490
Risk_group	[BP]L asparagine biosynthetic process	Risk	-1.3695	0.6042	0.0285	0.2493
Risk_group	[BP]threonine biosynthetic process	Risk	2.0741	0.9141	0.0283	0.2493
**Cell cycle functions**						
Delivery	[CC]ATP binding cassette ABC transporter complex	PTB	-0.1960	0.0645	0.0040	0.2414
Delivery	[CC]cell outer membrane	PTB	0.7627	0.3222	0.0225	0.2414
Delivery	[CC]GMP reductase complex	PTB	-1.4567	0.5281	0.0085	0.2414
Delivery	[CC]proton transporting ATP synthase complex coupling factor F o	PTB	0.1966	0.0830	0.0224	0.2414
Delivery	[CC]proton transporting two sector ATPase complex catalytic domain	PTB	0.7260	0.2362	0.0037	0.2414
Delivery	[CC]TAT protein transport complex	PTB	0.3981	0.1634	0.0190	0.2414
Delivery	[CC]tricarboxylic acid cycle enzyme complex	PTB	-1.4512	0.5878	0.0176	0.2414
LLETZ	[CC]cell division site	Yes	0.5439	0.2085	0.0124	0.2414
LLETZ	[CC]exodeoxyribonuclease VII complex	Yes	0.3106	0.1283	0.0198	0.2414
LLETZ	[CC]Holliday junction helicase complex	Yes	0.1997	0.0835	0.0212	0.2414
Outcome_group	[CC]TAT protein transport complex	no-risk_PTB	1.1453	0.4238	0.0098	0.2414
Outcome_group	[CC]voltage gated potassium channel complex	no-risk_PTB	1.9737	0.5502	0.0008	0.2414
Previous_PTB	[CC]3 isopropylmalate dehydratase complex	Yes	-1.2840	0.4625	0.0081	0.2414
Previous_PTB	[CC]cell division site	Yes	0.6188	0.2169	0.0066	0.2414
Previous_PTB	[CC]exodeoxyribonuclease VII complex	Yes	0.4042	0.1335	0.0041	0.2414
Previous_PTB	[CC]fatty acid synthase complex	Yes	-1.3555	0.4567	0.0049	0.2414
Previous_PTB	[CC]Holliday junction helicase complex	Yes	0.2259	0.0869	0.0127	0.2414
Previous_PTB	[CC]organelle inner membrane	Yes	-1.6757	0.6350	0.0115	0.2414
Previous_PTB	[CC]primosome complex	Yes	0.5271	0.2187	0.0203	0.2414

Previous_PTB	[CC]proteasome complex	Yes	-1.1385	0.4277	0.0109	0.2414
Previous_PTB	[CC]protein complex	Yes	0.4424	0.1817	0.0191	0.2414
Previous_PTB	[CC]tRNA m1A methyltransferase complex	Yes	-0.9715	0.3637	0.0106	0.2414
Risk_group	[CC]3 isopropylmalate dehydratase complex	Risk	1.2707	0.5004	0.0148	0.2414
Risk_group	[CC]cell division site	Risk	-0.6586	0.2346	0.0075	0.2414
Risk_group	[CC]cell wall	Risk	-1.8267	0.7422	0.0179	0.2414
Risk_group	[CC]extracellular region	Risk	-0.5341	0.2166	0.0177	0.2414
Risk_group	[CC]fatty acid synthase complex	Risk	1.3523	0.4941	0.0090	0.2414
Risk_group	[CC]organelle inner membrane	Risk	1.6735	0.6871	0.0191	0.2414
Risk_group	[CC]proteasome complex	Risk	1.1282	0.4628	0.0190	0.2414
Risk_group	[CC]septin ring	Risk	-1.7743	0.7527	0.0230	0.2414
Risk_group	[CC]tRNA m1A methyltransferase complex	Risk	1.1807	0.3936	0.0045	0.2414

Supplementary Table 4 | Microbial sequencing reads from studies using shotgun metagenomics on vaginal samples

**Sample size**	**Microbial reads**	**Platform**	**Author**	**Year**	**Journal**	**PMID**
101	1,020,000	HiSeq	Aliaga Goltsman *et al*	2018	Genome Research	30232199
47	100,000	NextSeq	Lev-Sagie *et al*	2019	Nature Medicine	31591599
555	100,000	HiSeq	Fettweis *et al*	2019	Nature Medicine	31142849
19	1,070,000 (median)	HiSeq	Ferretti *et al*	2018	Cell Host & Microbe	30001516
40	39,261	HiSeq	Leiby *et al*	2018	Microbiome	30376898
54	208,225	NextSeq	Oliver *et al*	2019	bioRxiv	https://doi.org/10.1101/840520

Supplementary Table 5 | Top 50 taxonomic composition of control samples

Taxon	Water1	Water2
unclassified	5.46	27.97
Cutibacterium acnes	2.58	16.04
Actinomyces sp000195595	0	1.73
Gordonia jacobaea	0.01	0.83
Rothia dentocariosa	0.02	0.77
Staphylococcus epidermidis	0.07	0.71
Cutibacterium namnetense	0.01	0.65
Pseudomonas_E putida	0	0.6
Bifidobacterium animalis	0.02	0.48
Peptoniphilus_A harei_A	0	0.48
Cutibacterium humerusii	0.09	0.46
Corynebacterium kefirresidentii	0.07	0.42
Acinetobacter johnsonii	0.01	0.42
Prevotella disiens	0	0.42
Rothia mucilaginosa	0	0.42
Finegoldia magna_H	0.01	0.4
Lawsonella clevelandensis_A	0	0.4
Actinomyces oris_A	0	0.33
Pseudomonas_E sp001320045	0	0.33
Streptococcus gordonii	0	0.33
Lactobacillus gasseri	0.02	0.31
Stenotrophomonas bentonitica	0.01	0.31
Micrococcus luteus	0.01	0.29
Rothia sp001808955	0	0.29
Kocuria rhizophila_B	0	0.27
Escherichia flexneri	0.37	0.25
Xanthomonas campestris	0	0.25
Cutibacterium granulosum	0.02	0.23
Prevotella corporis	0	0.23
Lactococcus lactis_E	0	0.21
Varibaculum sp900106855	0	0.21
Corynebacterium otitidis	0.02	0.19
Diaphorobacter sp003248855	0.01	0.19
Pseudomonas_E putida_H	0	0.19
Streptococcus oralis	0	0.19
Anaerococcus sp900258475	0	0.17
Finegoldia magna	0	0.17
Thermus scotoductus	0	0.17
Mycolicibacterium neumannii	0	0.15
Actinomyces viscosus	0.01	0.13
Ezakiella coagulans	0.01	0.13
Acinetobacter ursingii	0	0.13
Peptoniphilus_C coxii	0	0.13
Prevotella colorans	0	0.13
Streptococcus anginosus	0	0.13

Streptococcus oralis_S	0	0.13
Staphylococcus auricularis	0.03	0.1
Staphylococcus capitis	0.03	0.1
Ralstonia pickettii	0.02	0.1
Pseudomonas_E lactis	0.01	0.1

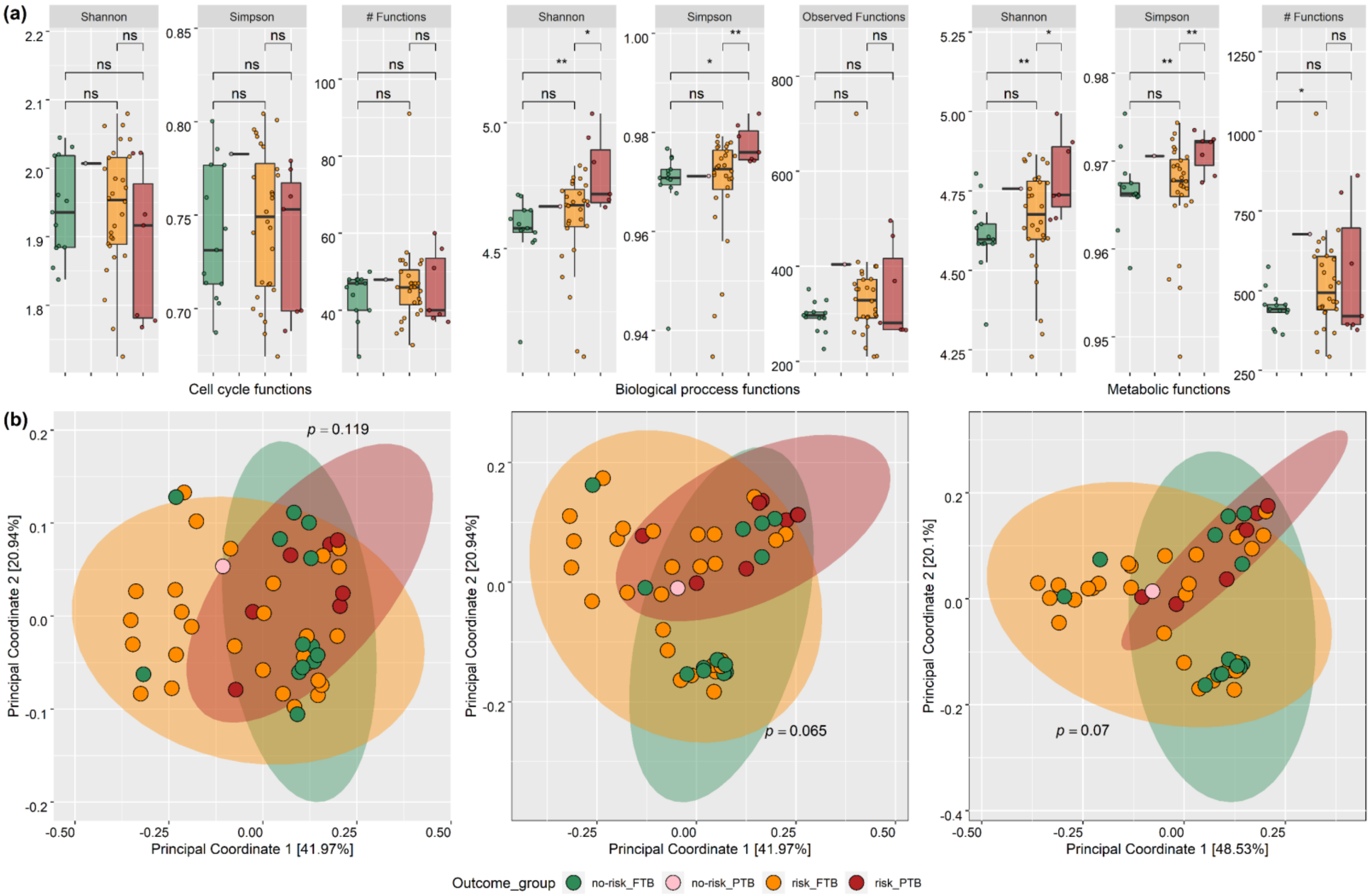

Supplementary Figure 1 | Functional diversity of the vaginal microbiome by outcome groups. Gene functions are divided into 3 higher level functional categories based on gene ontology classification with both alpha (a) and (b) beta diversity measures of identified content shown. Significant differences are highlighted by an asterisk where p value was < 0.05 as calculated by Kruskal-Wallis. Significant differences in beta diversity were determined by PERMANOVA analysis.
