# Supplementary material for: Shotgun sequencing of the vaginal microbiome reveals both a species and functional potential signature of preterm birth
Source: NPJ Biofilms Microbiomes. 2020 Nov 12;6:50. doi: 10.1038/s41522-020-00162-8 (PMC7665020; doi:10.1038/s41522-020-00162-8)
Supplement: Supplementary file 2 — Reporting Summary [file 41522_2020_162_MOESM2_ESM.pdf]

nature research

Corresponding author(s): Prof Paul Cotter

Last updated by author(s): Aug 5, 2020

Reporting Summary

Nature Research wishes to improve the reproducibility of the work that we publish. This form provides structure for consistency and transparency in reporting. For further information on Nature Research policies, see our Editorial Policies and the Editorial Policy Checklist.

Statistics

For all statistical analyses, confirm that the following items are present in the figure legend, table legend, main text, or Methods section.

n/a	Confirmed	
☐	☒	The exact sample size (*n*) for each experimental group/condition, given as a discrete number and unit of measurement
☐	☒	A statement on whether measurements were taken from distinct samples or whether the same sample was measured repeatedly
☐	☒	The statistical test(s) used AND whether they are one- or two-sided<br>*Only common tests should be described solely by name; describe more complex techniques in the Methods section.*
☐	☒	A description of all covariates tested
☐	☒	A description of any assumptions or corrections, such as tests of normality and adjustment for multiple comparisons
☐	☒	A full description of the statistical parameters including central tendency (e.g. means) or other basic estimates (e.g. regression coefficient) AND variation (e.g. standard deviation) or associated estimates of uncertainty (e.g. confidence intervals)
☐	☒	For null hypothesis testing, the test statistic (e.g. *F*, *t*, *r*) with confidence intervals, effect sizes, degrees of freedom and *P* value noted<br>*Give P values as exact values whenever suitable.*
☒	☐	For Bayesian analysis, information on the choice of priors and Markov chain Monte Carlo settings
☒	☐	For hierarchical and complex designs, identification of the appropriate level for tests and full reporting of outcomes
☒	☐	Estimates of effect sizes (e.g. Cohen's *d*, Pearson's *r*), indicating how they were calculated

Our web collection on statistics for biologists contains articles on many of the points above.

Software and code

Policy information about availability of computer code

Data collection

Provide a description of all commercial, open source and custom code used to collect the data in this study, specifying the version used OR state that no software was used.

Data analysis

Raw sequencing data was basecalled using Illumina's bcl2fastq software (v 2.19) (https://support.illumina.com/sequencing/sequencing_software/bcl2fastq-conversion-software.html). TrimGalore (v 0.6.0) (http://www.bioinformatics.babraham.ac.uk/projects/trim_galore/), a perl wrapper for Cutadapt (v. 1.18)33 and FastQC (v. 0.11.8) (http://www.bioinformatics.babraham.ac.uk/projects/fastqc/), was used to remove adapter sequences and low-quality sequences using default parameters. Removal of human DNA contamination was performed by aligning all high-quality paired-end reads to the latest draft of the human genome (hg38) using Bowtie2 (v. 2.3.4) 34. The resulting SAM files were converted to BAM format and filtered to keep only unmapped paired-end reads using SAMtools 35. Bedtools 36 was used to convert the remaining reads from BAM to FASTQ format. Taxonomic assignment of paired-end reads was performed using Kraken237 alignment against the GTDB_54k database created by Meric et al. (https://github.com/rrwick/Metagenomics-Index-Correction). Functional profiling was performed using the HUMAnN2 pipeline (v. 2.8.1) 38. The gene families output were renormalized as copies per million reads (CPM) and regrouped according to Gene Ontology terms.
Data was visualised using both Graphpad Prism 6 and RStudio (R v.3.6.0). Heatmaps were generated using the 'ComplexHeatmap' package39 with samples clustered using the Pearson distance metric and columns split by kmeans clustering to visualise community state types, assigned based on previous reported definitions24. Plots for diversity analysis were generated using the ggplot2 package (https://cran.r-project.org/web/packages/ggplot2/index.html). Statistical analysis was carried out in R using the vegan package (https://cran.r-project.org/web/packages/vegan/index.html) and RVAideMemoire (https://cran.r-project.org/web/packages/RVAideMemoire/index.html). Multivariate Association with Linear Models 2 (MaAsLiN2, R V.1.2.0) was used to determine independent associations of species and functions with metadata factors.

For manuscripts utilizing custom algorithms or software that are central to the research but not yet described in published literature, software must be made available to editors and reviewers. We strongly encourage code deposition in a community repository (e.g. GitHub). See the Nature Research guidelines for submitting code & software for further information.

Data

Policy information about availability of data

All manuscripts must include a data availability statement. This statement should provide the following information, where applicable:

- Accession codes, unique identifiers, or web links for publicly available datasets
- A list of figures that have associated raw data
- A description of any restrictions on data availability

Sequence data have been deposited in the European Nucleotide Archive (ENA) under the study accession number PRJEB34536.

Field-specific reporting

Please select the one below that is the best fit for your research. If you are not sure, read the appropriate sections before making your selection.

☒ Life sciences ☐ Behavioural & social sciences ☐ Ecological, evolutionary & environmental sciences

For a reference copy of the document with all sections, see nature.com/documents/nr-reporting-summary-flat.pdf

Life sciences study design

All studies must disclose on these points even when the disclosure is negative.

Sample size	57 participants in a prospective cohort study
Data exclusions	*Describe any data exclusions. If no data were excluded from the analyses, state so OR if data were excluded, describe the exclusions and the rationale behind them, indicating whether exclusion criteria were pre-established.*
Replication	*Describe the measures taken to verify the reproducibility of the experimental findings. If all attempts at replication were successful, confirm this OR if there are any findings that were not replicated or cannot be reproduced, note this and describe why.*
Randomization	*Describe how samples/organisms/participants were allocated into experimental groups. If allocation was not random, describe how covariates were controlled OR if this is not relevant to your study, explain why.*
Blinding	*Describe whether the investigators were blinded to group allocation during data collection and/or analysis. If blinding was not possible, describe why OR explain why blinding was not relevant to your study.*

Reporting for specific materials, systems and methods

We require information from authors about some types of materials, experimental systems and methods used in many studies. Here, indicate whether each material, system or method listed is relevant to your study. If you are not sure if a list item applies to your research, read the appropriate section before selecting a response.

Materials & experimental systems

n/a	Involved in the study	
☒	☐	Antibodies
☒	☐	Eukaryotic cell lines
☒	☐	Palaeontology and archaeology
☒	☐	Animals and other organisms
☐	☒	Human research participants
☒	☐	Clinical data
☒	☐	Dual use research of concern

Methods

n/a	Involved in the study	
☒	☐	ChIP-seq
☒	☐	Flow cytometry
☒	☐	MRI-based neuroimaging

Human research participants

Policy information about studies involving human research participants

Population characteristics	Pregnant women over the age of 18, with a risk of preterm birth due to either prior preterm birth or LLETZ procedure. A control group of pregnant women over the age of 18 with no known risk factors for preterm birth. Exclusion criteria were women currently on antibiotic treatment.
Recruitment	Participants in the study were recruited from women attending the preterm birth clinic at The National Maternity Hospital Dublin, Ireland.
Ethics oversight	Institutional ethics approval by the National Maternity Hospital Research Ethics Committee

Note that full information on the approval of the study protocol must also be provided in the manuscript.
